# Exploration of Pyrazolo[1,5‐*a*]pyrimidines as Membrane‐Bound Pyrophosphatase Inhibitors

**DOI:** 10.1002/cmdc.202100392

**Published:** 2021-10-12

**Authors:** Niklas G. Johansson, Loïc Dreano, Keni Vidilaseris, Ayman Khattab, Jianing Liu, Arthur Lasbleiz, Orquidea Ribeiro, Alexandros Kiriazis, Gustav Boije af Gennäs, Seppo Meri, Adrian Goldman, Jari Yli‐Kauhaluoma, Henri Xhaard

**Affiliations:** ^1^ Drug Research Program, Division of Pharmaceutical Chemistry and Technology, Faculty of Pharmacy University of Helsinki P.O. Box 56 (Viikinkaari 5 E) 00014 Helsinki Finland; ^2^ Department of Biosciences, Division of Biochemistry University of Helsinki P.O. Box 56 (Viikinkaari 9) 00014 Helsinki Finland; ^3^ Malaria Research Laboratory, Translational Immunology Research Program, Department of Bacteriology and Immunology, Haartman Institute University of Helsinki P.O. Box 21 (Haartmaninkatu 3) 00014 Helsinki Finland; ^4^ School of Biomedical Sciences and Astbury Centre for Structural Molecular Biology University of Leeds, Clarendon Way Leeds LS2 9JT UK

**Keywords:** membrane-bound pyrophosphatase, antiprotozoal agents, pyrazolo[1,5-*a*]pyrimidine, inhibitor, drug discovery

## Abstract

Inhibition of membrane‐bound pyrophosphatase (mPPase) with small molecules offer a new approach in the fight against pathogenic protozoan parasites. mPPases are absent in humans, but essential for many protists as they couple pyrophosphate hydrolysis to the active transport of protons or sodium ions across acidocalcisomal membranes. So far, only few nonphosphorus inhibitors have been reported. Here, we explore the chemical space around previous hits using a combination of screening and synthetic medicinal chemistry, identifying compounds with low micromolar inhibitory activities in the *Thermotoga maritima* mPPase test system. We furthermore provide early structure‐activity relationships around a new scaffold having a pyrazolo[1,5‐*a*]pyrimidine core. The most promising pyrazolo[1,5‐*a*]pyrimidine congener was further investigated and found to inhibit *Plasmodium falciparum* mPPase in membranes as well as the growth of *P. falciparum* in an *ex vivo* survival assay.

## Graphical abstract

**Chemical space exploration**: Novel low micromolar membrane‐bound pyrophosphatase (mPPase) inhibitors based on an exploratory screening against *Thermotoga maritima* mPPase followed by early SARs around a pyrazolo[1,5‐*a*]pyrimidine scaffold. Compound **17** **a** retained activity against *Plasmodium falciparum* mPPase in membranes, and inhibited parasite growth *ex vivo*.

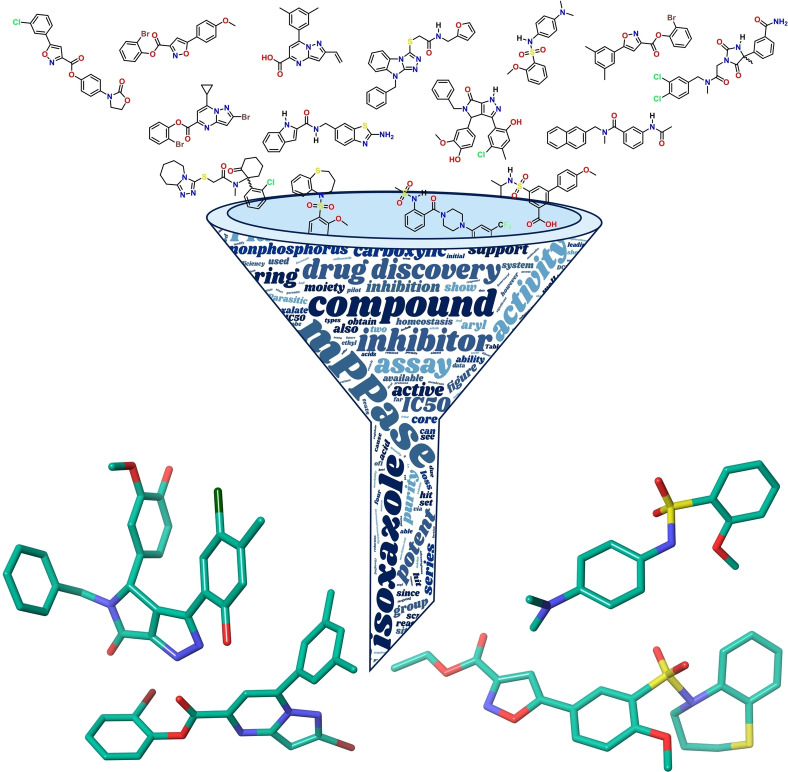

## Introduction

Parasitic human diseases, such as malaria, leishmaniasis, trypanosomiasis and toxoplasmosis, represent a severe global health concern. The life cycles of protozoan parasites, including *Plasmodium* spp., *Leishmania* spp., *Trypanosoma* spp. and *Toxoplasma* spp., are rather complex and typically comprise of transitions between hosts and vectors as well as intracellular and extracellular environments. To survive these changes, the protozoan cell must be able to adjust e. g. for fluctuating osmotic pressures. The key mechanism involves membrane‐bound pyrophosphatases (mPPases).^[1]^ These enzymes are located in the cell membrane of bacteria and archaea, but can also be found in the protist acidocalcisome, and in the Golgi apparatus and/or the vacuole of plants and algae.[^2^, ^3^, ^4^, ^5^, ^6^, ^7^, ^8^] mPPases are important for many protists by acting as ion pumps through pyrophosphate hydrolysis.[^9^, ^10^, ^11^, ^12^] As shown by knock‐out and knock‐down studies in *P. falciparum*,^[13]^ *T. brucei* ^[14]^ and *T. gondii*,^[15]^ mPPases are required for maintaining *in* *vitro* asexual blood stage growth, acidocalcisome acidification and parasitic virulence. Moreover, *T. gondii* intracellular proliferation was retarded by mPPase‐inhibiting bisphosphonate derivatives.^[16]^ In recent years, various crystal structures of mung bean *Vigna radiata*[^17^, ^18^] and hyperthermophilic bacterium *Thermotoga maritima*[^17^, ^19^, ^20^] mPPases have been solved by us and others.

mPPases are promising therapeutic targets as they play an important role in the lifecycle of many protists but do not exist in multicellular animals.[^9^, ^10^, ^11^, ^12^] In addition, their three‐dimensional structures have been solved in diverse conformations, which makes them amenable to structure‐based design. Only a few small molecule inhibitors have been however reported so far. A main class is formed by nonhydrolyzable pyrophosphate (PP_i_) analogues, which have the drawback of interfering with several human enzymes hydrolyzing or producing PP_i_. Examples include ectonucleotide pyrophosphatases, phosphodiesterases and inorganic pyrophosphatases.^[21]^

**Figure 1:**
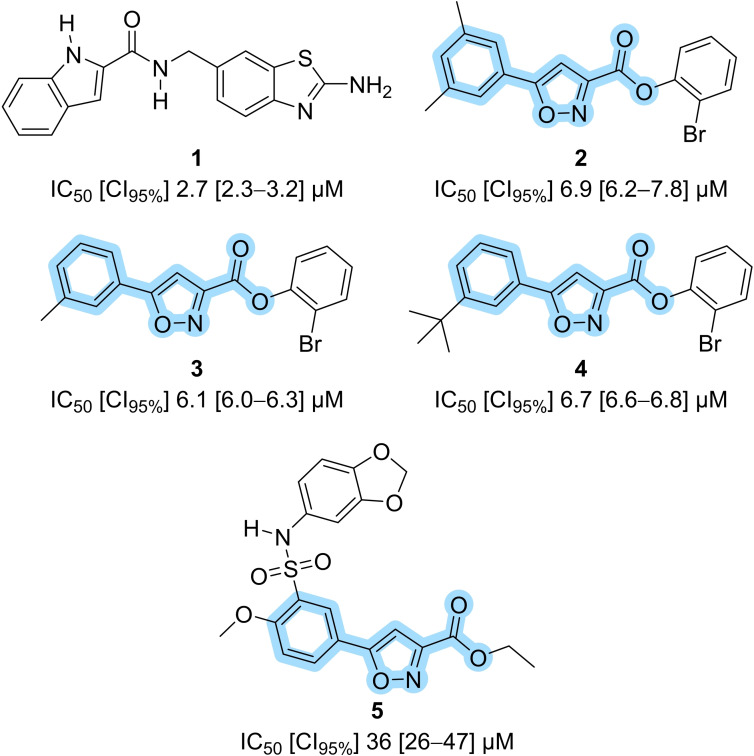
Selected examples of nonphosphorus mPPase inhibitors previously reported.[^20^, ^24^] Blue highlight, common substructure used for similarity searches in this manuscript; IC_50_, half maximal inhibitory concentration; CI_95%_, half maximal inhibitory concentration expressed as a 95 % confidence interval (given in square brackets).

Previously, we developed a 96‐well plate *in* *vitro* screening assay using thermostable *Thermotoga maritima* mPPase (TmPPase) as a model enzyme, and discovered several classes of nonphosphorus inhibitors.[^22^, ^23^] By screening of commercially available compounds as well as synthetic medicinal chemistry, we explored and identified several low micromolar hits (compounds **1**–**5**; Figure 1).^[24]^ Among those, the isoxazole‐based compound **2** retained activity *ex vivo* against *P. falciparum*.^[24]^ The binding location of the isoxazoles, whether at the catalytic site or elsewhere, could not be determined experimentally. In contrast, we have demonstrated using X‐ray crystallography that the 2‐aminobenzothiazole **1** binds allosterically outside the catalytic side, near the so‐called exit channel (PDB code: 6QXA).^[20]^

Here, starting from **2** and its congeners, we aimed to identify novel molecular scaffolds that would lead to gain in binding affinity. These could be translated into therapeutic molecules but could also have a higher probability to be co‐crystallized at the mPPase catalytic site, which so far has eluded us. Following a screening of 52 compounds, we set to explore the pyrazolo[1,5‐*a*]pyrimidine core. An advantage of swapping the central core structure is that it would allow further chemical expansion of the 2‐position in pyrazolo[1,5‐*a*]pyrimidines via Suzuki coupling or other coupling methods. Furthermore, pyrazolo[1,5‐*a*]pyrimidines^[25]^ have been reported to have antibacterial activity against *Mycobacterium tuberculosis*,^[26]^ antiparasitic activity including antileishmanial,^[27]^ antimalarial (*P. falciparum* dihydroorotate dehydrogenase inhibitors)^[28]^ and antitrypanosomal,^[27]^ as well as antiviral activity against HIV.^[29]^

## Results and Discussion

### Exploration of the isoxazole and sulfonamide space through screening

We started from the previously identified 5‐arylisoxazole‐3‐carboxylate core present in **2**–**5** (Figure 1, highlighted in blue). Using KNIME,^[30]^ we did a substructure search for commercial analogues in the ZINC12 database^[31]^ From our experience, computational methods (including docking simulations) do not offer a robust and/or reliable mean of prioritization at the mPPase. Therefore, we manually picked compounds from a diverse collection provided by a single vendor (Ambinter, 174 matches for the substructure query). Out of the search results, we selected fifteen isoxazoles for testing *in* *vitro*. For all compounds in this manuscript, we tested activity using a 96‐well based assay that detects the inhibition of TmPPase.[^22^, ^23^, ^24^]

**Figure 2:**
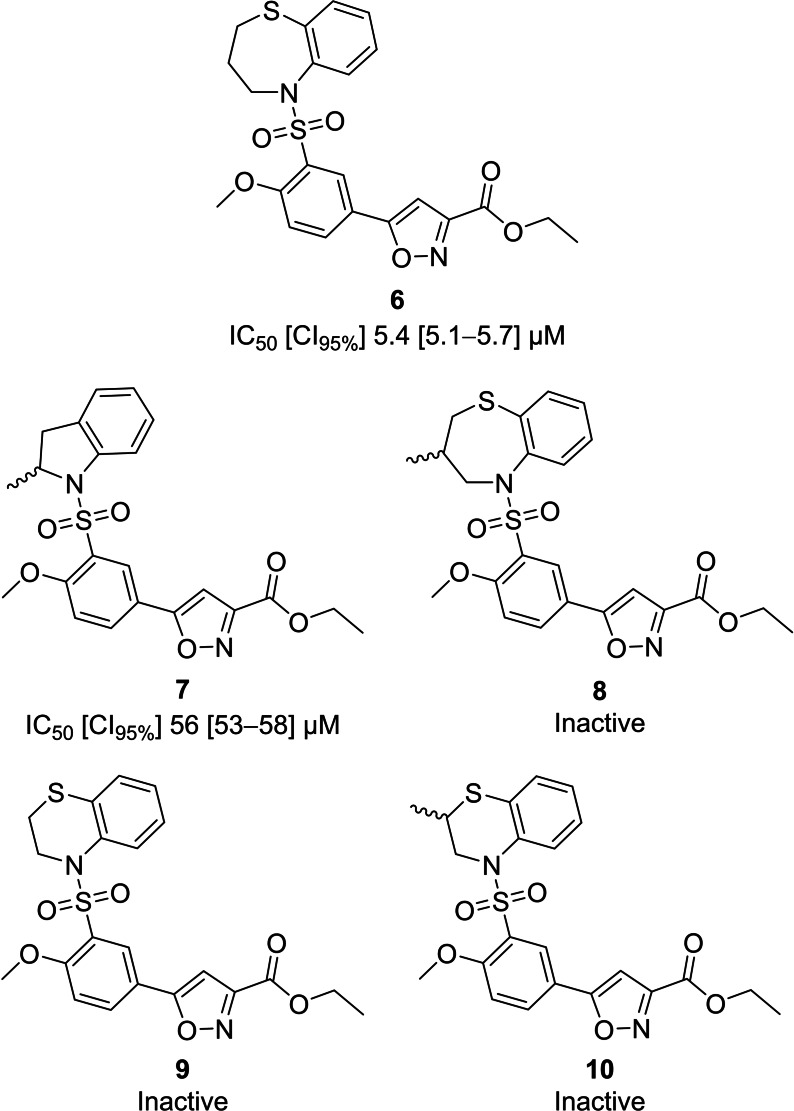
Top two compounds and some of their inactive analogues from the substructure‐based search.

In this first set (Supporting Information, Table S1 and S2), eleven of the fifteen isoxazoles had a sulfonamide‐linked 2‐methoxyphenyl moiety. The sulfonamides **6** and **7** (Figure 2) were the two best compounds with **6** (IC_50_=5.4 μM) being one of the most potent TmPPase inhibitors found this far. Interestingly, in comparison with **7–10**, the sulfonamide‐linked 2,3,4,5‐tetrahydrobenzo[*b*][1,4]thiazepine moiety found in **6** is critical for mPPase activity. Even the simple addition of a methyl group (as in **8**) was not tolerated. Also, minor alterations of the seven‐membered ring (as in **9** and **10**) caused the loss of all TmPPase inhibitory activity. Other ring modifications also proved unsuccessful (Table S2). Because of the sharp, difficult to rationalize, structure‐activity relationships of the isoxazole‐sulfonamide core, we in this manuscript decided to pursue other scaffolds (see below).

**Figure 3:**
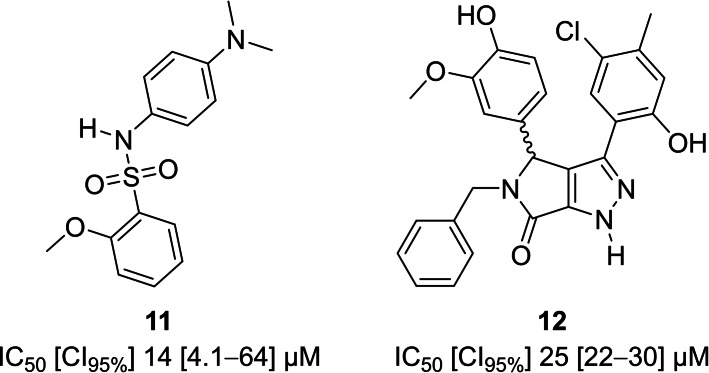
Best hits from the readily available compound set.

We also tested another set of 18 sulfonamides (Table S3) and 19 amides (Table S4) that were readily available to us and thus were candidates for repurposing. These compounds share some chemical features with **2**, with many having a central polar core bearing N and/or O atoms surrounded by more hydrophobic/aromatic functional groups. Of this set, compounds **11** and **12** (Figure 3) were the most potent with IC_50_ values below 25 μM. Sulfonamide **11** (IC_50_=14 μM; ligand efficiency (LE)=0.23) share the 2‐methoxyphenyl‐sulfonamide moiety of **6** but lacks its isoxazole core, and therefore shows potential for further exploration. Compound **12** (IC_50_=25 μM) is structurally different from the hitherto most potent nonphosphorus TmPPase inhibitors and thus was picked as a template for further design. Compound **12** was the only compound from this subset with a 4,5‐dihydropyrrolo[3,4‐*c*]pyrazol‐6(1*H*)‐one core.

### Chemical exploration of the pyrazolo[1,5‐*a*]pyrimidine core

Next, we decided to further explore the chemical space around the recently discovered 4,5‐dihydropyrrolo[3,4‐*c*]pyrazol‐6(1*H*)‐one scaffold, using combinations of substituents already found to be favorable for activity. We nonetheless decided to swap the original bicyclic scaffold with a related nitrogen‐rich, pyrazolo[1,5‐*a*]pyrimidine core. This approach was synthetically more achievable, allowing easy modifications at three positions. In addition, the synthetic exploration could take advantage of readily available starting materials, such as various aromatic or aliphatic ketones. The scaffold change can be rationalized by comparing the pharmacophores of **17** **a** with **2** and **12** (Supporting Information, Figure S1 and S2).

**Scheme 1:**
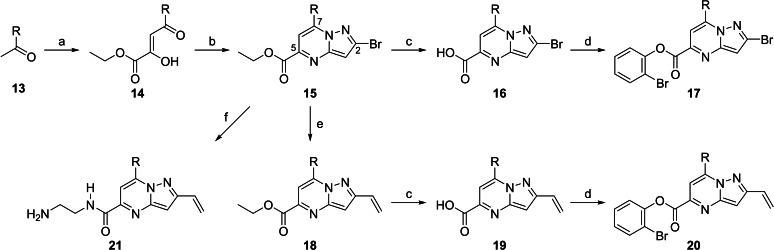
Synthesis of compounds **14**–**21**. Reagents and conditions: (a) *t*‐BuOK, diethyl oxalate, THF, rt, 1 h, 87–88 %; (b) 5‐Bromo‐1*H*‐pyrazol‐3‐amine; HCl (aq), EtOH, mw, 78 °C, 30 min, 64–81 %; (c) LiOH, EtOH/H_2_O, rt, overnight, 89–96 %; (d) 2‐Bromophenol, HATU, DIPEA, DMF, rt, overnight, 36–61 %; (e) Potassium vinyltrifluoroborate, Et_3_N, Pd(dppf)Cl_2_, EtOH, mw, 125 °C, 15 min, 53–56 %; (f) Potassium vinyltrifluoroborate, ethylenediamine, Pd(dppf)Cl_2_, *n*‐PrOH, mw, 125 °C, 15–30 min, 49–87 %.

We relied on a method reported by Childress et al.^[32]^ as we could adapt the first two steps of their synthesis route to access the key intermediate **15** (Scheme 1). Crossed condensation at room temperature of commercially available 3,5‐dimethylacetophenone or acetylcyclopropane with diethyl oxalate and potassium *tert*‐butoxide in THF gave **14**. Subsequent ring condensation of **14** with 3‐bromo‐1*H*‐pyrazol‐5‐amine in refluxing ethanol resulted in the formation of pyrazolo[1,5‐*a*]pyrimidine **15**. Hydrolysis of the formed ethyl ester **15** using lithium hydroxide in a mixture of ethanol and water gave the corresponding carboxylic acid **16**, which was reacted with 2‐bromophenol and 1‐[bis(dimethylamino)methylene]‐1*H*‐1,2,3‐triazolo[4,5‐*b*]pyridinium 3‐oxide hexafluorophosphate (HATU) and 2‐bromophenol to give the desired 2‐bromophenyl carboxylate **17**.

The bromine substituent in the 2‐position of **15** could serve as a coupling handle for further exploration of the chemical space by various Pd‐mediated cross‐coupling reactions. Suzuki coupling of ethyl ester **15** in *n*‐propanol with commercially available potassium vinyltrifluoroborate was done under microwave conditions. The product obtained, **18**, could be transformed into the corresponding 2‐bromophenyl ester **20** following the same hydrolysis and esterification procedures used for **16** and **17**, respectively. However, changing trimethylamine used in the Suzuki coupling to ethylenediamine serendipitously led to both Suzuki coupling and amidation occurring in the same reaction mixture, yielding **21** in a single step.

### Biological activity of the pyrazolo[1,5‐*a*]pyrimidine core

We started our exploration by taking inspiration from **12** (IC_50_=25 μM) and changing the isoxazole core of **2** (IC_50_=6.9 μM) to the corresponding pyrazolo[1,5‐*a*]pyrimidine analogue **17** **a** (Table 1). This scaffold change was relatively well tolerated, with just a 2‐fold loss in activity compared to **2**. As previously noticed for the isoxazole series,^[24]^ the corresponding carboxylic acid **16** **a** was inactive, but the ethyl ester **15** **a** retained weak activity.

**Table 1:** Activities of the pyrazolo[1,5‐*a*]pyrimidine series.

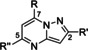									
compound	R	R^’^	R^’’^	IC_50_ [CI_95%_]	compound	R	R^’^	R^’’^	IC_50_ [CI_95%_]
**15** **a**	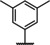	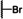	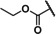	120 [110–140] μM	**15** **b**	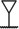	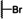	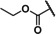	150 [130–180] μM
**16** **a**	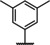	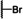	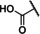	NA [NA–NA] μM	**16** **b**	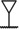	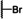	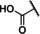	NA [NA–NA] μM
**17** **a**	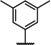	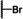	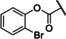	14 [13–15] μM	**17** **b**	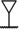	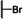	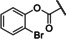	NA [NA–NA] μM
**18** **a**	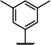	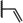	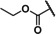	54 [51–58] μM	**18** **b**	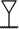	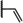	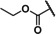	NA [NA–NA] μM
**19** **a**	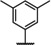	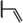	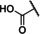	14 [13–15] μM	**19** **b**	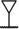	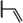	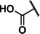	NA [NA–NA] μM
**20** **a**	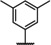	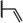	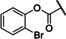	18 [17–19] μM	**20** **b**	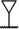	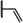	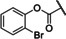	NA [NA–NA] μM
**21** **a**	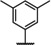	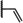	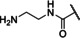	72 [61–85] μM	**21** **b**	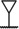	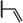	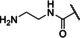	260 [190–360] μM

Since the central pyrazolo[1,5‐*a*]pyrimidine core is bulkier than the original isoxazole moiety (the molecular weight of **17** **a** is approximately 500 Da), we tried to introduce lighter substitutions. The exchange of the R‐group at the 7‐position, from a 3,5‐dimethylphenyl group to a cyclopropyl ring, was only slightly effective for the ethyl carboxylate **15** **b**. Furthermore, it proved completely unsuccessful for the carboxylic acid **16** **b** and the 2‐bromophenyl carboxylate **17** **b**. Similarly, replacing the R‐substituent with other phenyl moieties than the original 3,5‐dimethylphenyl substituent (unsubstituted or bearing electron withdrawing/donating groups), were generally not well tolerated for the isoxazole derivatives.^[24]^

We next studied further functionalization of the 2‐position in pyrazolo[1,5‐*a*]pyrimidines. Bromine atoms are very useful in X‐ray crystallography (due to their anomalous scattering, which can aid in identifying the presence of the compound as well as its orientation in low‐resolution, 3.5 Å or worse). Additionally, aryl bromides are highly useful e. g. in Suzuki coupling with various organoboron substrates. We introduced a well‐accepted vinyl group at the 5‐position, showing nearly no loss of activity for **20** **a** in comparison to the hitherto best pyrazolo[1,5‐*a*]pyrimidine **17** **a** (IC_50_=14 μM). Moreover, there was a 2.2‐fold improvement in the inhibition comparing the ethyl ester **18** **a** to the 2‐bromo analogues **15** **a**. Interestingly, the 2‐vinyl‐substituted carboxylic acid **19** **a** (IC_50_=14 μM) was as active as the best pyrazolo[1,5‐*a*]pyrimidines and superior to its inactive 2‐bromo analogue **16** **a**. As presented above, the corresponding 2‐vinyl‐substituted cyclopropyl analogues **18** **b**–**20** **b** were all inactive. In the same way, the 3,5‐dimethylphenyl‐substituted amide **21** **a** showed 3.6‐fold higher inhibition than the corresponding cyclopropyl‐substituted analogue **21** **b**.

### Follow‐up studies of 12, 17 a, 19 a and 20 a

In order to rule out a cause of false positives, colloidal aggregation in the TmPPase model assay was evaluated for compounds **17** **a**, **19** **a** and **20** **a** at six concentrations (100 μM, 50 μM, 20 μM, 10 μM, 1 μM, and 0.1 μM) using the assay conditions (Figure S3). Compounds **17** **a** and **20** **a** showed aggregate formation at concentrations above 20 μM, which is above their IC_50_ values. Compound **19** **a** showed no detectable aggregation.

**Figure 4:**
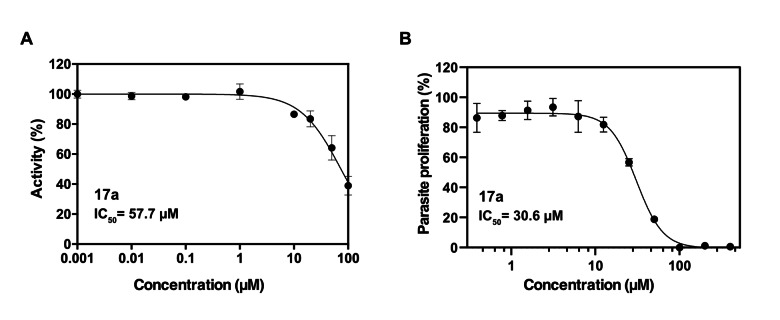
(A) Inhibition of *P. falciparum* mPPase (PfPPaseVP‐1) by **17** **a**. (B) Effect of **17** **a** on *P. falciparum* growth. All data are shown as mean ±SD in three replicates.

Further hit validation was done against the purified mPPase from *P. falciparum* (PfPPase‐VP1) expressed in baculovirus‐infected insect cells. Compound **17** **a** was able to inhibit the PfPPase‐VP1 activity with an IC_50_ of 58 μM (Figure 4A). Compounds **19** **a** and **20** **a** had lower inhibition activities with IC_50_ values of 130 μM and 74 μM, respectively (Figure S5). Overall, these compounds have higher IC_50_ values for PfPPase‐VP1 than for TmPPase. In a survival assay in erythrocytes culture **17** **a** was able to inhibit the growth of *P. falciparum* with an IC_50_ of 31 μM (Figure 4B), better than its inhibition on the PfPPase‐VP1. This could mean that the compound inhibits other proteins in the parasite, e. g. through soluble pyrophosphatase, or via some other mechanism, which could be linked to colloidal aggregation. Interestingly, compound **12** was able to inhibit the growth of *P. falciparum* with the IC_50_ of 3.6 μM (Figure S6) even though the activity on PfPPase‐VP1 (Figure S4) was comparably weak. No hemolysis of human erythrocytes was observed, suggesting no significant cytotoxicity.^[33]^

## Conclusion

Altogether, this manuscript presents novel scaffolds with potential for further exploration in the drug discovery against parasitic diseases. Using a screening approach (52 compounds) together with a medicinal chemistry exploration (14 compounds) and the TmPPase test system, we discovered new TmPPase inhibitors: the 4,5‐dihydropyrrolo[3,4‐*c*]pyrazol‐6(1*H*)‐one core (compound **12**) and pyrazolo[1,5‐*a*]pyrimidines (**17** **a**, **19** **a** and **20** **a**). We explored the SARs around this latter core and maintained low micromolar activity (IC_50_=14–18 μM) for three of the synthesized pyrazolo[1,5‐*a*]pyrimidines. Molecular modelling suggests that the substrate binding sites are highly conserved in many protozoan pathogens, which should allow transferability of the findings.[^11^, ^34^] Indeed, compound **17** **a** binds to the PfPPase‐VP1 with an IC_50_ of 58 μM and inhibits parasite growth.

## Experimental Section

### Computational methods

Pharmacophore modelling was conducted using the Schrödinger Maestro suite.^[35]^ Substructure searches were conducted from the ZINC12 database^[31]^ (clean drug‐like subset; 13,195,609 compounds; downloaded on 2018.11.11). Ligand efficiencies were computed using the pIC_50_ and the “Heavy Atom Count“ normalization method with Accelrys's Discovery Studio.^[36]^

### Chemistry

**General experimental methods**: All chemicals were available from commercial vendors and used without any further purification. Anhydrous reactions were conducted in oven‐dried (130 °C, >24 h) glassware that were purged with argon prior to use. Microwave reactions were done in sealed reaction vials using a Biotage^®^ Initiator^+^ instrument (Uppsala, Sweden). The progress of the reactions was monitored using thin‐layer chromatography on silica gel 60‐F_254_ aluminum plates and visualized by a dual short/long wave (254/366 nm) UV lamp. Combined organic solutions from extractions were dried over anhydrous Na_2_SO_4_, filtered and concentrated with a rotary evaporator at reduced pressure. Flash SiO_2_ column chromatography was performed with automated high performance flash chromatography, Biotage^®^ Isolera™ Spektra Systems with ACI™ and Assist (ISO‐1SW Isolera One) equipped with a variable UV‐VIS (200–800 nm) photodiode array (Uppsala, Sweden) using SNAP KP‐Sil/Ultra 10, 25, 50 or 100 g cartridges and the indicated mobile phase gradient. The reactions were not optimized and all the yields are given for purified products.

The synthesized products were characterized by NMR and MS analysis. ^1^H and ^13^C NMR spectra were acquired at 298 K on a Bruker Ascend 400 MHz‐Avance III HD NMR spectrometer (Bruker Corporation, Billerica, MA, USA). Chemical shifts (*δ*) are reported in parts per million (ppm) relative to the residual solvent signals: CDCl_3_ 7.26 and 77.16 ppm, CD_3_OD 3.31 and 49.00 ppm for ^1^H and ^13^C NMR, respectively. Multiplicities are indicated as bs (broad singlet), s (singlet), d (doublet), t (triplet), q (quartet), dd (doublet of doublets), tt (triplet of triplets) and m (multiplet). Purity of the final compounds (>95 %) was confirmed by LC–MS using a Waters Acquity^®^ UPLC system (Waters, Milford, MA, USA) equipped with an Acquity PDA detector, a Waters Synapt G2 HDMS mass spectrometer (Waters, Milford, MA, USA) and an Acquity UPLC^®^ BEH C18 column (1.7 μm, 50 mm×2.1 mm, Waters, Ireland). Mass range was set from 100 to 600 Da. High resolution mass (HRMS‐ESI) data was reported for the calculated and experimentally found molecular ions [M+H]^+^ or [M−H]^−^.

**General procedure for synthesis of compound 14**: The synthesis of compound **14** was adapted from a previously described method.^[32]^ In brief, *t*‐BuOK (2 equiv) was dissolved in anhydrous THF (1.2 mL/mmol) under argon, followed by addition of diethyl oxalate (2 equiv) and the mixture was stirred for 30 min. Then, ketone **13** (1 equiv) dissolved in anhydrous THF (2.2 mL/mmol) was added dropwise and stirred for 1 h. The reaction was quenched by addition of a 1 M solution of HCl in H_2_O (3.0 mL/mmol).

**Ethyl 4‐(3,5‐dimethylphenyl)‐2‐hydroxy‐4‐oxobut‐2‐enoate (14** **a)**: 1‐(3,5‐Dimethylphenyl)ethan‐1‐one (2.0 g, 13 mmol) was used to give **14** **a** (2.9 g, 88 %) as a yellow solid. ^1^H NMR (400 MHz, CDCl_3_): *δ*=7.59 (s, 2H), 7.23 (s, 1H), 7.04 (s, 1H), 4.40 (q, *J*=7.1 Hz, 2H), 2.38 (s, 6H), 1.41 (t, *J*=7.1 Hz, 3H), 1H not observed (exchangeable); ^13^C NMR (101 MHz, CDCl_3_): *δ*=191.3, 169.6, 162.4, 138.7, 135.7, 135.0, 125.8, 98.2, 62.7, 21.3, 14.2.

**Ethyl 4‐cyclopropyl‐2‐hydroxy‐4‐oxobut‐2‐enoate (14** **b)**: 1‐Cyclopropylethan‐1‐one (1.4 g, 17 mmol) was used to give **14** **b** (2.8 g, 87 %) as a pale yellow liquid. ^1^H NMR (400 MHz, CDCl_3_): *δ*=6.46 (s, 1H), 4.33 (q, *J*=7.1 Hz, 2H), 1.87 (tt, *J*=7.8, 4.5 Hz, 1H), 1.35 (t, *J*=7.1 Hz, 3H), 1.22–1.17 (m, 2H), 1.08–1.02 (m, 2H), 1H not observed (exchangeable); ^13^C NMR (101 MHz, CDCl_3_): *δ*=204.7, 163.2, 162.4, 102.2, 62.5, 20.8, 14.1, 12.3.

**General procedure for synthesis of compound 15**: The synthesis of compound **15** was adapted from a previously reported method.^[32]^ Briefly, to a solution of **14** (1 equiv) in EtOH (5.0 mL/mmol) were added a catalytic amount of a 1 M solution of HCl in H_2_O (5 drops) and 5‐bromo‐1*H*‐pyrazol‐3‐amine (1.25 equiv). The reaction mixture heated by microwave irradiation at 78 °C for 30 min, followed by filtration once the product had precipitated.

**Ethyl 2‐bromo‐7‐(3,5‐dimethylphenyl)pyrazolo[1,5‐*a*]pyrimidine‐5‐carboxylate (15** **a)**: Compound **14** **a** (1.0 g, 4.1 mmol) was used to give **15** **a** (1.2 g, 81 %) as a pale yellow solid. ^1^H NMR (400 MHz, CDCl_3_): *δ*=7.65 (s, 2H), 7.64 (s, 1H), 7.22 (s, 1H), 7.02 (s, 1H), 4.54 (q, *J*=7.1 Hz, 2H), 2.42 (s, 6H), 1.48 (t, *J*=7.1 Hz, 3H); ^13^C NMR (101 MHz, CDCl_3_): *δ*=164.0, 149.8, 147.6, 147.3, 138.7, 136.1, 133.7, 129.9, 127.1, 107.4, 102.1, 62.9, 21.5, 14.4; HRMS (ESI) *m/z*: [M+H]^+^ calcd for C_17_H_17_BrN_3_O_2_ 374.0504, found 374.0503.

**Ethyl 2‐bromo‐7‐cyclopropylpyrazolo[1,5‐*a*]pyrimidine‐5‐carboxylate (15** **b)**: Compound **14** **b** (0.77 g, 4.2 mmol) was used to give **15** **b** (0.82 g, 64 %) as a white solid. ^1^H NMR (400 MHz, CDCl_3_): *δ*=7.05 (s, 1H), 6.93 (s, 1H), 4.49 (q, *J*=7.1 Hz, 2H), 2.91 (tt, *J*=8.5, 5.3 Hz, 1H), 1.47–1.38 (m, 5H), 1.19–1.13 (m, 2H); ^13^C NMR (101 MHz, CDCl_3_): 164.1, 153.3, 148.7, 147.2, 135.8, 101.8, 101.1, 62.9, 14.4, 10.8, 10.7; HRMS (ESI) *m/z*: [M+H]^+^ calcd for C_12_H_13_BrN_3_O_2_ 310.0191, found 310.0190.

**General procedure for synthesis of compounds 16 and 19**: The synthesis of compounds **16** and **19** were done according to a method described elsewhere.^[24]^ In brief, a mixture of the ethyl ester (1 equiv) and LiOH (2 equiv) in a 4 : 1 ratio of EtOH/H_2_O (75 mL/mmol) was stirred for 16 h (3 d for **16** **b**), followed by back‐extraction.

**2‐Bromo‐7‐(3,5‐dimethylphenyl)pyrazolo[1,5‐*a*]pyrimidine‐5‐carboxylic acid (16** **a)**: Compound **15** **a** (0.30 g, 0.80 mmol) was used to give **16** **a** (0.25 g, 89 %) as a pale yellow solid. ^1^H NMR (400 MHz, CD_3_OD): *δ*=7.69 (s, 2H), 7.67 (s, 1H), 7.28 (s, 1H), 7.01 (s, 1H), 2.43 (s, 6H); ^13^C NMR (101 MHz, CD_3_OD): *δ*=166.5, 151.0, 149.7, 149.0, 139.8, 136.7, 134.3, 131.3, 128.2, 108.4, 102.0, 21.4; HRMS (ESI) *m/z*: [M+H]^+^ calcd for C_15_H_13_BrN_3_O_2_ 346.0191, found 346.0196.

**2‐Bromo‐7‐cyclopropylpyrazolo[1,5‐*a*]pyrimidine‐5‐carboxylic acid (16** **b)**: Compound **15** **b** (0.15 g, 0.49 mmol) was used to give **16** **b** (0.12 g, 90 %) as a white solid. ^1^H NMR (400 MHz, CD_3_OD): *δ*=7.13 (s, 1H), 6.91 (s, 1H), 2.98 (tt, *J*=8.4, 5.1 Hz, 1H), 1.51–1.44 (m, 2H), 1.25–1.19 (m, 2H); ^13^C NMR (101 MHz, CD_3_OD): *δ*=163.5, 155.1, 147.5, 145.5, 136.5, 101.3, 99.5, 11.4; 11.1; HRMS (ESI) *m/z*: [M+H]^+^ calcd for C_10_H_9_BrN_3_O_2_ 281.9878, found 281.9876.

**7‐(3,5‐Dimethylphenyl)‐2‐vinylpyrazolo[1,5‐*a*]pyrimidine‐5‐carboxylic acid (19** **a)**: Compound **18** **a** (0.23 g, 0.71 mmol) was used to give **19** **a** (0.20 g, 96 %) as a yellow solid. ^1^H NMR (400 MHz, CD_3_OD): *δ*=7.63 (s, 2H), 7.53 (s, 1H), 7.19 (s, 1H), 7.00 (s, 1H), 6.85 (dd, *J*=17.7, 11.0 Hz, 1H), 6.12 (dd, *J*=17.7, 1.3 Hz, 1H), 5.56 (dd, *J*=11.0, 1.3 Hz, 1H), 2.37 (s, 6H); ^13^C NMR (101 MHz, CD_3_OD): *δ*=166.5, 157.8, 150.8, 148.7, 148.2, 139.6, 134.0, 131.7, 130.2, 128.1, 120.3, 107.7, 96.0, 21.4; HRMS (ESI) *m/z*: [M+H]^+^ calcd for C_17_H_16_N_3_O_2_ 294.1242, found 294.1246.

**7‐Cyclopropyl‐2‐vinylpyrazolo[1,5‐*a*]pyrimidine‐5‐carboxylic acid (19** **b)**: Compound **18** **b** (0.10 g, 0.40 mmol) was used to give **19** **b** (0.88 mg, 96 %) as a pale yellow solid. ^1^H NMR (400 MHz, CD_3_OD): *δ*=6.98 (s, 1H), 6.83 (s, 1H), 6.81 (dd, *J*=17.8, 11.1 Hz, 1H), 6.07 (dd, *J*=17.8, 1.2 Hz, 1H), 5.52 (dd, *J*=11.1, 1.2 Hz, 1H), 2.78 (tt, *J*=8.4, 5.1 Hz, 1H), 1.37–1.29 (m, 2H), 1.17–1.10 (m, 2H); ^13^C NMR (101 MHz, CD_3_OD): *δ*=166.4, 157.4, 154.5, 149.4, 147.9, 130.1, 120.1, 101.7, 95.7, 11.5, 10.4; HRMS (ESI) *m/z*: [M+H]^+^ calcd for C_12_H_12_N_3_O_2_ 230.0930, found 230.0932.

**General procedure for synthesis of compounds 17 and 20**: The carboxylic acid (1 equiv) was dissolved in anhydrous DMF (3.3 mL/mmol) under argon, followed by *N*,*N*‐diisopropylethylamine (DIPEA; 2.5 equiv) and stirred for 15 min at room temperature. HATU (1.2 equiv) and 2‐bromophenol (1.3 equiv) were subsequently added and the mixture was stirred for 16 h. The mixture was diluted with EtOAc and washed once with a 0.05 M solution of HCl in H_2_O (2.5 equiv), three times with a half‐saturated solution of NaHCO_3_ in H_2_O, brine, dried with Na_2_SO_4_, filtered, evaporated and the residue was purified by flash chromatography with *n*‐hexane/EtOAc (1:0→0:1) as eluent.

**2‐Bromophenyl 2‐bromo‐7‐(3,5‐dimethylphenyl)pyrazolo[1,5‐*a*]pyrimidine‐5‐carboxylate (17** **a)**: Compound **16** **a** (0.051 g, 0.15 mmol) was used to give **17** **a** (0.027 g, 36 %) as a pale yellow solid. ^1^H NMR (400 MHz, CDCl_3_): *δ*=7.80 (s, 1H), 7.71–7.66 (m, 3H), 7.44–7.39 (m, 1H), 7.33 (dd, *J*=8.1, 1.6 Hz, 1H), 7.25–7.19 (m, 2H), 7.12 (s, 1H), 2.45 (s, 6H); ^13^C NMR (101 MHz, CDCl_3_): *δ*=161.7, 149.9, 148.4, 147.9, 145.8, 138.8, 136.5, 133.9, 133.7, 129.8, 128.9, 128.1, 127.2, 123.7, 116.2, 107.8, 102.6, 21.6; HRMS (ESI) *m/z*: [M+H]^+^ calcd for C_21_H_16_Br_2_N_3_O_2_ 499.9609, found 499.9611.

**2‐Bromophenyl 2‐bromo‐7‐cyclopropylpyrazolo[1,5‐*a*]pyrimidine‐5‐carboxylate (17** **b)**: Compound **16** **b** (0.050 g, 0.18 mmol) was used to give **17** **b** (0.043 g, 56 %) as a white solid. ^1^H NMR (400 MHz, CDCl_3_): *δ*=7.65 (dd, *J*=8.1, 1.5 Hz, 1H), 7.41–7.36 (m, 1H), 7.29 (dd, *J*=8.1, 1.5 Hz, 1H), 7.22–7.16 (m, 2H), 7.03 (s, 1H), 2.96 (tt, *J*=8.4, 5.2 Hz, 1H), 1.48–1.41 (m, 2H), 1.25–1.19 (m, 2H); ^13^C NMR (101 MHz, CDCl_3_): *δ*=161.8, 153.7, 148.7, 148.3, 145.7, 136.2, 133.6, 128.8, 128.0, 123.7, 116.1, 102.2, 101.5, 11.0, 10.9; HRMS (ESI) *m/z*: [M+H]^+^ calcd for C_16_H_12_Br_2_N_3_O_2_ 435.9296, found 435.9297.

**2‐Bromophenyl 7‐(3,5‐dimethylphenyl)‐2‐vinylpyrazolo[1,5‐*a*]pyrimidine‐5‐carboxylate (20** **a)**: Compound **19** **a** (0.033 g, 0.11 mmol) was used to give **20** **a** (0.022 g, 43 %) as a yellow solid. ^1^H NMR (400 MHz, CDCl_3_): *δ*=7.76 (s, 1H), 7.75 (s, 1H), 7.69 (dd, *J*=8.0, 1.6 Hz, 1H), 7.44–7.39 (m, 1H), 7.34 (dd, *J*=8.0, 1.6 Hz, 1H), 7.25–7.18 (m, 2H), 7.14 (s, 1H), 6.94 (dd, *J*=17.8, 11.0 Hz, 1H), 6.13 (dd, *J*=17.8, 1.2 Hz, 1H), 5.60 (dd, *J*=11.0, 1.2 Hz, 1H), 2.45 (s, 6H); ^13^C NMR (101 MHz, CDCl_3_): *δ*=162.1, 157.0, 150.0, 148.5, 147.6, 144.9, 138.6, 133.7, 133.5, 130.4, 129.2, 128.8, 128.0, 127.2, 123.8, 120.1, 116.3, 107.3, 96.5, 21.5 ; HRMS (ESI) *m/z*: [M+H]^+^ calcd for C_23_H_19_BrN_3_O_2_ 448.0661, found 448.0665.

**2‐Bromophenyl 7‐cyclopropyl‐2‐vinylpyrazolo[1,5‐*a*]pyrimidine‐5‐carboxylate (20** **b)**: Compound **19** **b** (0.034 g, 0.15 mmol) was used to give **20** **b** (0.035 mg, 61 %) as a yellow solid. ^1^H NMR (400 MHz, CDCl_3_): *δ*=7.66 (dd, *J*=8.0, 1.6 Hz, 1H), 7.42–7.36 (m, 1H), 7.30 (dd, *J*=8.0, 1.6 Hz, 1H), 7. 21–7.15 (m, 2H), 7.04 (s, 1H), 6.94 (dd, *J*=17.7, 11.0 Hz, 1H), 6.15 (dd, *J*=17.7, 1.2 Hz, 1H), 5.60 (dd, *J*=11.0, 1.2 Hz, 1H), 3.00 (tt, *J*=8.4, 5.2 Hz, 1H), 1.46–1.39 (m, 2H), 1.24–1.19 (m, 2H); ^13^C NMR (101 MHz, CDCl_3_): *δ*=162.2, 156.7, 153.3, 148.8, 148.5, 144.8, 133.6, 129.1, 128.8, 127.9, 123.8, 120.0, 116.2, 101.0, 96.4, 10.9, 10.5; HRMS (ESI) *m/z*: [M+H]^+^ calcd for C_18_H_15_BrN_3_O_2_ 384.0348, found 384.0348.

**General procedure for synthesis of compound 18**: The synthesis of compound **18** was adapted from a previously reported method.^[32]^ Briefly, the 2‐bromo pyrazolo[1,5‐*a*]pyrimidine **15** (1 equiv), potassium vinyltrifluoroborate (1.2 equiv), Et_3_N (2 equiv), and Pd(dppf)Cl_2_ (0.05 equiv) were dissolved in EtOH (7.5 mL/mmol) in a microwave vial and purged with argon for 10 min. The reaction mixture was heated by microwave irradiation at 125 °C for 15 min. Then, the reaction mixture was filtrated through Celite® and purified with automated flash chromatography with *n*‐hexane/EtOAc (1:0→0:1) as eluent.

**Ethyl 7‐(3,5‐dimethylphenyl)‐2‐vinylpyrazolo[1,5‐*a*]pyrimidine‐5‐carboxylate (18** **a)**: Compound **17** **a** (0.50 g, 1.3 mmol) was used to give **18** **a** (0.23 g, 53 %) as a yellow solid. ^1^H NMR (400 MHz, CDCl_3_): *δ*=7.70 (s, 2H), 7.60 (s, 1H), 7.21 (s, 1H), 7.05 (s, 1H), 6.90 (dd, *J*=17.7, 11.0 Hz, 1H), 6.09 (dd, *J*=17.7, 1.2 Hz, 1H), 5.56 (dd, *J*=11.0, 1.2 Hz, 1H), 4.54 (q, *J*=7.1 Hz, 2H), 2.43 (s, 6H), 1.48 (t, *J*=7.1 Hz, 3H); ^13^C NMR (101 MHz, CDCl_3_): *δ*=164.4, 156.6, 149.9, 147.3, 146.5, 138.5, 133.3, 130.6, 129.3, 127.2, 119.7, 106.9, 96.0, 62.7, 21.5, 14.5; HRMS (ESI) *m/z*: [M+H]^+^ calcd for C_19_H_20_N_3_O_2_ 322.1555, found 322.1554.

**Ethyl 7‐cyclopropyl‐2‐vinylpyrazolo[1,5‐*a*]pyrimidine‐5‐carboxylate (18** **b)**: Compound **17** **b** (0.40 g, 1.3 mmol) was used to give **18** **b** (0.19 g, 56 %) as a pale yellow solid. ^1^H NMR (400 MHz, CDCl_3_): *δ*=6.99 (s, 1H), 6.94 (s, 1H), 6.89 (dd, *J*=17.7, 11.0 Hz, 1H), 6.10 (dd, *J*=17.7, 1.2 Hz, 1H), 5.55 (dd, *J*=11.0, 1.2 Hz, 1H), 4.48 (q, *J*=7.1 Hz, 2H), 2.94 (tt, *J*=8.5, 5.2 Hz, 1H), 1.43 (t, *J*=7.1 Hz, 3H), 1.41–1.33 (m, 2), 1.19–1.11 (m, 2H); ^13^C NMR (101 MHz, CDCl_3_): *δ*=164.5, 156.3, 153.0, 148.7, 146.3, 129.2, 119.7, 100.6, 96.0, 62.6, 14.4, 10.7, 10.3; HRMS (ESI) *m/z*: [M+H]^+^ calcd for C_14_H_16_N_3_O_2_ 258.1242, found 258.1245.

**General procedure for synthesis of compound 21**: 2‐Bromo pyrazolo[1,5‐*a*]pyrimidine **15** (1 equiv), potassium vinyltrifluoroborate (1.2 equiv), ethylenediamine (4 equiv), and Pd(dppf)Cl_2_ (0.05 equiv) were dissolved in *n*‐PrOH (5.5 mL/mmol) in a microwave vial and purged with argon for 10 min. The reaction mixture was heated by microwave irradiation at 125 °C for 15 min (for **21** **a**) and 2×15 min (for **21** **b**). Then, the reaction mixture was filtrated through Celite® and purified with automated flash chromatography with *n*‐hexane/EtOAc (1:0→0:1) as eluent.

***N*** **‐(2‐Aminoethyl)‐7‐(3,5‐dimethylphenyl)‐2‐vinylpyrazolo[1,5‐*a*]pyrimidine‐5‐carboxamide (21** **a)**: Compound **15** **a** (0.051 g, 0.14 mmol) was used to give **21** **a** (0.023 g, 49 %) as a yellow solid. ^1^H NMR (400 MHz, CD_3_OD): *δ*=7.69 (s, 2H), 7.61 (s, 1H), 7.25 (s, 1H), 6.99 (s, 1H), 6.87 (dd, *J*=17.8, 11.1 Hz, 1H), 6.12 (dd, *J*=17.8, 1.3 Hz, 1H), 5.57 (dd, *J*=11.1, 1.3 Hz, 1H), 3.68 (t, *J*=5.9 Hz, 2H), 3.11 (t, *J*=5.9 Hz, 2H), 2.41 (s, 6H); ^13^C NMR (101 MHz, CD_3_OD): 166.4, 157.8, 150.6, 149.8, 148.9, 139.7, 134.0, 132.0, 130.3, 128.1, 120.2, 105.8, 95.5, 41.3, 39.9, 21.4; HRMS (ESI) *m/z*: [M+H]^+^ calcd for C_19_H_22_N_5_O 336.1824, found 336.1828.

***N*** **‐(2‐Aminoethyl)‐7‐cyclopropyl‐2‐vinylpyrazolo[1,5‐*a*]pyrimidine‐5‐carboxamide (21** **b)**: Compound **15** **b** (0.051 g, 0.16 mmol) was used to give **21** **b** (0.039 g, 87 %) as a pale brown solid. ^1^H NMR (400 MHz, CD_3_OD): *δ*=7.07 (s, 1H), 6.87 (s, 1H), 6.7 (dd, *J*=17.8, 11.0 Hz, 1H), 6.12 (dd, *J*=17.8, 1.3 Hz, 1H), 5.56 (dd, *J*=11.0, 1.3 Hz, 1H), 3.63 (t, *J*=6.00 Hz, 2H), 3.08 (t, *J*=6.00 Hz, 2H), 2.84 (tt, *J*=8.4, 5.2 Hz, 1H), 1.39–1.33 (m, 2H), 1.18–1.13 (m, 2H); ^13^C NMR (101 MHz, CD_3_OD): 166.3, 157.4, 154.6, 149.7, 149.3, 130.3, 120.0, 99.8, 95.2, 41.4, 40.3, 11.5, 10.3; HRMS (ESI) *m/z*: [M+H]^+^ calcd for C_14_H_18_N_5_O 272.1511, found 272.1513.

### Biological activities

All commercially obtained compounds had purities >90 % as specified by the vendor. Synthesized compounds were >95 % pure as determined by LC–MS and characterized by HRMS. The inhibition assay was carried out with purified TmPPase as previously reported.[^22^, ^23^, ^24^] The best hits **17** **a**, **19** **a**, and **20** **a**, were validated using eight compound concentrations in triplicate. Further hit validation was done against the purified mPPase from *P. falciparum* (PfPPase‐VP1) expressed in baculovirus‐infected insect cells (expression, purification and further enzyme analysis will be described in more detail elsewhere). 4 μL of the purified PfPPase‐VP1 (2.0 mg/mL) was reactivated in 96 μL of reactivation buffer, i. e. 20 mM 2‐(*N*‐morpholino)ethanesulfonic acid (MES) pH 6.5, 3.5 % (v/v) glycerol, 2 mM dithiothreitol (DTT) and 12 mg/mL l‐α‐phosphatidylcholine from soybean. For each tube strip, 3 μL of the reactivated PfPPase‐VP1 was added into 12 μL of reaction buffer (200 mM Tris‐Cl pH 8.0, 8.0 mM MgCl_2_, 333 mM KCl, 67 mM NaCl) and 25 μL of inhibitor solution. The reaction mixtures were incubated at 50 °C for 5 min and the assay was started with the addition of 10 μL of 2 mM sodium pyrophosphate and further incubation at 50 °C for 45 min. The reaction termination and colour development were done as earlier described.[^22^, ^23^, ^24^]

*Plasmodium falciparum* parasite strain 3D7 was used for testing the antiplasmodial activity of the test compounds. The parasite was maintained in culture as described previously.^[37]^ Briefly, parasites were cultured in O+ human erythrocytes at 5 % haematocrit in RPMI‐1640 medium supplemented with 0.5 % Albumax II (Gibco, Carlsbad, CA, USA), 200 μM hypoxanthine (Sigma, St. Louis, MO, USA) and 20 μg/mL gentamycin (Gibco). Parasites were synchronized with the sorbitol method as described.^[38]^ Test compounds were dissolved in DMSO to make a stock solution of 10 mM or 50 mM. Two‐fold serial dilutions of the test compounds were made in the culture medium to cover either the range of 100 μM–100 nM or 400 μM–390 nM. 50 μL of each dilution were mixed in 96‐well plate with 150 μL of 2 % haematocrit of 0.5 % parasitemia (ring stage). Compound dilutions were also mixed with 150 μL of 2 % haematocrit of uninfected erythrocytes to serve as baselines for the activity of the coloured compounds. DMSO concentration in the highest compound concentration was 0.8 %. After 72 h incubation at 37 °C, the parasite growth was quantified using fluorescent SYBR Green I®‐based assay as described.^[39]^ The half‐maximal inhibitory concentration (IC_50_) of the test compounds was assessed using the non‐linear regression fit model in Prism 7 (GraphPad Software, San Diego, CA, USA). The potent anti‐malarial drug, artemisinin, was used in parallel as a positive control. Each concentration of the test compounds was tested in duplicate and the assay was repeated three times. The mean and standard deviation of the three repeats were used to calculate the IC_50_.

## Conflict of interest

The authors declare no conflict of interest.

## Supporting information

As a service to our authors and readers, this journal provides supporting information supplied by the authors. Such materials are peer reviewed and may be re‐organized for online delivery, but are not copy‐edited or typeset. Technical support issues arising from supporting information (other than missing files) should be addressed to the authors.

Supporting Information
