# Supplementary material for: Exploration of Pyrazolo[1,5‐a]pyrimidines as Membrane‐Bound Pyrophosphatase Inhibitors
Source: ChemMedChem. 2021 Oct 12;16(21):3360–7. doi: 10.1002/cmdc.202100392 (PMC8597055; doi:10.1002/cmdc.202100392)
Supplement: Supplementary file 1 — Supporting Information [file CMDC-16-3360-s001.pdf]

ChemMedChem

Supporting Information

Exploration of Pyrazolo[1,5-*a*]pyrimidines as Membrane-Bound Pyrophosphatase Inhibitors

Niklas G. Johansson, Loïc Dreano, Keni Vidilaseris, Ayman Khattab, Jianing Liu, Arthur Lasbleiz, Orquidea Ribeiro, Alexandros Kiriazis, Gustav Boije af Gennäs, Seppo Meri, Adrian Goldman, Jari Yli-Kauhaluoma, and Henri Xhaard*

Table of Contents

1 List of screened compounds against TmPPase S3

1.1 5-Arylisoxazoles (Table S1) S3

1.2 Ethyl 5-(4-methoxy-3-sulfamoylphenyl)isoxazole-3-carboxylates (Table S2) S4

1.3 Sulfonamides (Table S3) S6

1.4 Amides (Table S4) S9

1.5 Pyrazolo[1,5-*a*]pyrimidines (Table S5) S13

2 Pharmacophore models for 2, 12 and 17a (Figure S1 and S2) S15

3 Aggregation data for 17a, 19a and 20a (Figure S3) S16

4 PfPPase inhibition assay for 12, 17a, 19a and 20a (Figure S4 and S5) S17

5 *P. falciparum* survival assay for 12 (Figure S6) S18

6 IC_{50} plots from the TmPPase inhibition assay S19

7 NMR spectra (for 15a–20a and 15b–20b) S30

8 Mass spectra (for 17a, 19a and 20a) S46

References S49

1 List of screened compounds against TmPPase

The blue and black structures indicate the compounds already presented in the main article and compounds only shown in the Supporting Information, respectively. Purities of the tested compounds were above 95% for the synthesized compounds and above 90% for the purchased ones as guaranteed by the vendor (Ambinter). IC_{50}, half maximal inhibitory concentration; $CI_{95\%}$, half maximal inhibitory concentration expressed as a 95% confidence interval.

1.1 5-Arylisoxazoles (Table S1)

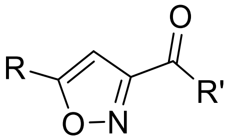

compd	R	R’	MW (Da)	IC_{50} [$CI_{95\%}$] (µM)	Chemical Formula
**SI-1**	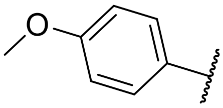	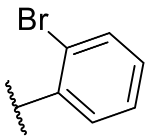	374	74 [68–81]	$C_{17}H_{12}BrNO_4$
**SI-2**	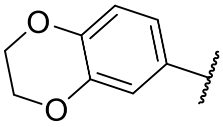	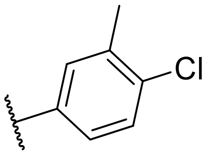	371	Inactive	$C_{19}H_{14}ClNO_5$
**SI-3**	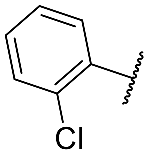	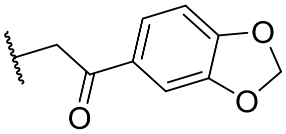	385	Inactive	$C_{19}H_{12}ClNO_6$
**SI-4**	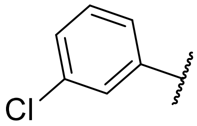	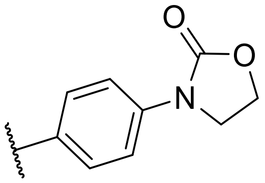	384	Inactive	$C_{19}H_{13}ClN_2O_5$

1.2 Ethyl 5-(4-methoxy-3-sulfamoylphenyl)isoxazole-3-carboxylates (Table S2)

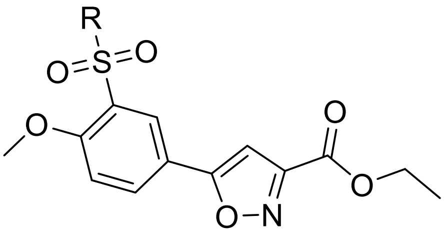

compd	R	MW (Da)	IC_{50} [$CI_{95\%}$] (μM)	Chemical Formula
**6**	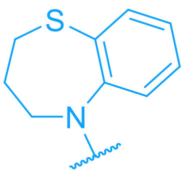	474	5.4 [5.1–5.7]	$C_{22}H_{22}N_2O_6S_2$
**7**	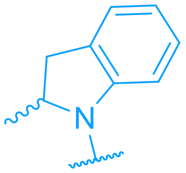	442	56 [53–58]	$C_{22}H_{22}N_2O_6S$
**8**	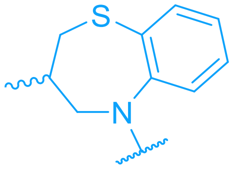	489	Inactive	$C_{23}H_{24}N_2O_6S_2$
**9**	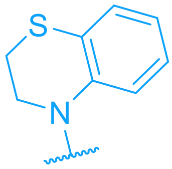	460	Inactive	$C_{21}H_{20}N_2O_6S_2$
**10**	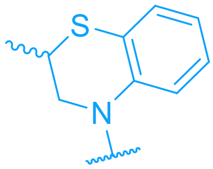	474	Inactive	$C_{22}H_{22}N_2O_6S_2$
**SI-5**	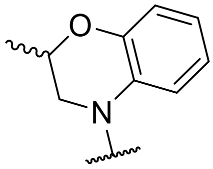	458	Inactive	$C_{22}H_{22}N_2O_7S$
**SI-6**	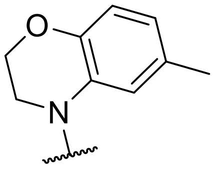	458	Slightly active	$C_{22}H_{22}N_2O_7S$

**SI-7**	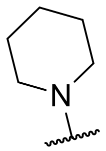	394	Inactive	$C_{18}H_{22}N_2O_6S$
**SI-8**	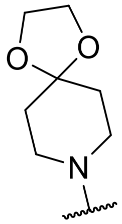	452	Inactive	$C_{20}H_{24}N_2O_8S$
**SI-9**	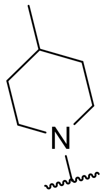	408	Inactive	$C_{19}H_{24}N_2O_6S$
**SI-10**	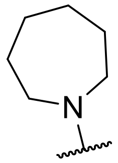	408	Slightly active	$C_{19}H_{24}N_2O_6S$

1.3 Sulfonamides (Table S3)

compd	R	MW (Da)	IC_{50} [$CI_{95\%}$] (μM)	Chemical Formula
**11**	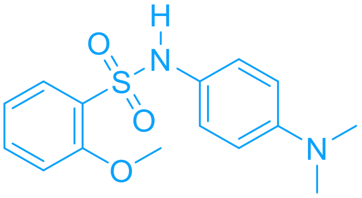	306	14 [4.1–64]	$C_{15}H_{18}N_2O_3S$
**SI-11**	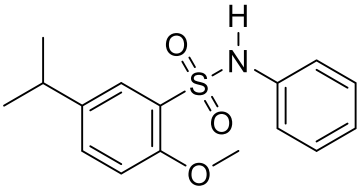	305	Inactive	$C_{16}H_{19}NO_3S$
**SI-12**	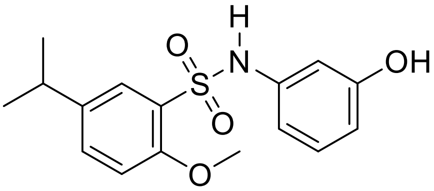	321	Inactive	$C_{16}H_{19}NO_4S$
**SI-13**	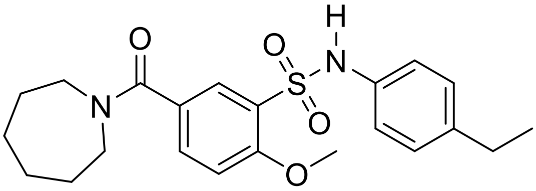	416	Inactive	$C_{22}H_{28}N_2O_4S$
**SI-14**	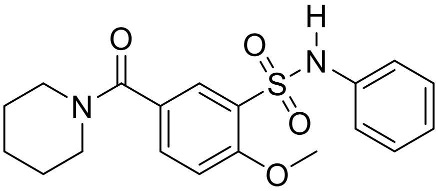	374	Inactive	$C_{19}H_{22}N_2O_4S$
**SI-15**	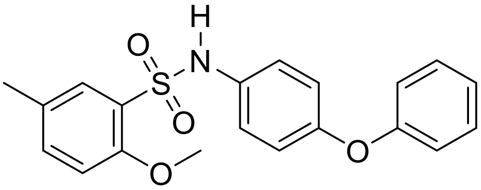	369	Inactive	$C_{20}H_{19}NO_4S$
**SI-16**	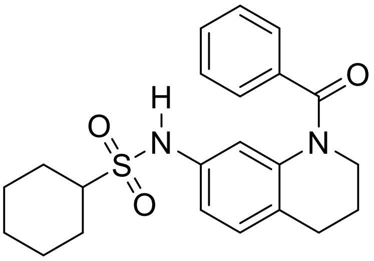	398	Inactive	$C_{22}H_{26}N_2O_3S$

**SI-17**	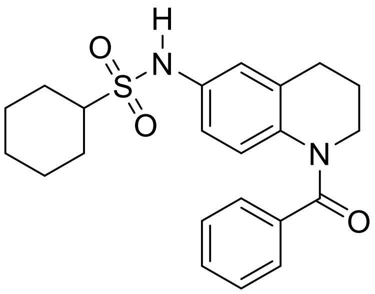	398	54 [49–60]	$C_{22}H_{26}N_2O_3S$
**SI-18**	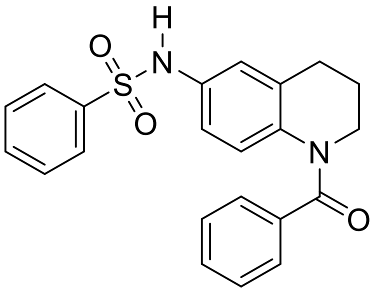	392	Inactive	$C_{22}H_{20}N_2O_3S$
**SI-19**	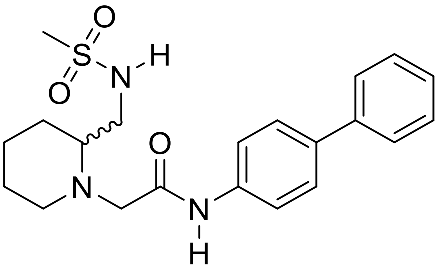	401	55 [50–60]	$C_{21}H_{27}N_3O_3S$
**SI-20**	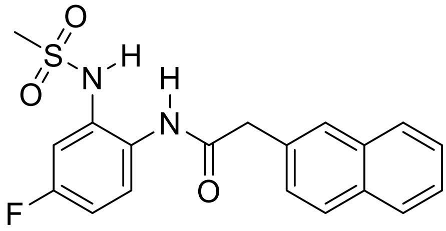	372	Inactive	$C_{19}H_{17}FN_2O_3S$
**SI-21**	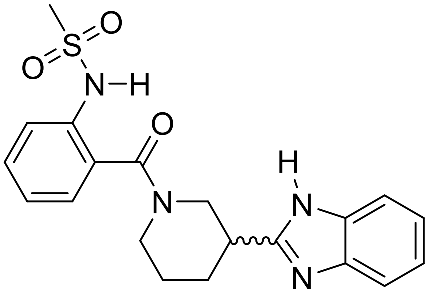	398	Inactive	$C_{20}H_{22}N_4O_3S$
**SI-22**	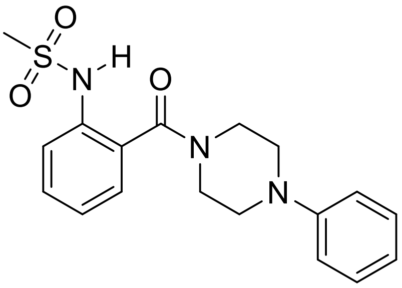	359	220 [130–380]	$C_{18}H_{21}N_3O_3S$

**SI-23**	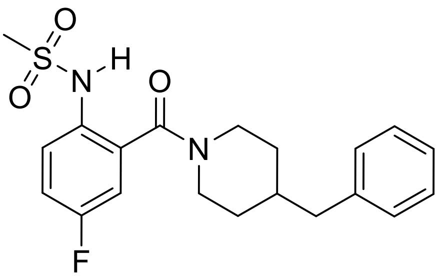	390	Inactive	$C_{20}H_{23}FN_2O_3S$
**SI-24**	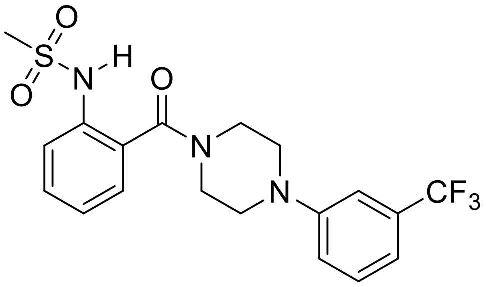	427	Inactive	$C_{19}H_{20}F_3N_3O_3S$
**SI-25**	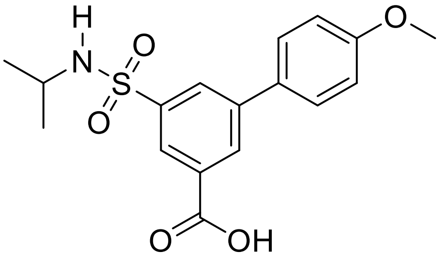	349	Inactive	$C_{17}H_{19}NO_5S$
**SI-26**	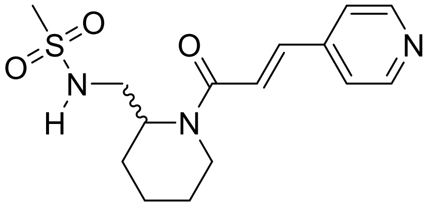	323	Inactive	$C_{15}H_{21}N_3O_3S$
**SI-27**	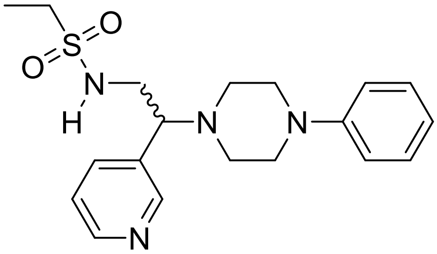	374	Inactive	$C_{19}H_{26}N_4O_2S$

1.4 Amides (Table S4)

compd	R	MW (Da)	IC_{50} $[CI_{95\%}]$ (μM)	Chemical Formula
**12**	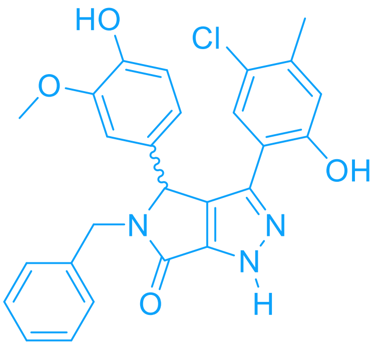	475	25 [22–30]	$C_{26}H_{22}ClN_3O_4$
**SI-28**	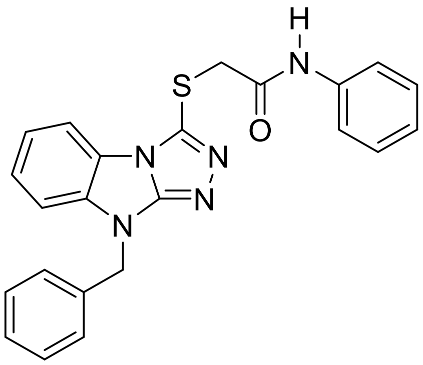	413	Inactive	$C_{23}H_{19}N_5OS$
**SI-29**	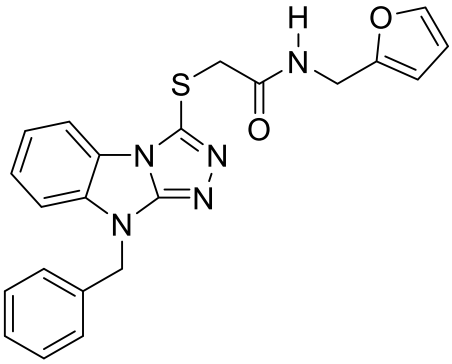	417	Inactive	$C_{22}H_{19}N_5O_2S$
**SI-30**	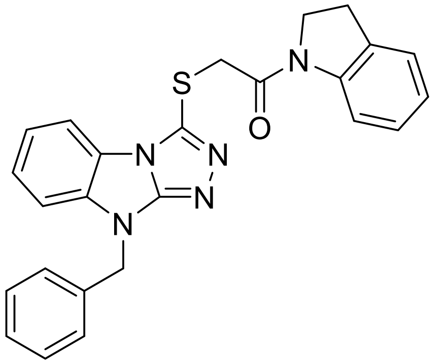	439	Inactive	$C_{25}H_{21}N_5OS$

**SI-31**	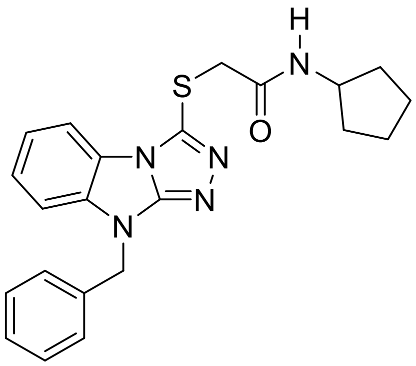	405	Inactive	$C_{22}H_{23}N_5OS$
**SI-32**	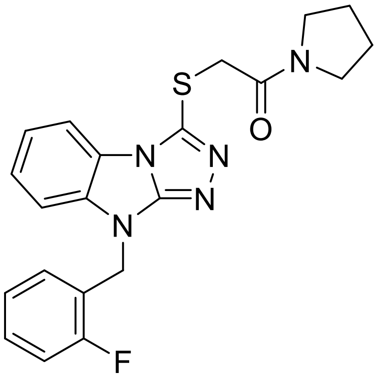	409	Inactive	$C_{21}H_{20}FN_5OS$
**SI-33**	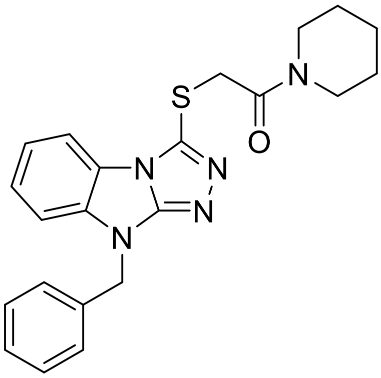	405	Inactive	$C_{22}H_{23}N_5OS$
**SI-34**	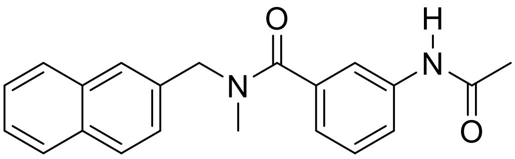	332	Inactive	$C_{21}H_{20}N_2O_2$
**SI-35**	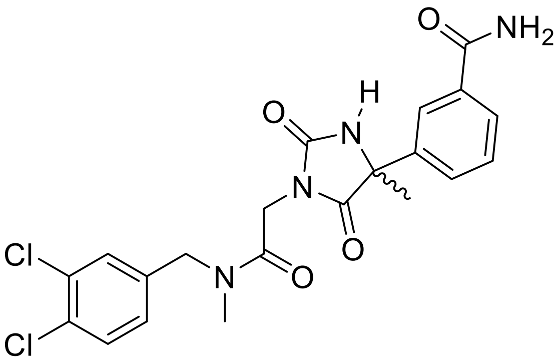	463	73 [71–75]	$C_{21}H_{20}Cl_2N_4O_4$

**SI-36**	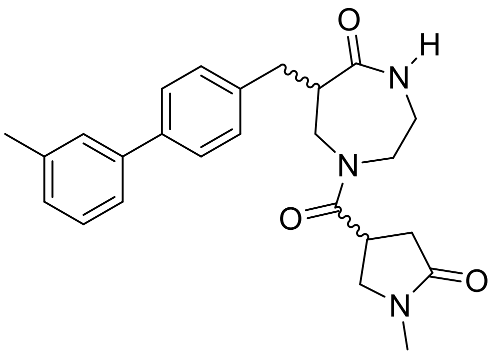	419	Inactive	$C_{25}H_{29}N_3O_3$
**SI-37**	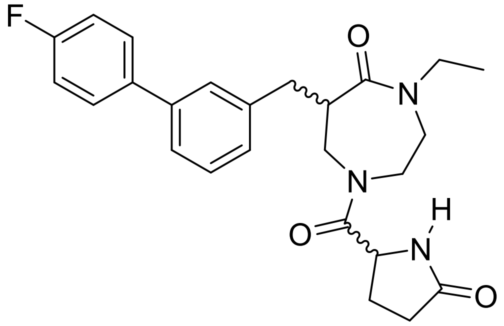	437	Inactive	$C_{25}H_{28}FN_3O_3$
**SI-38**	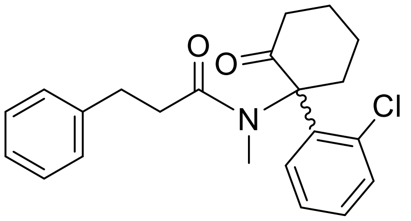	369	Inactive	$C_{22}H_{24}ClNO_2$
**SI-39**	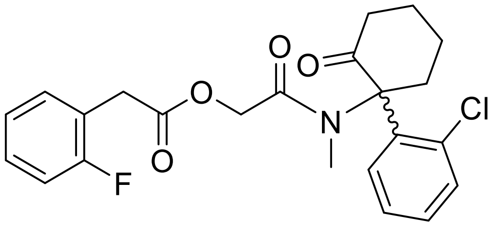	431	Inactive	$C_{23}H_{23}ClFNO_4$
**SI-40**	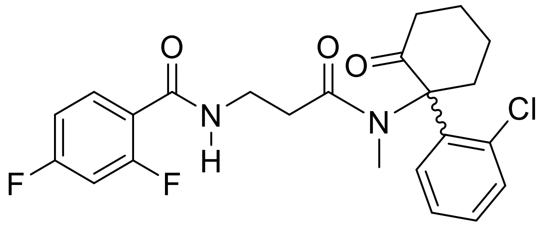	448	43 [37–48]	$C_{23}H_{23}ClF_2N_2O_3$
**SI-41**	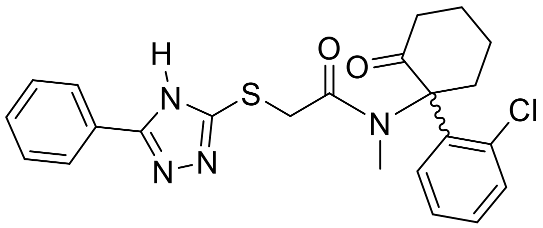	454	150 [96–360]	$C_{23}H_{23}ClN_4O_2S$

**SI-42**	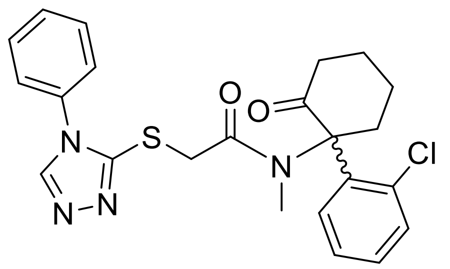	454	Inactive	$C_{23}H_{23}ClN_4O_2S$
**SI-43**	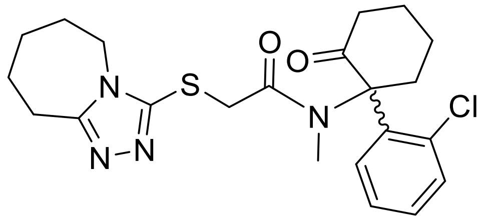	483	Inactive	$C_{22}H_{28}Cl_2N_4O_2S$
**SI-44**	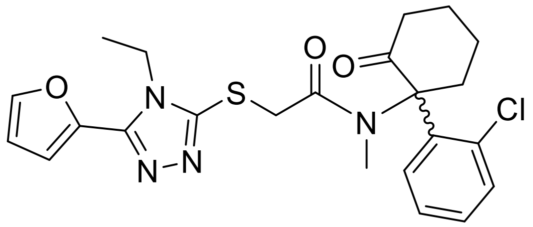	472	Inactive	$C_{23}H_{25}ClN_4O_3S$
**SI-45**	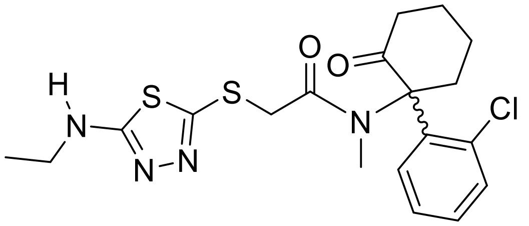	438	Inactive	$C_{19}H_{23}ClN_4O_2S_2$

1.5 Pyrazolo[1,5-*a*]pyrimidines (Table S5)

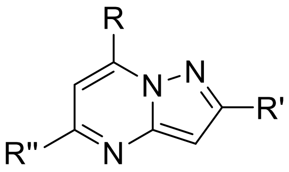

compd	R	R’	R’’´	MW (Da)	IC_{50} [$CI_{95\%}$] (µM)	Chemical Formula
**15a**	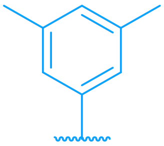	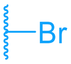	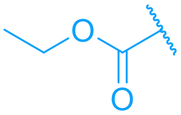	374	120 [110–140]	$C_{17}H_{16}BrN_3O_2$
**15b**	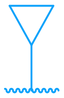	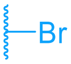	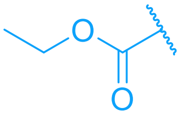	310	150 [130–180]	$C_{12}H_{12}BrN_3O_2$
**16a**	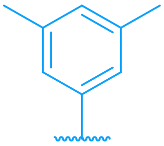	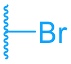	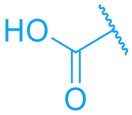	346	Inactive	$C_{15}H_{12}BrN_3O_2$
**16b**	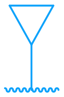	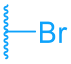	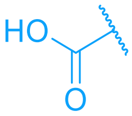	282	Inactive	$C_{10}H_8BrN_3O_2$
**17a**	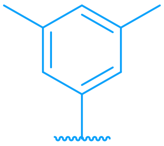	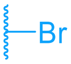	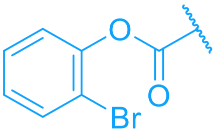	501	14 [13–15]	$C_{21}H_{15}Br_2N_3O_2$
**17b**	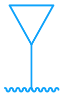	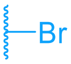	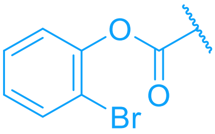	437	Inactive	$C_{16}H_{11}Br_2N_3O_2$
**18a**	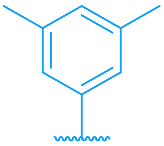	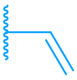	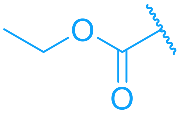	321	54 [51–58]	$C_{19}H_{19}N_3O_2$
**18b**	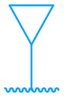	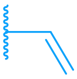	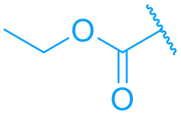	257	Inactive	$C_{14}H_{15}N_3O_2$
**19a**	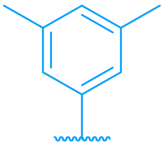	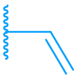	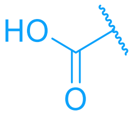	293	14 [13–15]	$C_{17}H_{15}N_3O_2$

**19b**	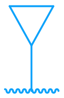	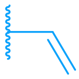	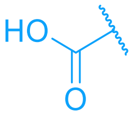	229	Inactive	$C_{12}H_{11}N_3O_2$
**20a**	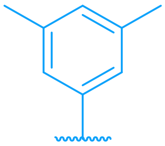	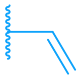	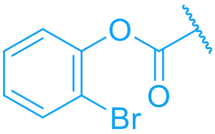	448	18 [17–19]	$C_{23}H_{18}BrN_3O_2$
**20b**	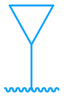	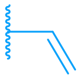	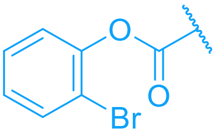	384	Inactive	$C_{18}H_{14}BrN_3O_2$
**21a**	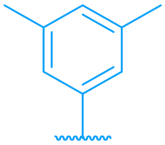	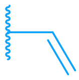	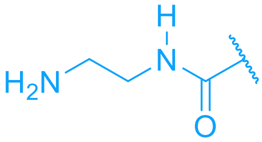	335	72 [61–85]	$C_{19}H_{21}N_5O$
**21b**	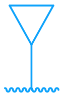	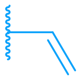	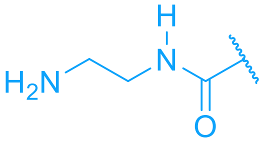	271	260 [190–360]	$C_{14}H_{17}N_5O$

2 Pharmacophore models for 2, 12 and 17a (Figure S1 and S2)

The chemical similarity of compounds **2**, **12**, and **17a** was studied using pharmacophore models with the Schrödinger Maestro suite (version 2019-4).[1] Compounds **2** and **17a**, as well as **12** and **17a**, were compared independently (Figure S1 and S2). The "Develop Pharmacophore model" tool was used with the following options: compounds aligned using the **multiple ligands** method; **Find best alignment and common features;** and the option **Generate conformers** turned on. As a result, several alignments (60 for each pair) are returned, classified according to the number of common features and their overlap. The best alignment is shown below for **12** and **17a,** as well as **2** and **17a**. Overall, there is a significant overlap of compounds (Figure S1-C and S2-C) and of pharmacophoric features (Figure S1-D and S2-D), which support the change of scaffold that was operated.

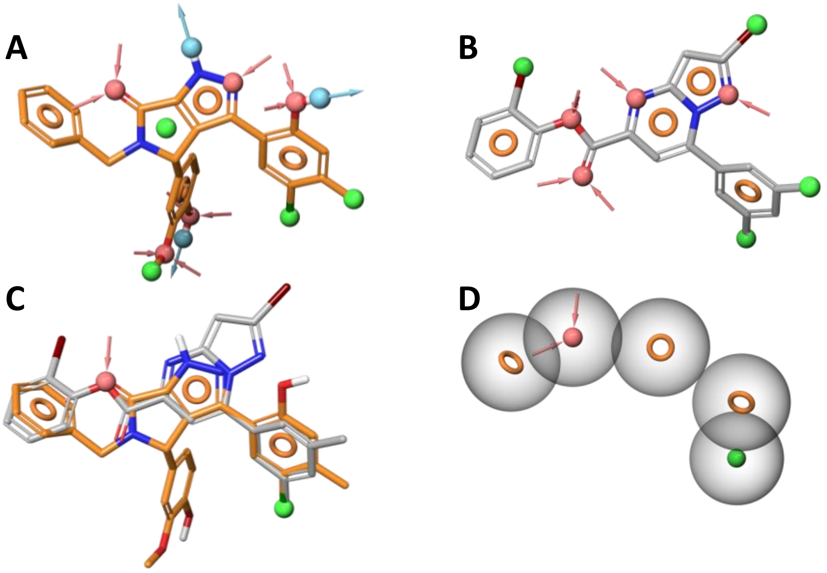

Figure S1. A and B – Pharmacophoric features (Aromatic, orange ring; Hydrophobic, green sphere; Hydrogen bond Acceptor, red arrow, Hydrogen bond Donor, blue arrow) mapped on (A) compound **12**, orange and (B) compound **17a**, grey; C – Alignment of compound **12** and **17a** and common features; D – Pharmacophore features shared by compounds **12** and **17a**.

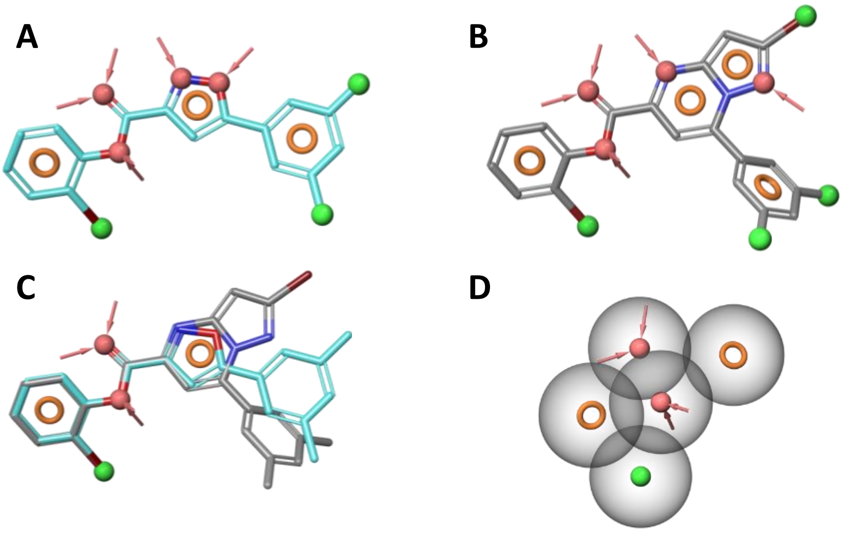

Figure S2. A and B – Pharmacophoric features (Aromatic, orange ring; Hydrophobic, green sphere; Hydrogen bond Acceptor, red arrow, Hydrogen bond Donor, blue arrow) mapped on (A) compound **2**, cyan and (B) compound **17a**, grey; C – Alignment of compound **2** and **17a** and common features; D– Pharmacophore features shared by compounds **2** and **17a**.

3 Aggregation data for 17a, 19a and 20a (Figure S3)

Potential colloidal aggregation in the TmPPase model system was studied by nephelometric methods using Nepheloskan Ascent® (Labsystems, Finland). The original assay conditions were simulated, and the light scattering of potential aggregates in the mixture was studied at six concentrations (100 µM, 50 µM, 20 µM, 10 µM, 1 µM and 0.1 µM). Aggregation of the blank and compounds **17a**, **19a** and **20a** was measured as quadruplicates from one independent experiment at three different voltages (300 V, 400 V and 500 V) at room temperature.

Compounds **17a** and **20a** showed aggregate formation at 100 µM, 50 µM and 20 µM. The obtained values at 10 µM, 1 µM and 0.1 µM were similar to those observed by the blank, indicating merely negligible/no detectable aggregation. For compound **19a** there was no detectable aggregation.

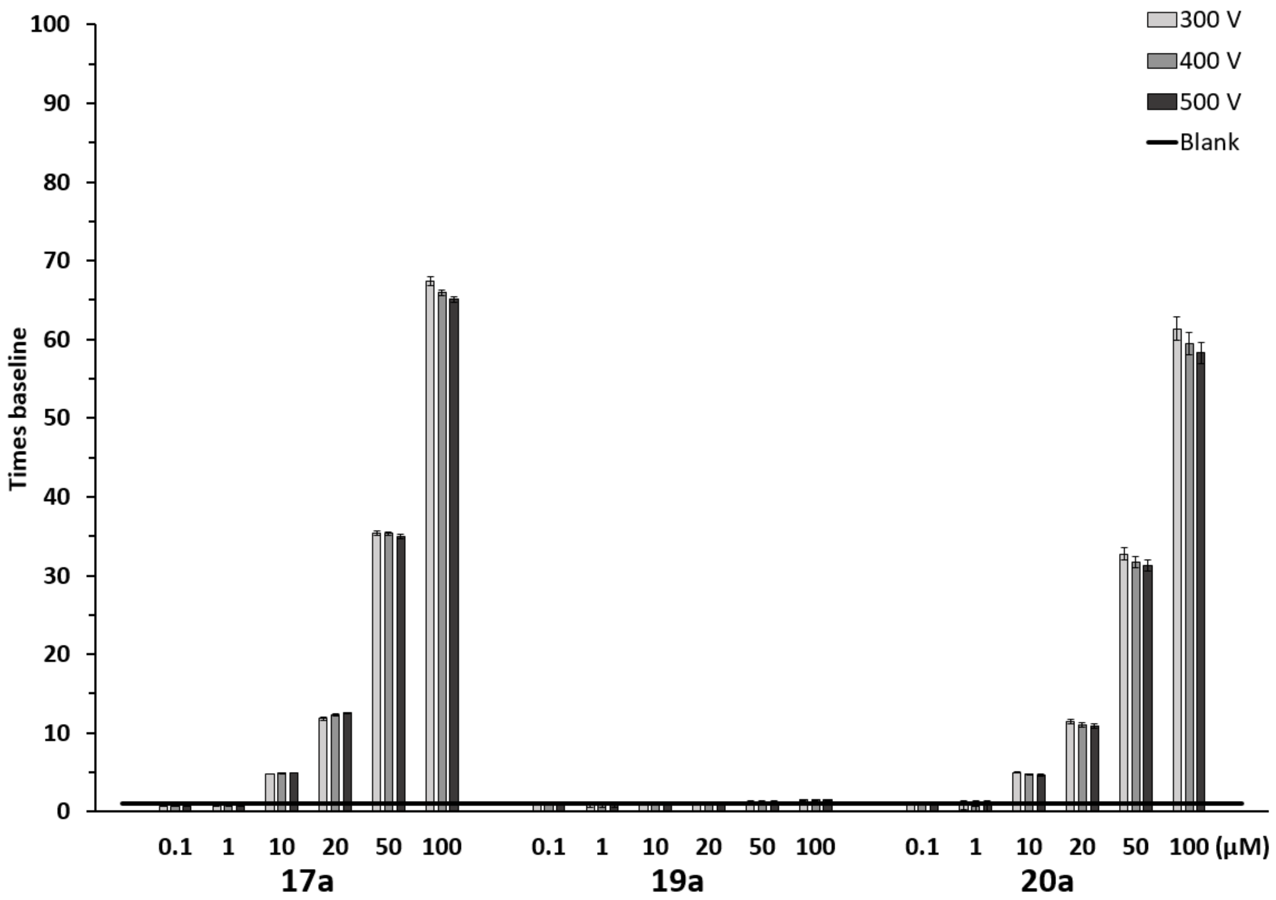

Figure S3. Aggregation data for compounds **17a**, **19a** and **20a**. The data, measured in relative nephelometric units (RNU), is shown as the mean ± SD and normalized to the blank.

4 PfPPase inhibition assay for 12, 17a, 19a and 20a (Figure S4 and S5)

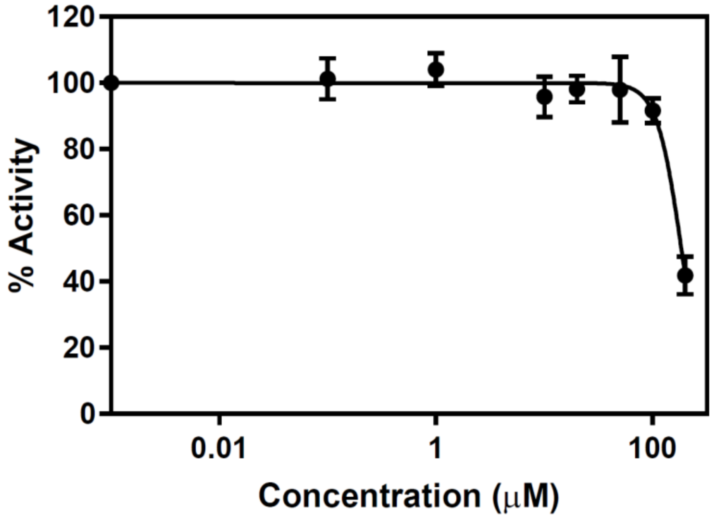

Figure S4. Inhibition of *P. falciparum* mPPase (PfPPaseVP-1) by **12** (IC_{50} = 180 µM. All data are shown as mean ± SD with three replicates.

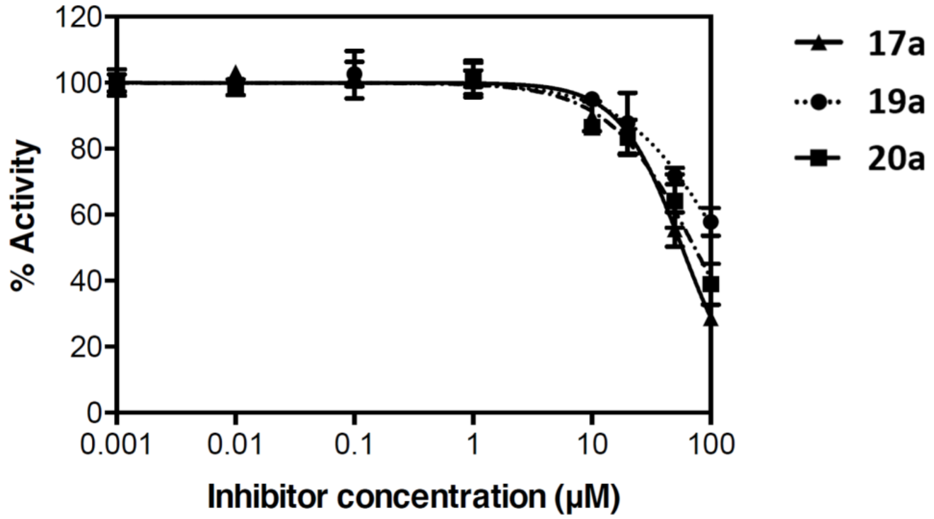

Figure S5. Inhibition of *P. falciparum* mPPase (PfPPaseVP-1) by **17a** (IC_{50} = 58 µM), **19a** (IC_{50} = 130 µM) and **20a** (IC_{50} = 74 µM). All data are shown as mean ± SD with three replicates.

5 *P. falciparum* survival assay for 12 (Figure S6)

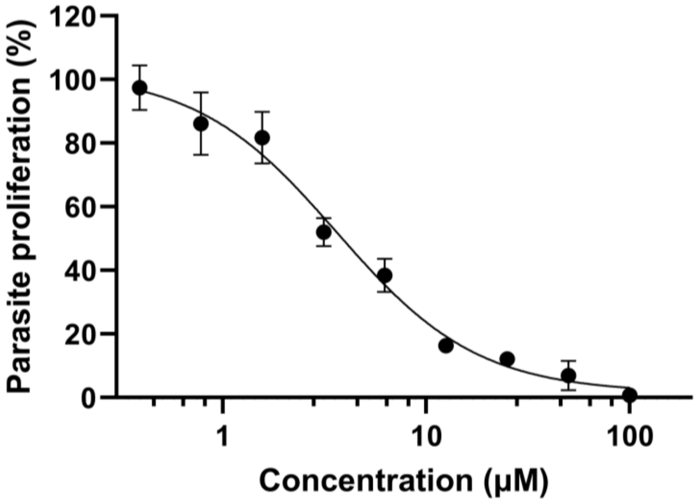

Figure S6. Effect of **12** (IC_{50} = 3.6 μM) on *P. falciparum* growth. All data are shown as mean ± SD with three replicates.

6 IC_{50} plots from the TmPPase inhibition assay

The blue and black curves refer to compounds presented already in the main article and only shown in the Supporting Information, respectively. IC_{50}, half maximal inhibitory concentration; $CI_{95\%}$, half maximal inhibitory concentration expressed as a 95% confidence interval (given in square brackets); NA, IC_{50} not assigned.

IC_{50} determinations were done using R 3.6.1[2] and the n-parameter logistic regression (nplr) package[3] version 0.1.7. In this package, the weighted nplr, given *x* (compound concentrations) and *y* values (activity) is calculated using Richard's equation (eq 1) where *B* and *T* are the bottom and top asymptotes, respectively; *b* and *xmid* are the Hill slope and the x coordinate at inflexion point, respectively; and *s* is an asymmetric coefficient. The model parameters were simultaneously optimized using non-linear minimization, using a sum of squared errors.[4] The standard error of the model, defined as the squared error on the fitted values, is used to estimate a confidence interval at 95% on the predicted IC_{50} values.

$$y = B + \frac{T - B}{(1 + 10^{b(xmid-x)})^{s}} \quad (1)$$

6.1. 5-Arylisoxazoles

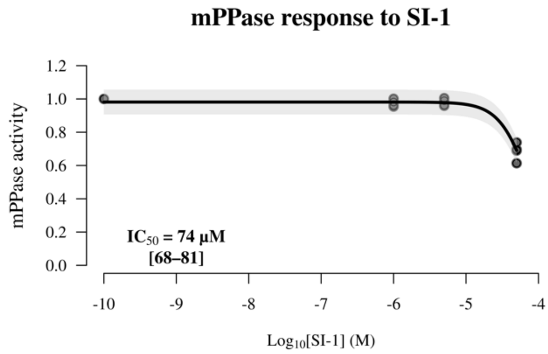

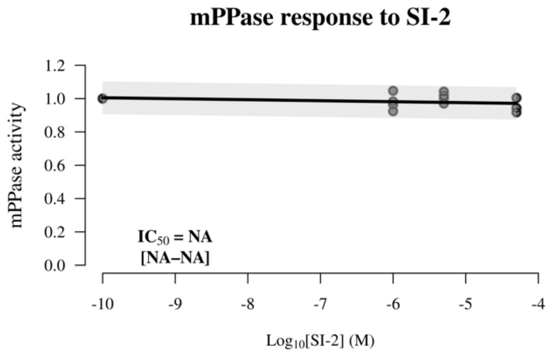

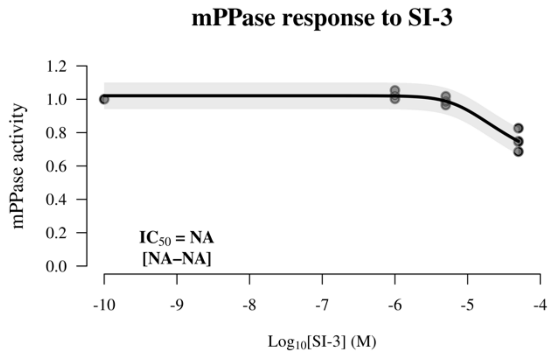

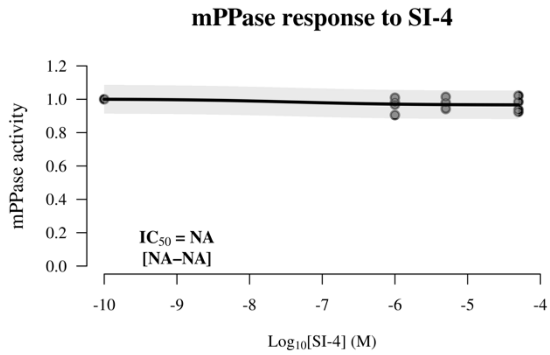

6.2. Ethyl 5-(4-methoxy-3-sulfamoylphenyl)isoxazole-3-carboxylates

mPPase response to 6

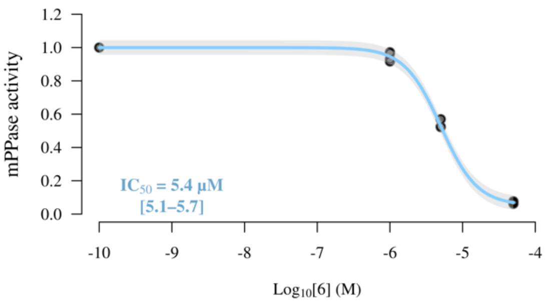

mPPase response to 7

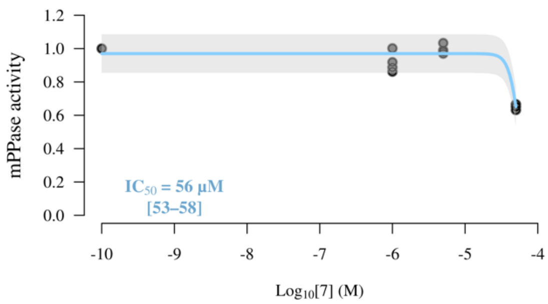

mPPase response to 8

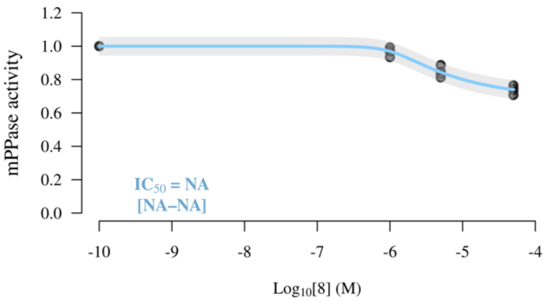

mPPase response to 9

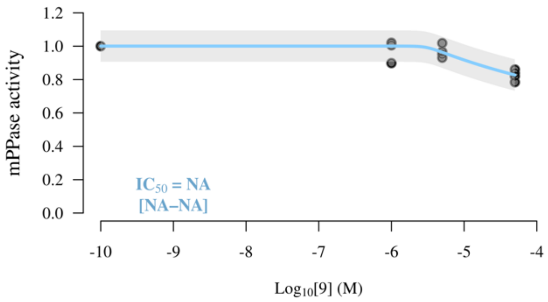

mPPase response to 10

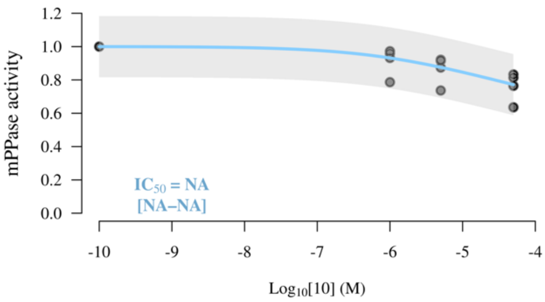

mPPase response to SI-5

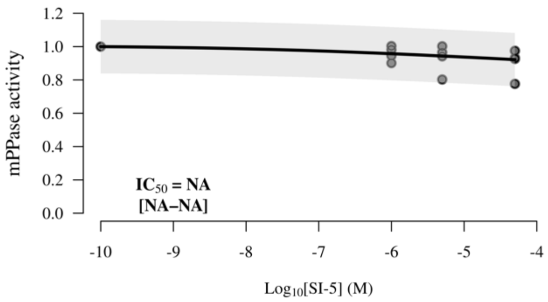

mPPase response to SI-6

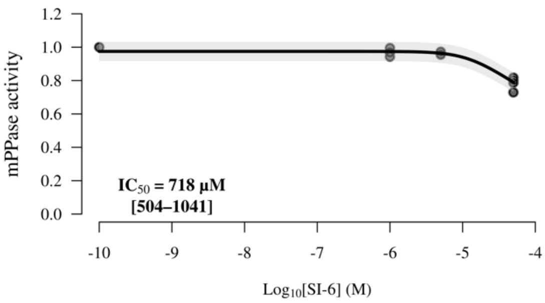

mPPase response to SI-7

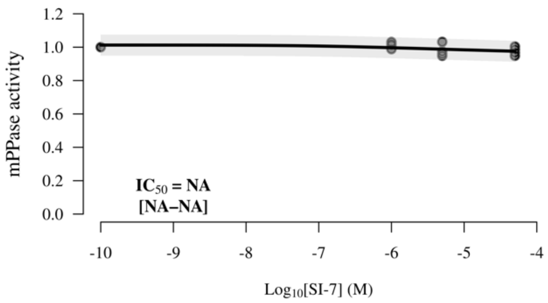

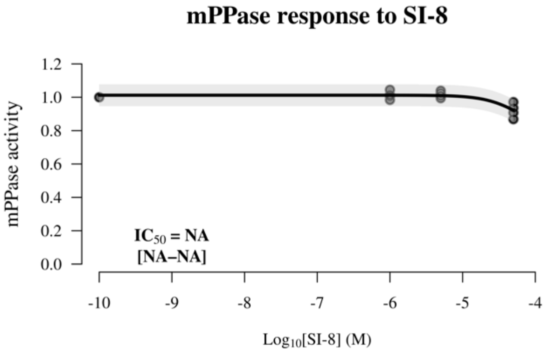
mPPase response to SI-8
mPPase activity
IC$_{50}$ = NA
[NA–NA]
Log$_{10}$[SI-8] (M)

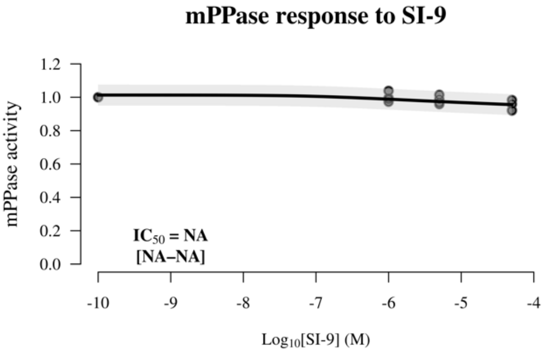
mPPase response to SI-9
mPPase activity
IC$_{50}$ = NA
[NA–NA]
Log$_{10}$[SI-9] (M)

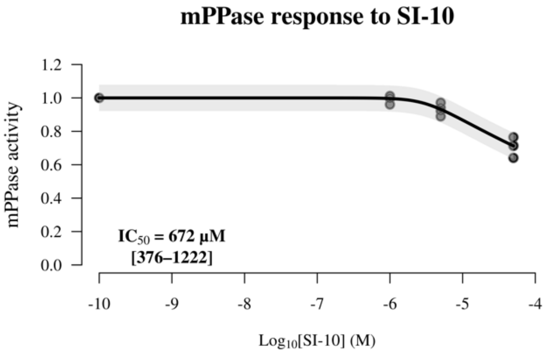
mPPase response to SI-10
mPPase activity
IC$_{50}$ = 672 µM
[376–1222]
Log$_{10}$[SI-10] (M)

6.3. Sulfonamides

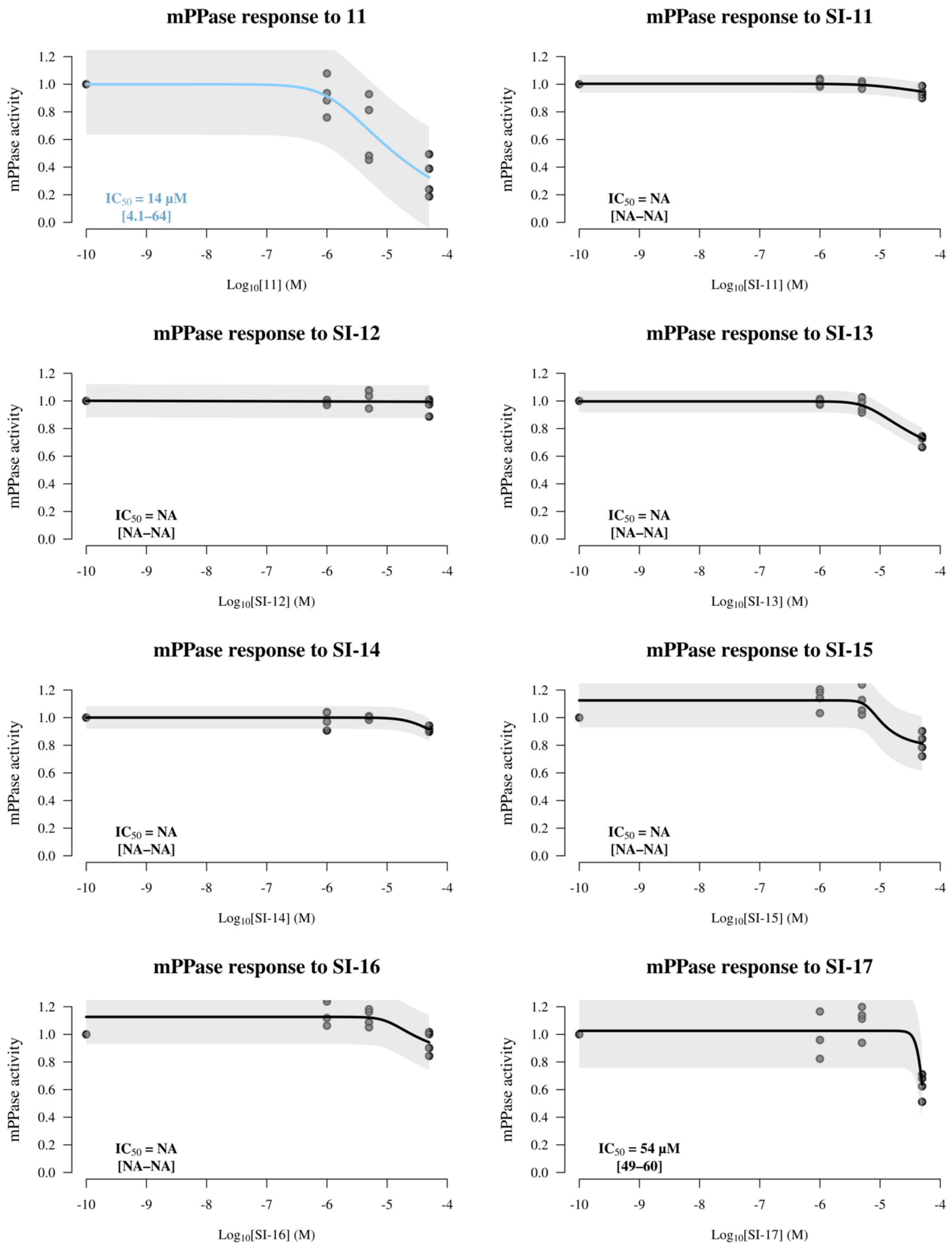

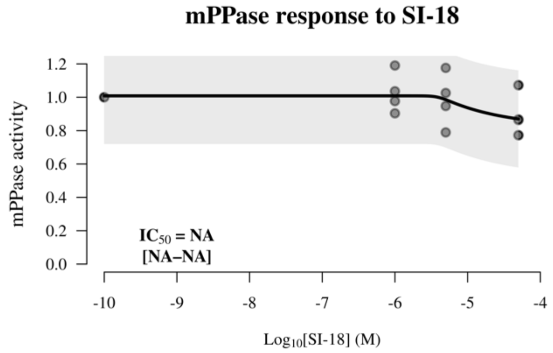
mPPase response to SI-18
mPPase activity
1.2
1.0
0.8
0.6
0.4
0.2
0.0
IC_{50} = NA
[NA–NA]
-10 -9 -8 -7 -6 -5 -4
Log_{10}[SI-18] (M)

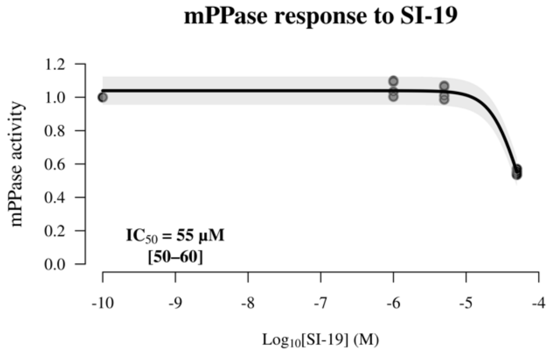
mPPase response to SI-19
mPPase activity
1.2
1.0
0.8
0.6
0.4
0.2
0.0
IC_{50} = 55 µM
[50–60]
-10 -9 -8 -7 -6 -5 -4
Log_{10}[SI-19] (M)

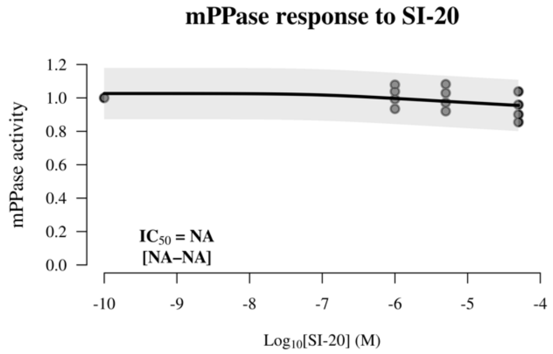
mPPase response to SI-20
mPPase activity
1.2
1.0
0.8
0.6
0.4
0.2
0.0
IC_{50} = NA
[NA–NA]
-10 -9 -8 -7 -6 -5 -4
Log_{10}[SI-20] (M)

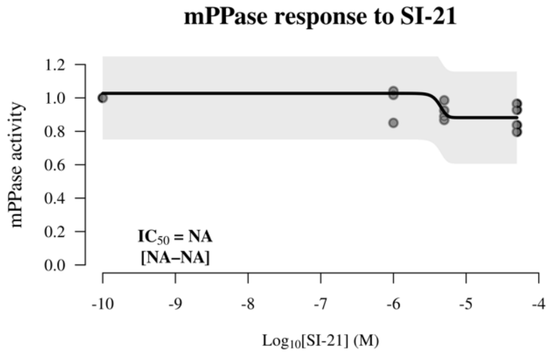
mPPase response to SI-21
mPPase activity
1.2
1.0
0.8
0.6
0.4
0.2
0.0
IC_{50} = NA
[NA–NA]
-10 -9 -8 -7 -6 -5 -4
Log_{10}[SI-21] (M)

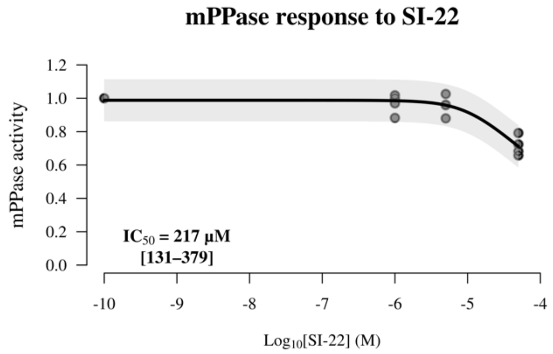
mPPase response to SI-22
mPPase activity
1.2
1.0
0.8
0.6
0.4
0.2
0.0
IC_{50} = 217 µM
[131–379]
-10 -9 -8 -7 -6 -5 -4
Log_{10}[SI-22] (M)

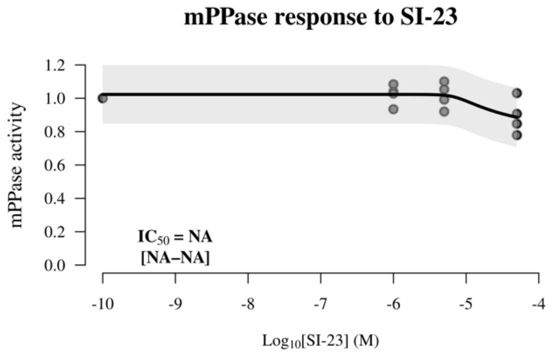
mPPase response to SI-23
mPPase activity
1.2
1.0
0.8
0.6
0.4
0.2
0.0
IC_{50} = NA
[NA–NA]
-10 -9 -8 -7 -6 -5 -4
Log_{10}[SI-23] (M)

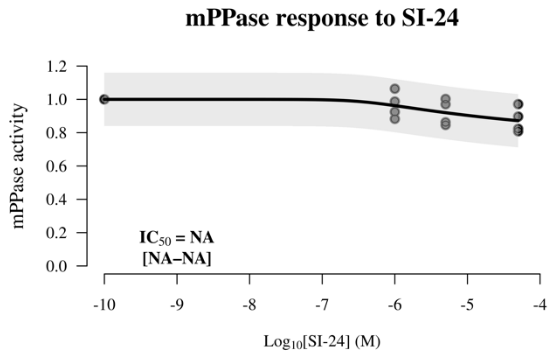
mPPase response to SI-24
mPPase activity
1.2
1.0
0.8
0.6
0.4
0.2
0.0
IC_{50} = NA
[NA–NA]
-10 -9 -8 -7 -6 -5 -4
Log_{10}[SI-24] (M)

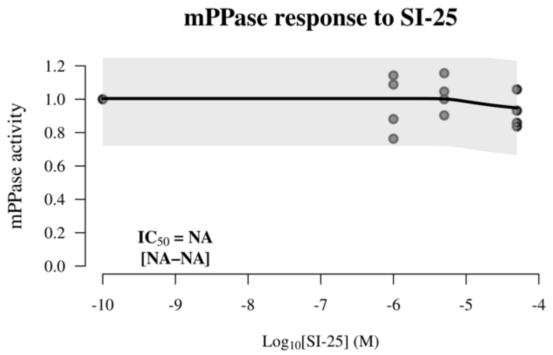
mPPase response to SI-25
mPPase activity
1.2
1.0
0.8
0.6
0.4
0.2
0.0
IC_{50} = NA
[NA–NA]
-10 -9 -8 -7 -6 -5 -4
Log_{10}[SI-25] (M)

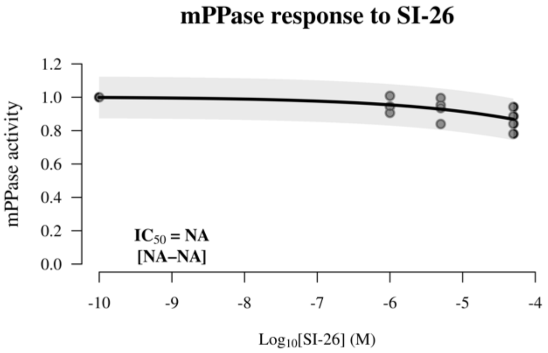
mPPase response to SI-26
mPPase activity
IC50 = NA
[NA–NA]
Log10[SI-26] (M)

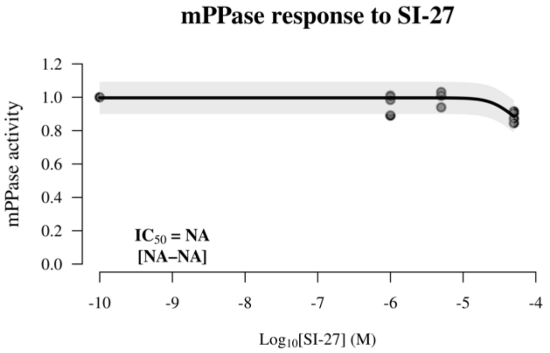
mPPase response to SI-27
mPPase activity
IC50 = NA
[NA–NA]
Log10[SI-27] (M)

6.4. Amides

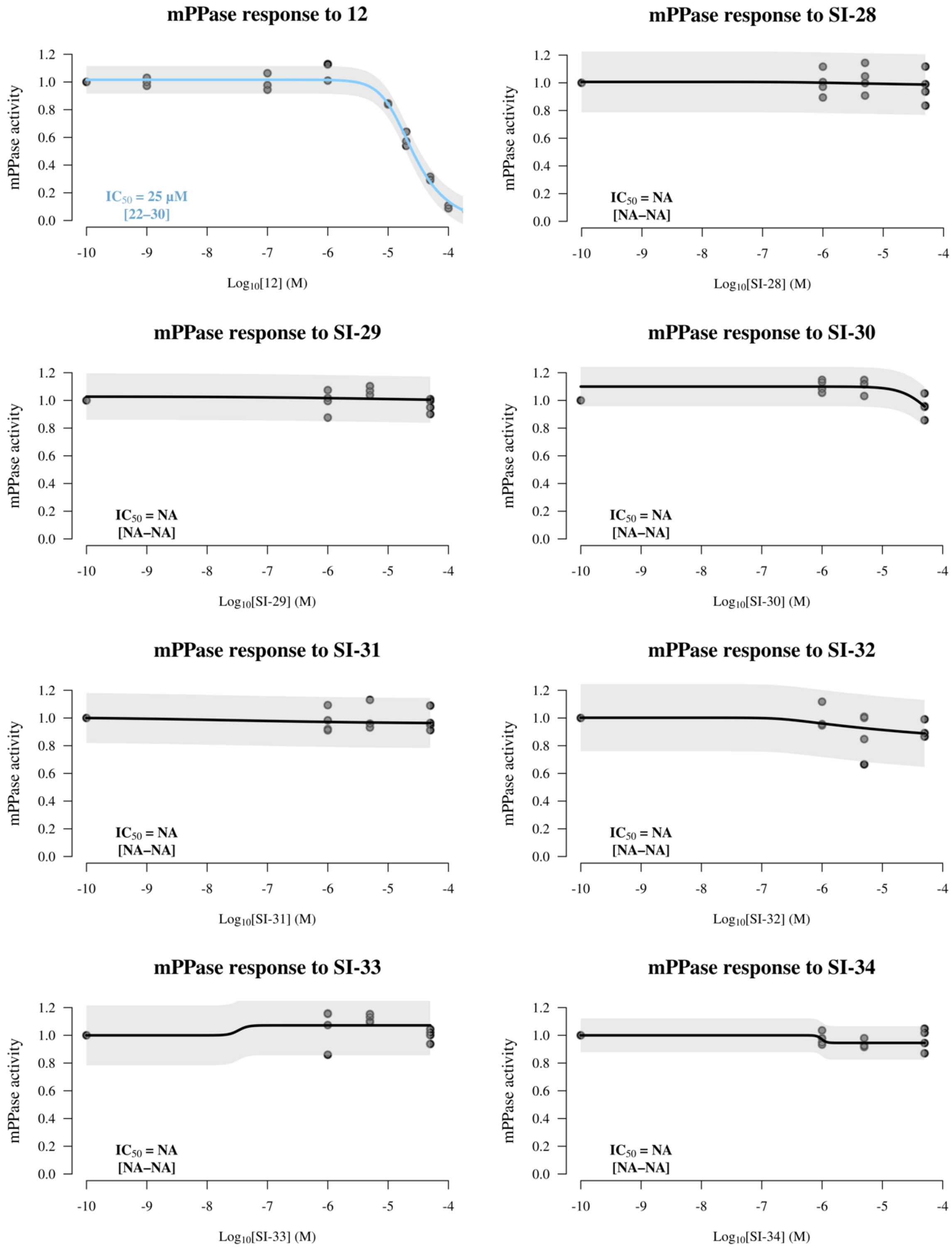
mPPase response to 12
mPPase activity
IC50 = 25 µM
[22–30]
Log10[12] (M)
mPPase response to SI-28
IC50 = NA
[NA–NA]
Log10[SI-28] (M)
mPPase response to SI-29
IC50 = NA
[NA–NA]
Log10[SI-29] (M)
mPPase response to SI-30
IC50 = NA
[NA–NA]
Log10[SI-30] (M)
mPPase response to SI-31
IC50 = NA
[NA–NA]
Log10[SI-31] (M)
mPPase response to SI-32
IC50 = NA
[NA–NA]
Log10[SI-32] (M)
mPPase response to SI-33
IC50 = NA
[NA–NA]
Log10[SI-33] (M)
mPPase response to SI-34
IC50 = NA
[NA–NA]
Log10[SI-34] (M)

mPPase response to SI-35

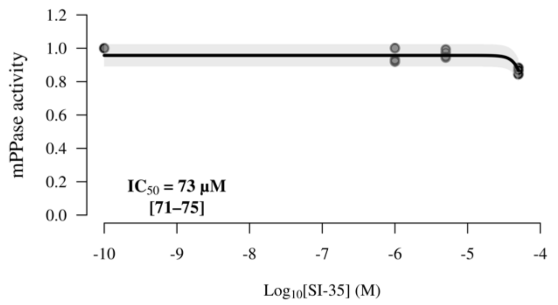

mPPase response to SI-36

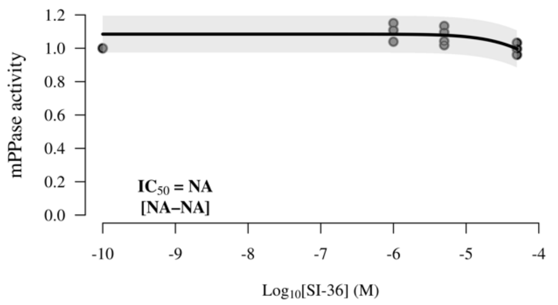

mPPase response to SI-37

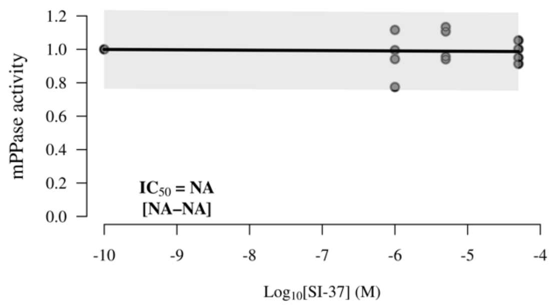

mPPase response to SI-38

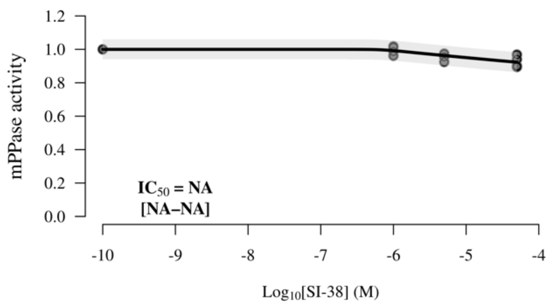

mPPase response to SI-39

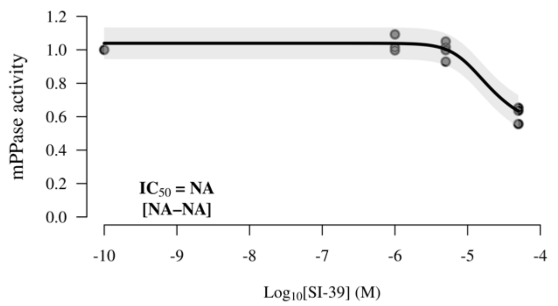

mPPase response to SI-40

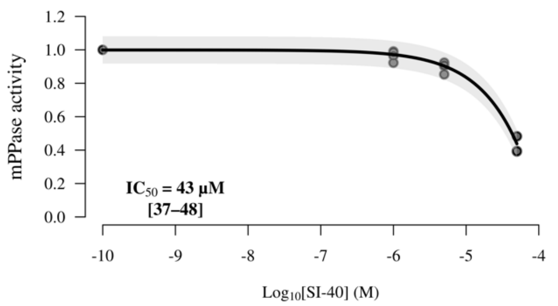

mPPase response to SI-41

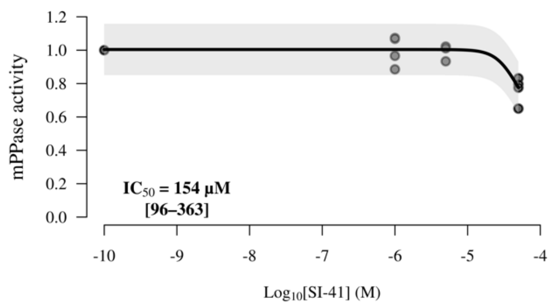

mPPase response to SI-42

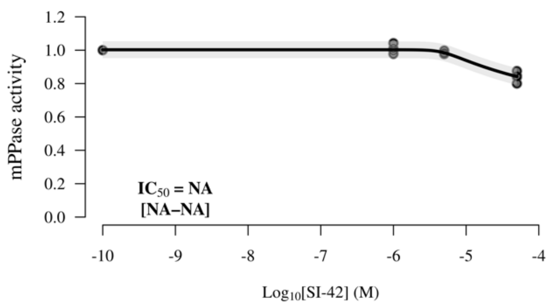

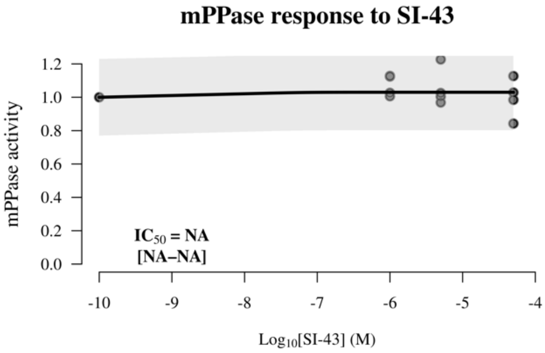
mPPase response to SI-43
mPPase activity
1.2
1.0
0.8
0.6
0.4
0.2
0.0
IC50 = NA
[NA–NA]
-10
-9
-8
-7
-6
-5
-4
Log10[SI-43] (M)

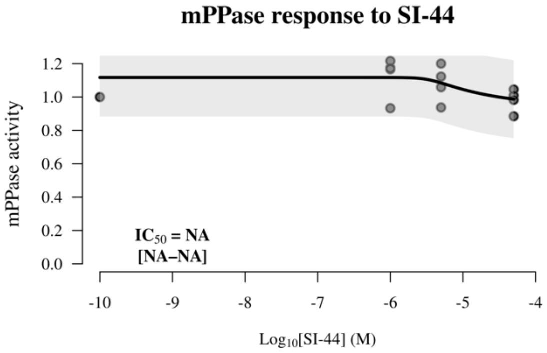
mPPase response to SI-44
mPPase activity
1.2
1.0
0.8
0.6
0.4
0.2
0.0
IC50 = NA
[NA–NA]
-10
-9
-8
-7
-6
-5
-4
Log10[SI-44] (M)

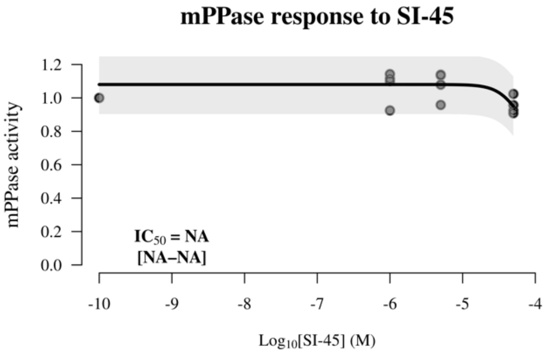
mPPase response to SI-45
mPPase activity
1.2
1.0
0.8
0.6
0.4
0.2
0.0
IC50 = NA
[NA–NA]
-10
-9
-8
-7
-6
-5
-4
Log10[SI-45] (M)

6.5. Pyrazolo[1,5-*a*]pyrimidines

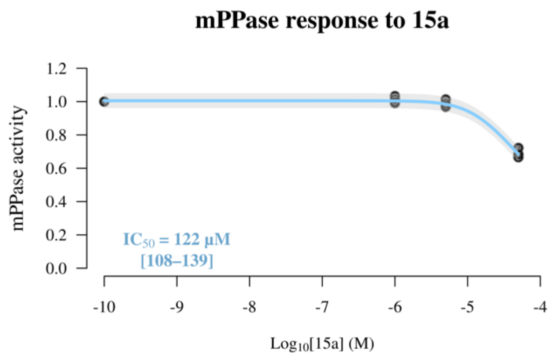

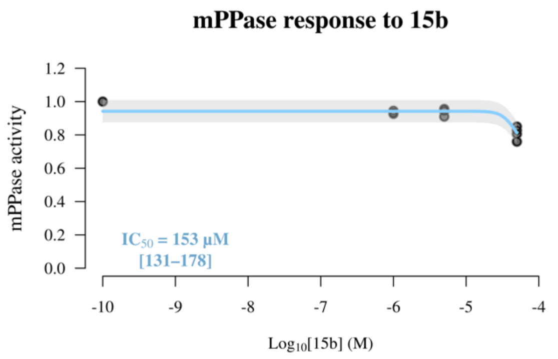

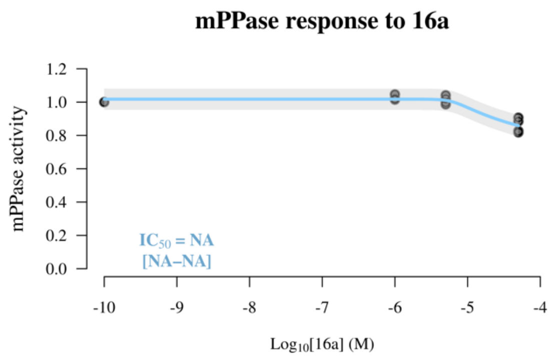

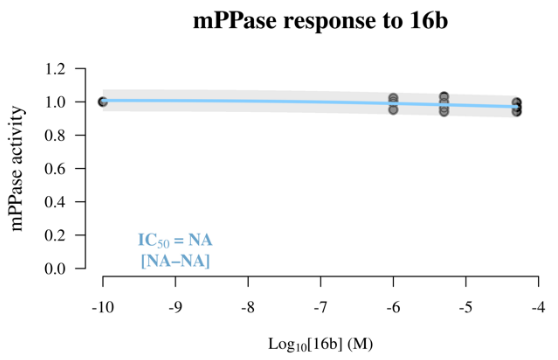

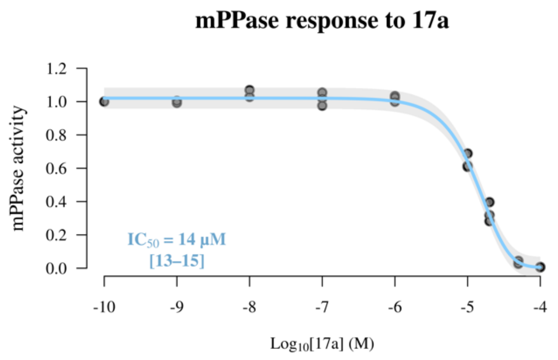

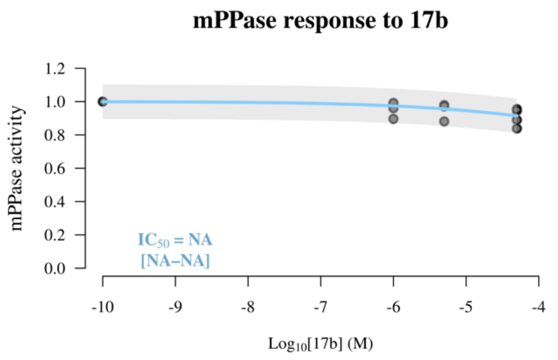

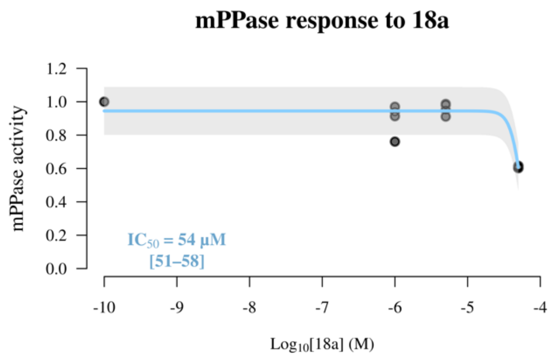

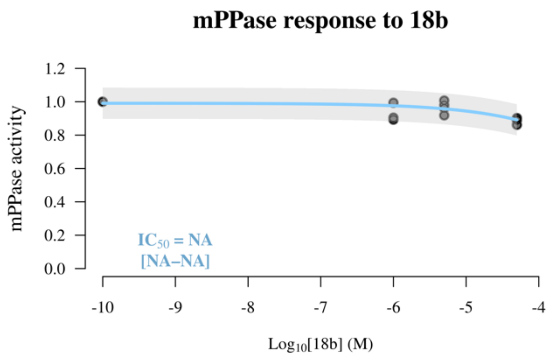

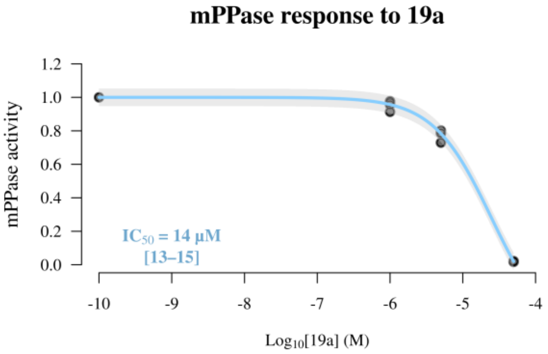
mPPase response to 19a
mPPase activity
IC50 = 14 µM
[13–15]
Log10[19a] (M)

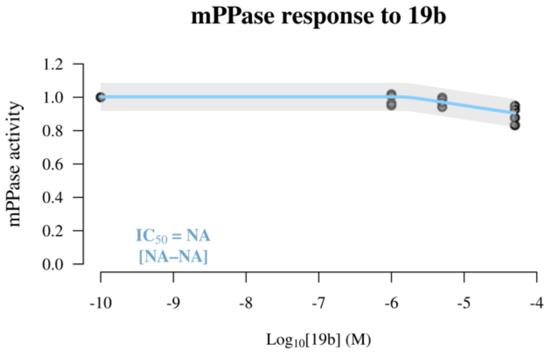
mPPase response to 19b
mPPase activity
IC50 = NA
[NA–NA]
Log10[19b] (M)

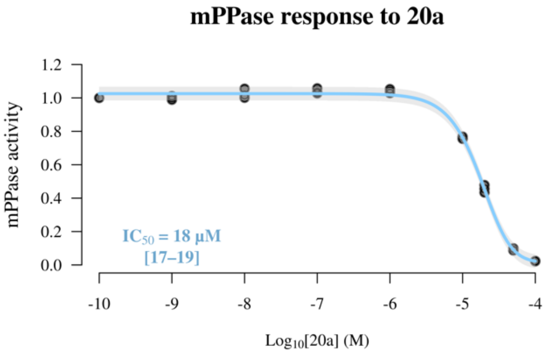
mPPase response to 20a
mPPase activity
IC50 = 18 µM
[17–19]
Log10[20a] (M)

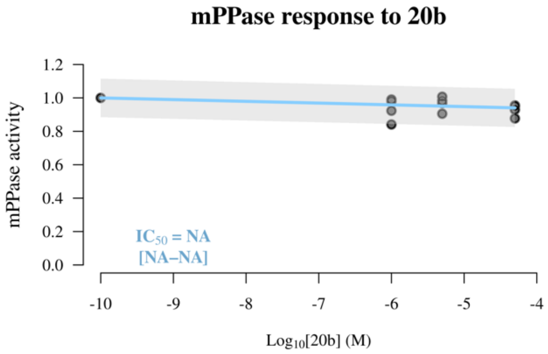
mPPase response to 20b
mPPase activity
IC50 = NA
[NA–NA]
Log10[20b] (M)

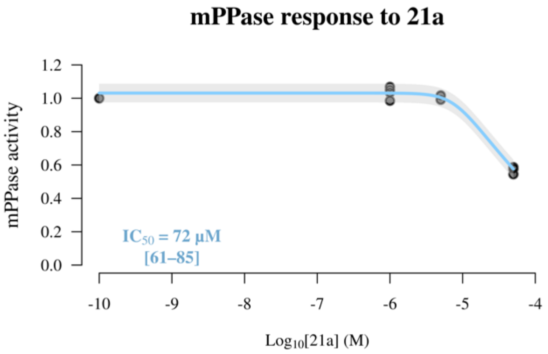
mPPase response to 21a
mPPase activity
IC50 = 72 µM
[61–85]
Log10[21a] (M)

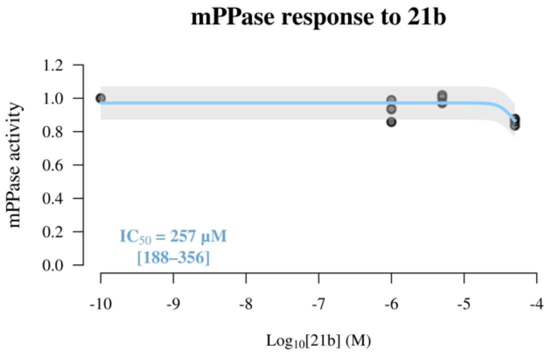
mPPase response to 21b
mPPase activity
IC50 = 257 µM
[188–356]
Log10[21b] (M)

7 NMR spectra (for 15a–20a and 15b–20b)

^{1}H NMR of compound **14a** in $CDCl_3$ (7.26 ppm)

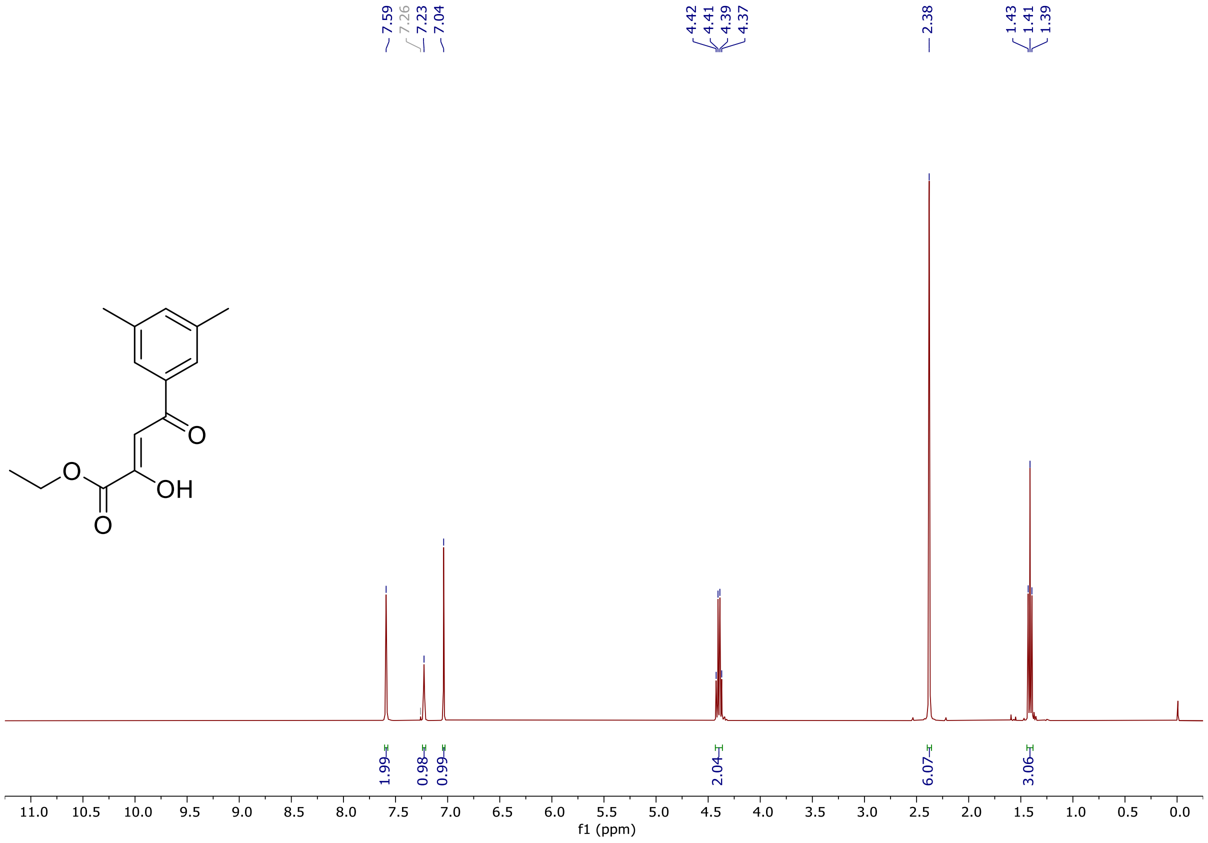

^{13}C NMR of compound **14a** in $CDCl_3$ (77.16 ppm)

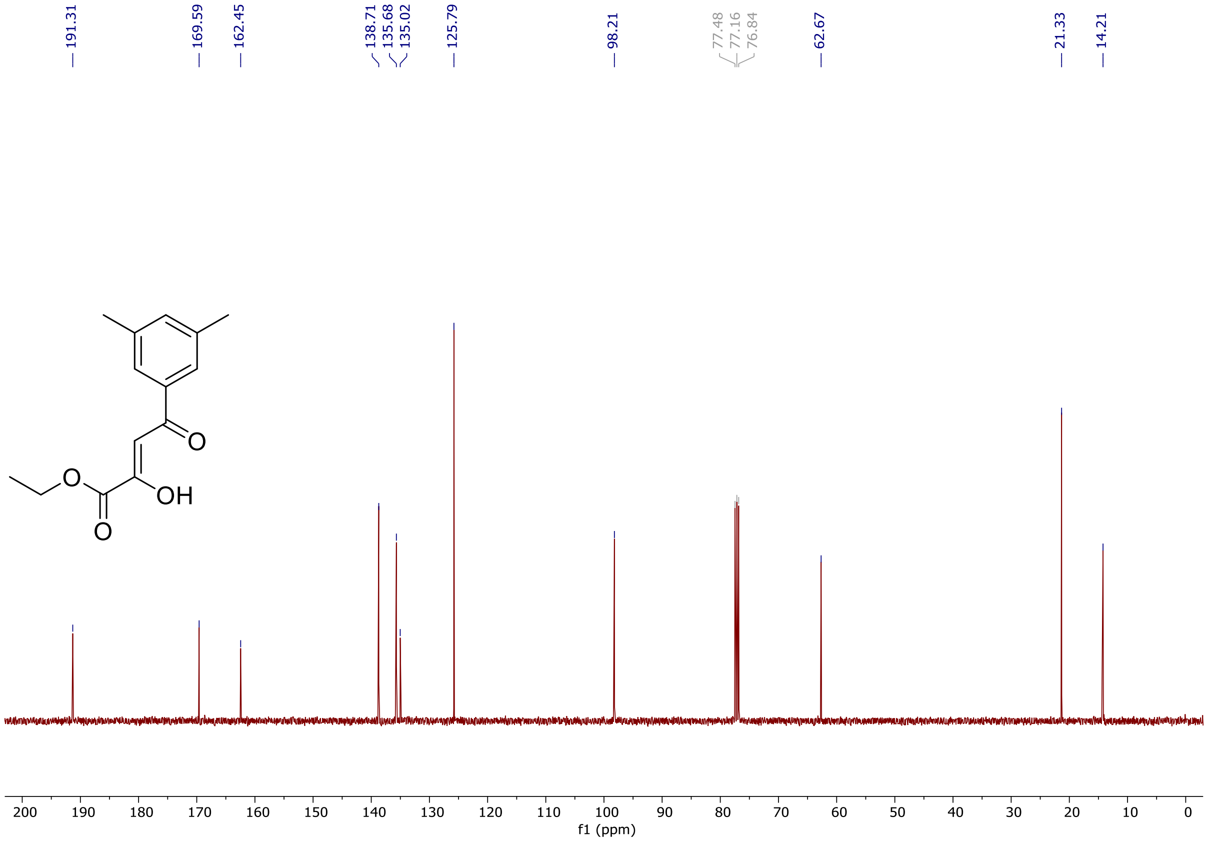

^{1}H NMR of compound **14b** in $CDCl_3$ (7.26 ppm)

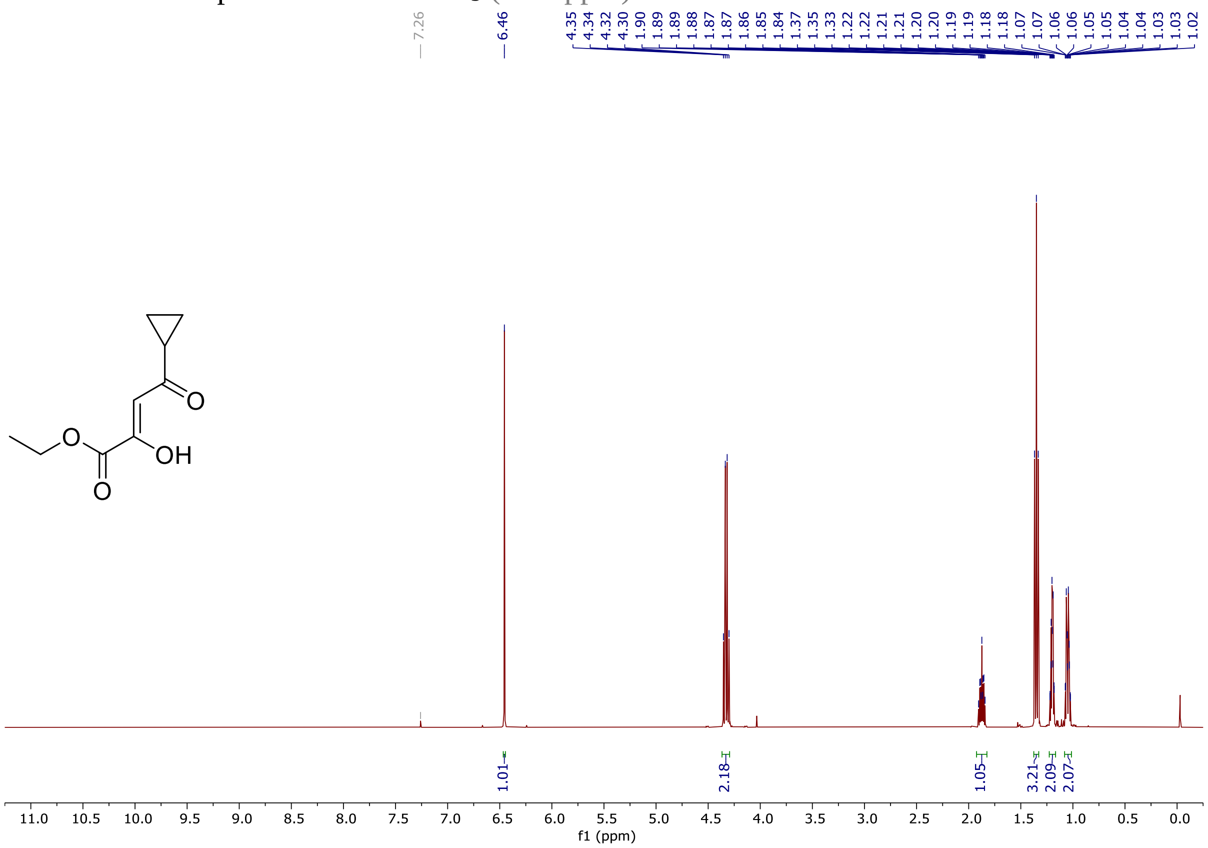

^{13}C NMR of compound **14b** in $CDCl_3$ (77.16 ppm)

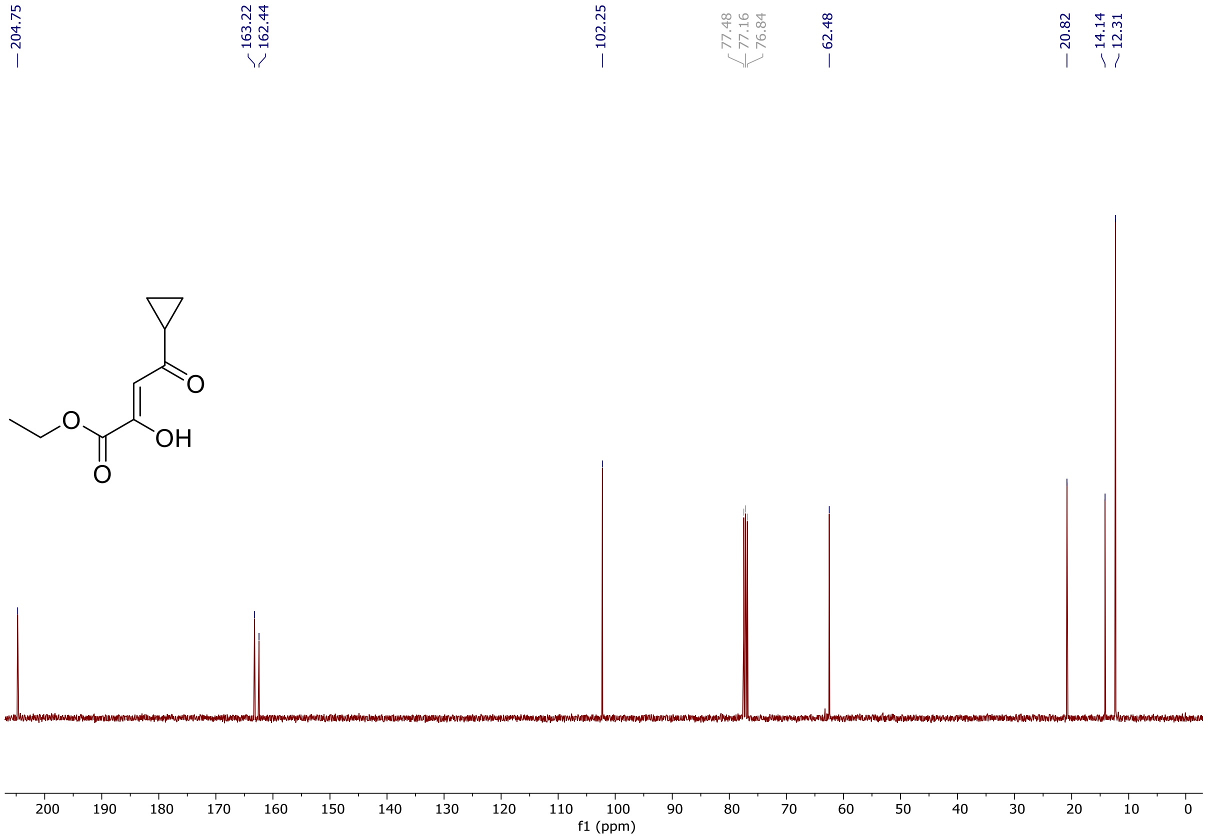

^{1}H NMR of compound **15a** in $CDCl_3$ (7.26 ppm)

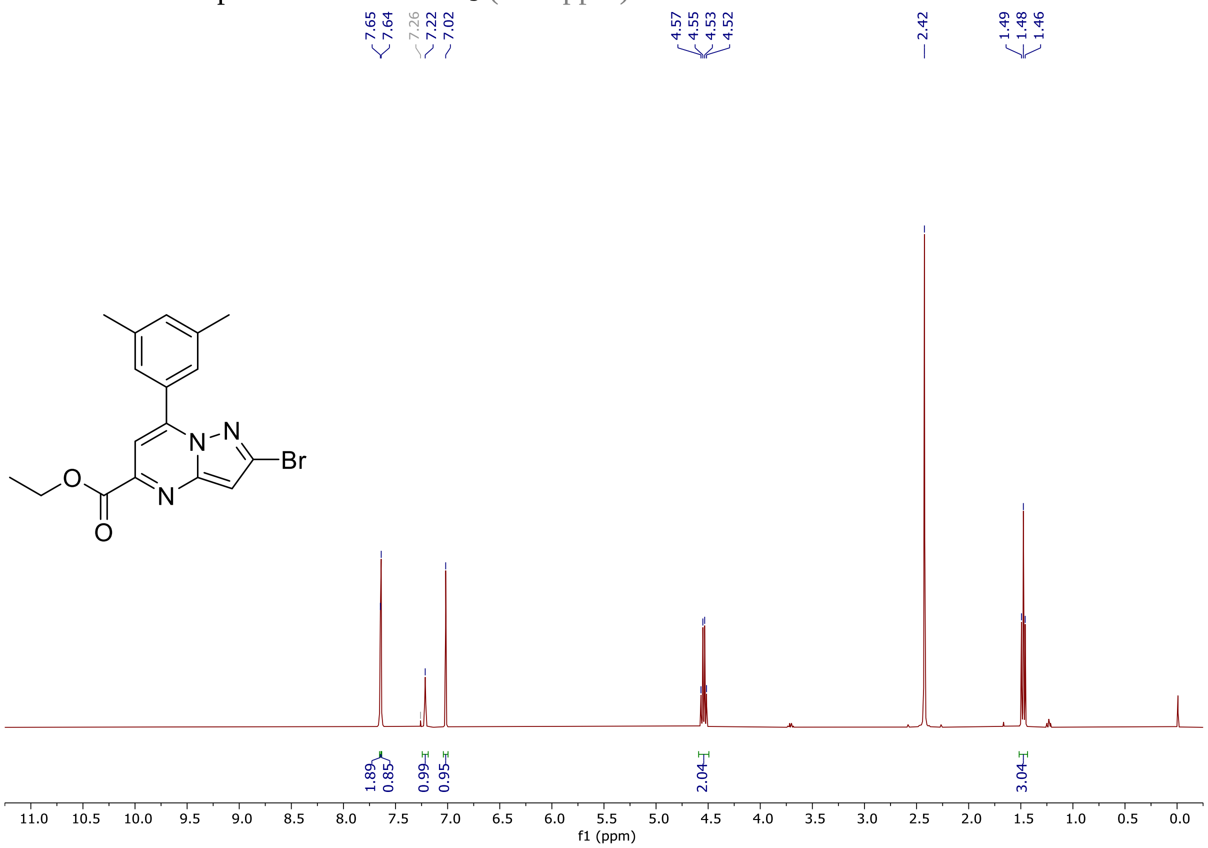

^{13}C NMR of compound **15a** in $CDCl_3$ (77.16 ppm)

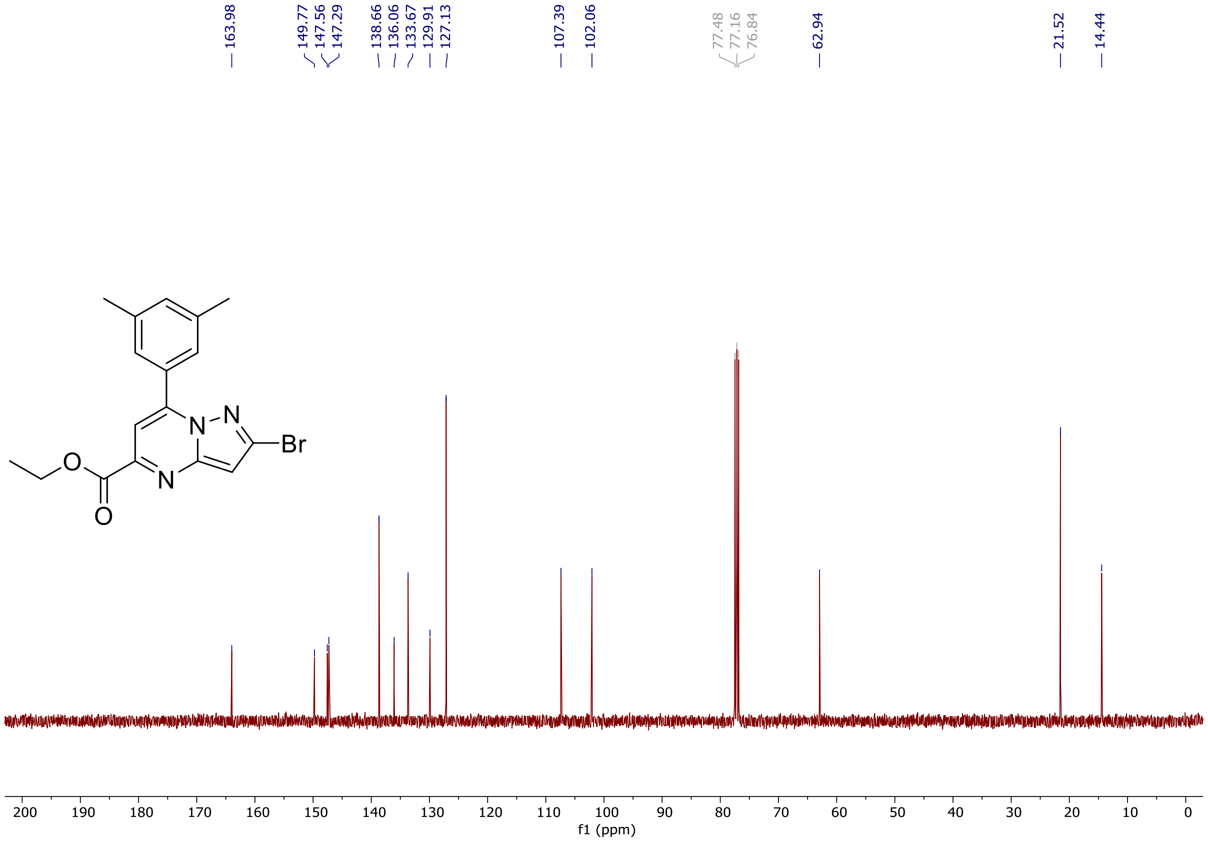

^{1}H NMR of compound **15b** in $CDCl_3$ (7.26 ppm)

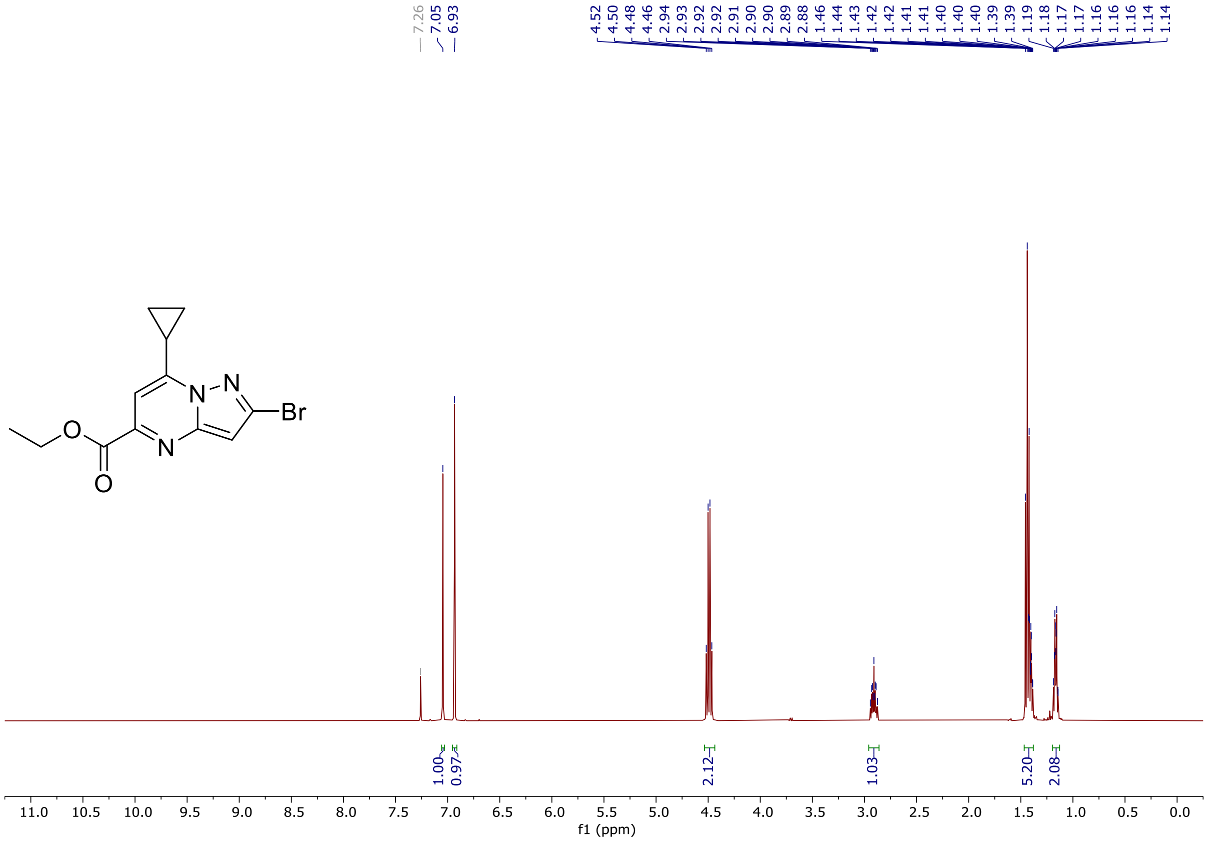

^{13}C NMR of compound **15b** in $CDCl_3$ (77.16 ppm)

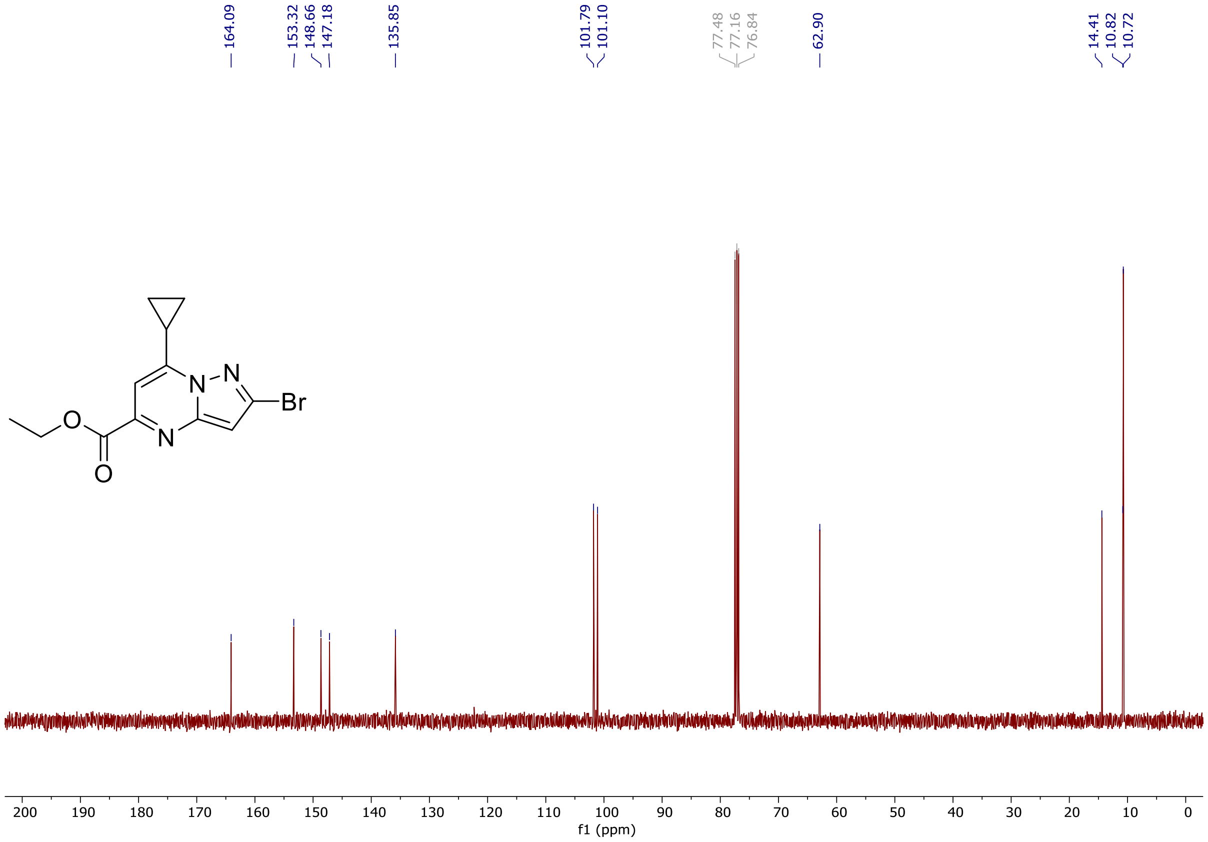

^{1}H NMR of compound **16a** in CD_3OD (3.31 ppm)

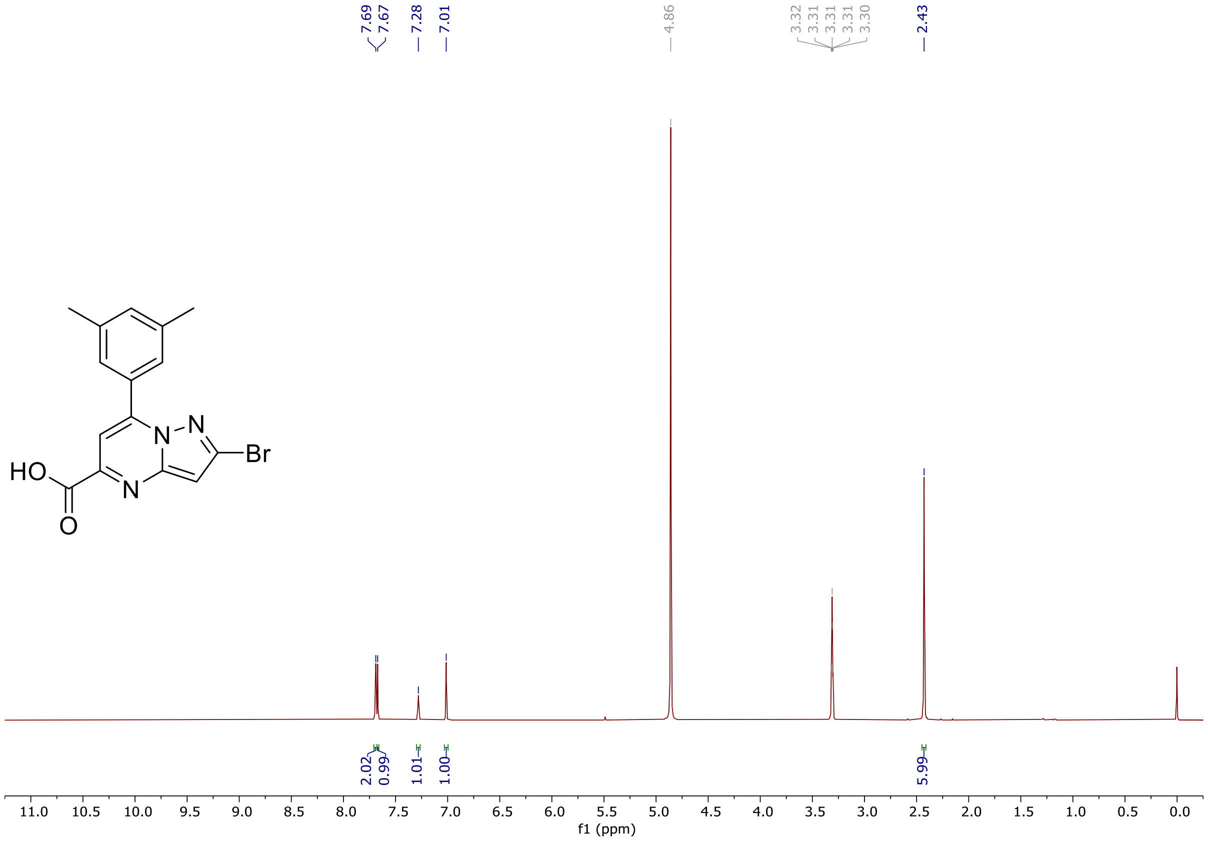

^{13}C NMR of compound **16a** in CD_3OD (49.00 ppm)

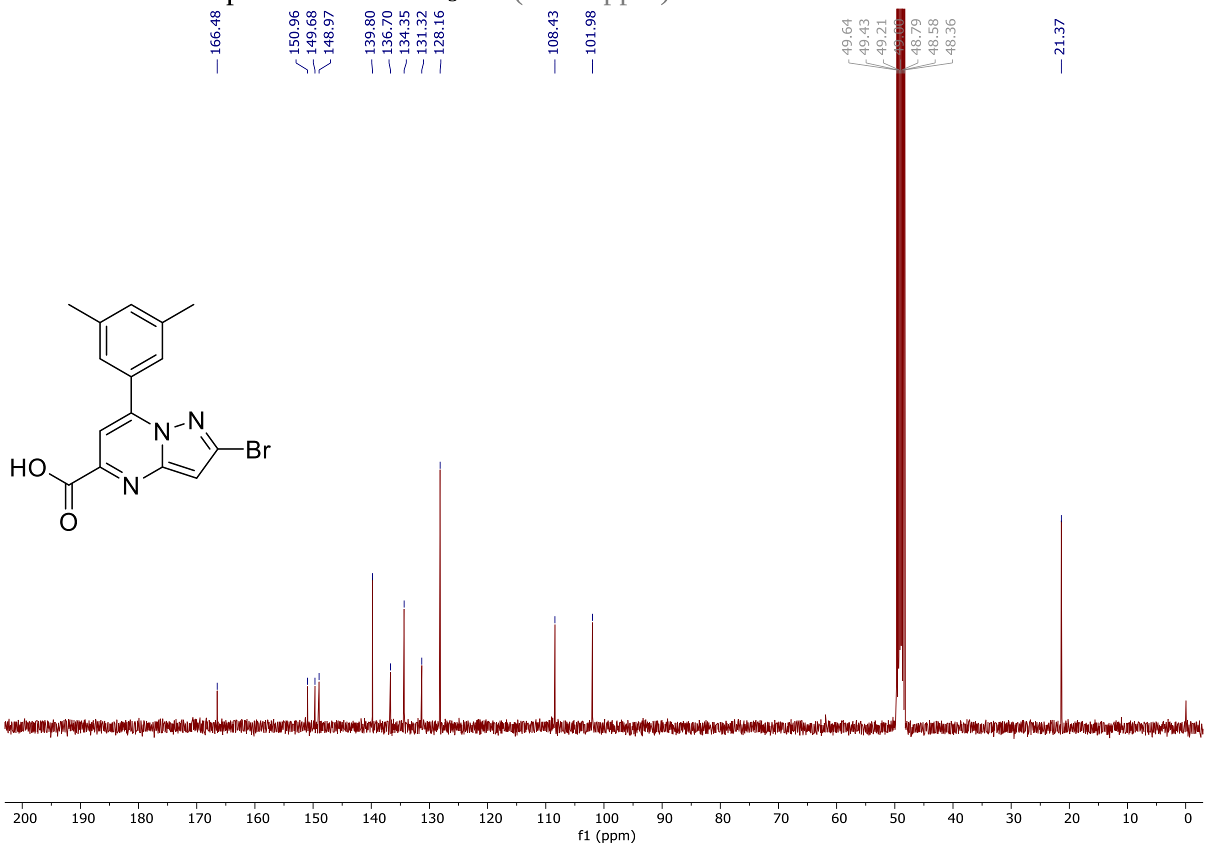

^{1}H NMR of compound **16b** in CD_3OD (3.31 ppm)

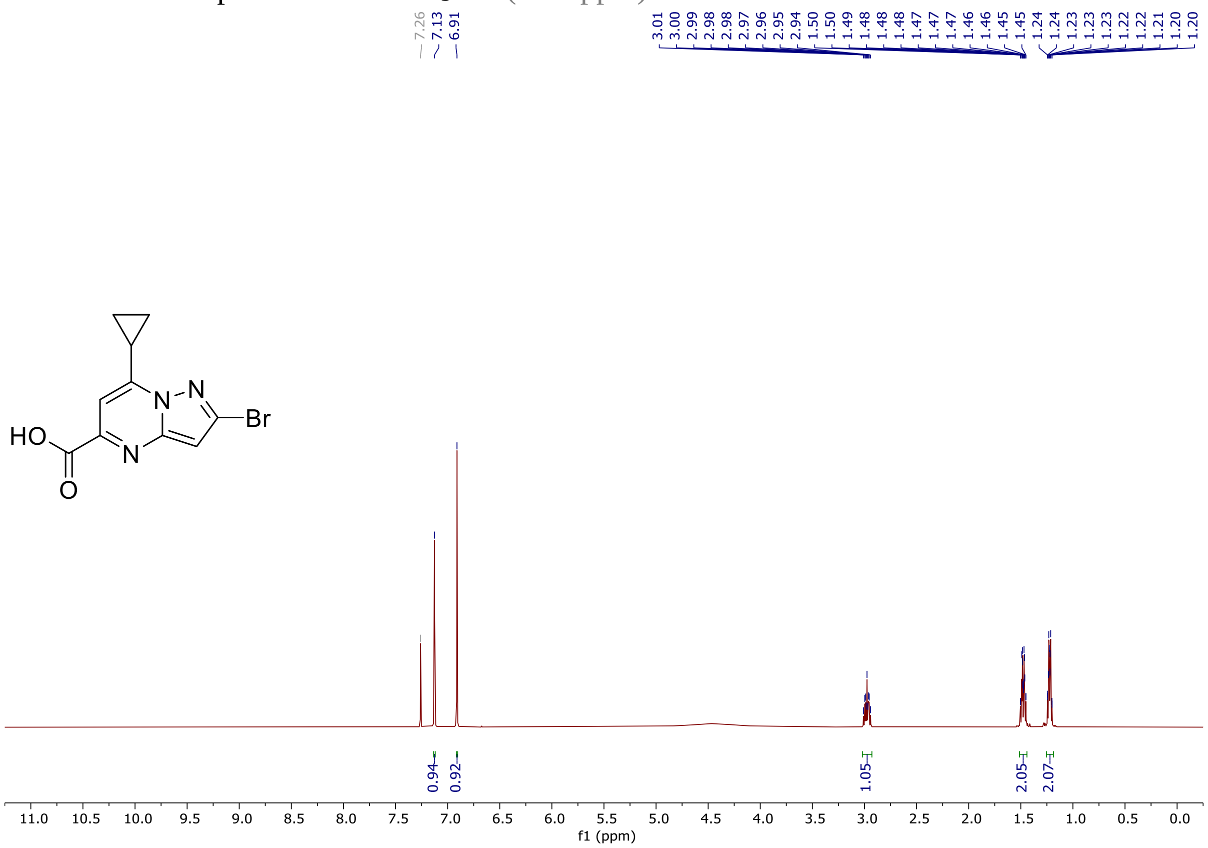

^{13}C NMR of compound **16b** in CD_3OD (49.00 ppm)

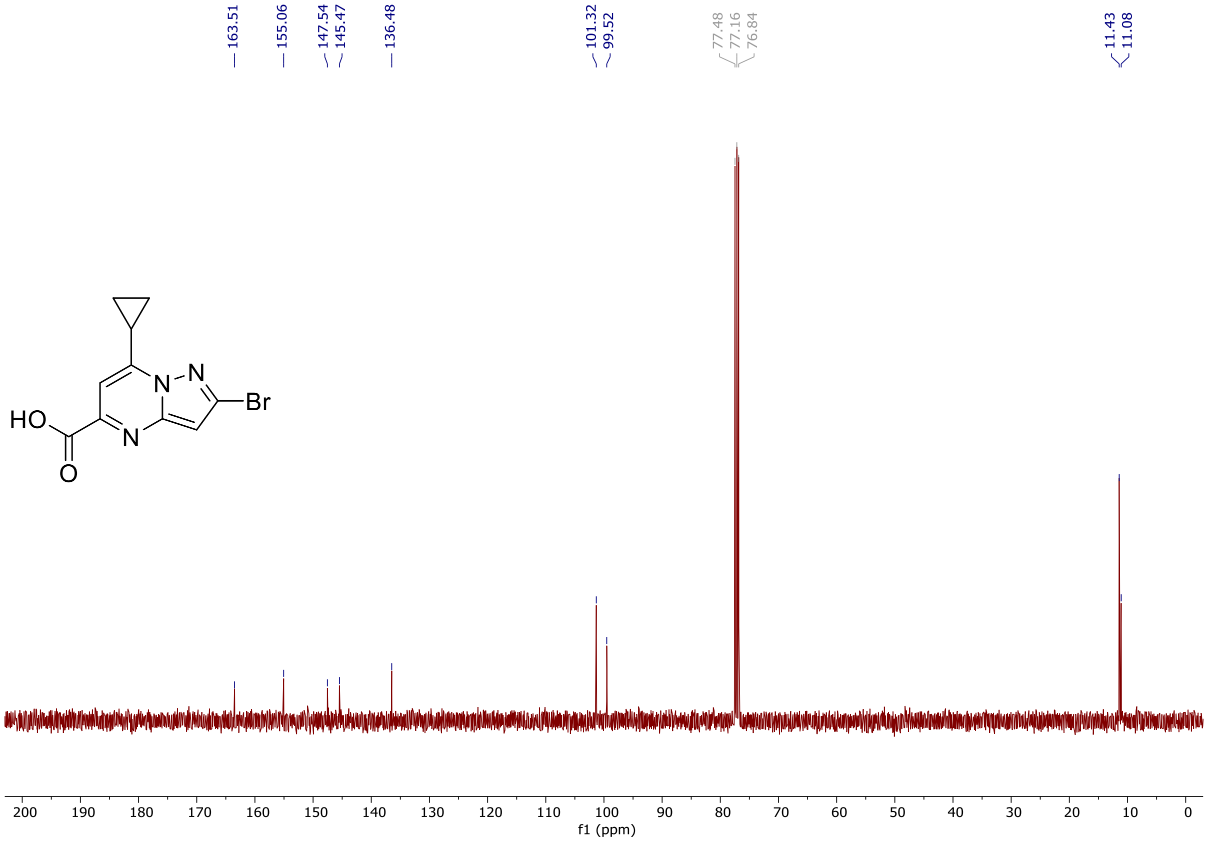

^{1}H NMR of compound **17a** in $CDCl_3$ (7.26 ppm)

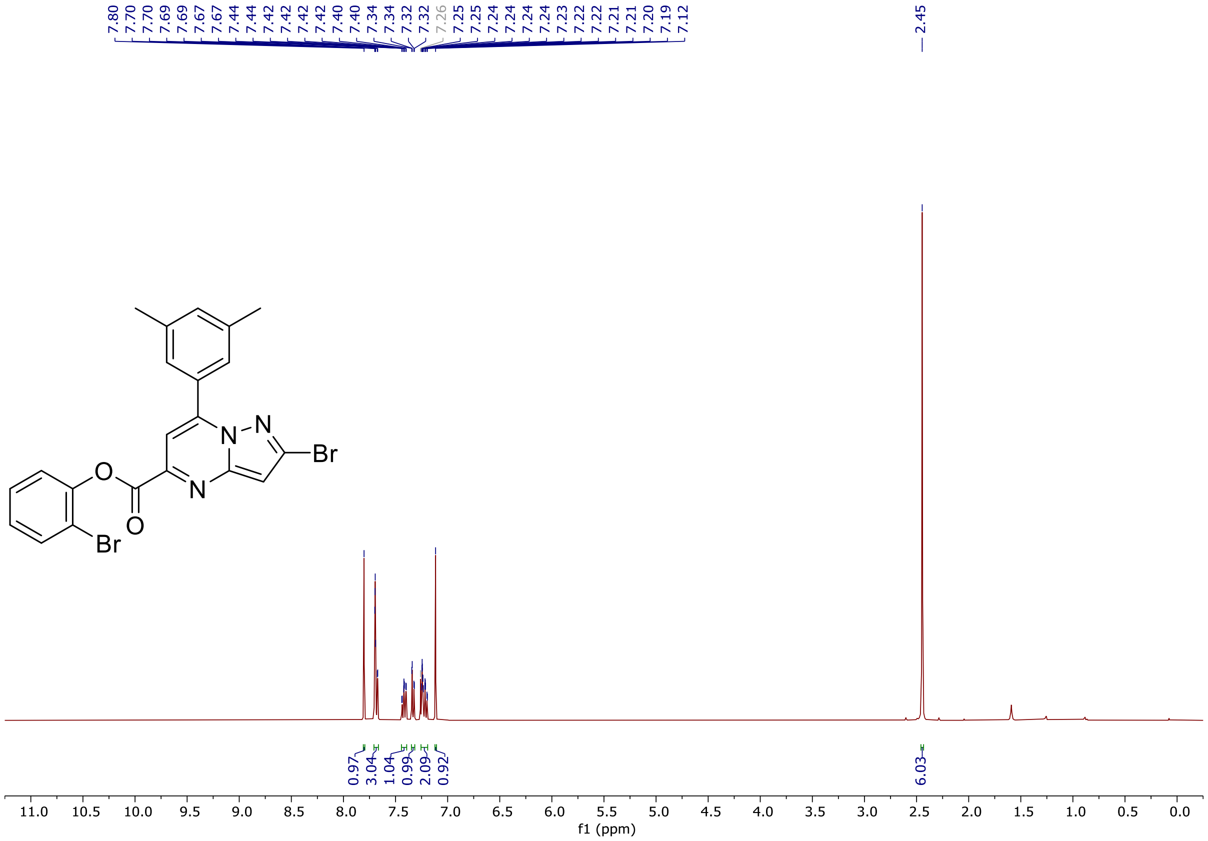

^{13}C NMR of compound **17a** in $CDCl_3$ (77.16 ppm)

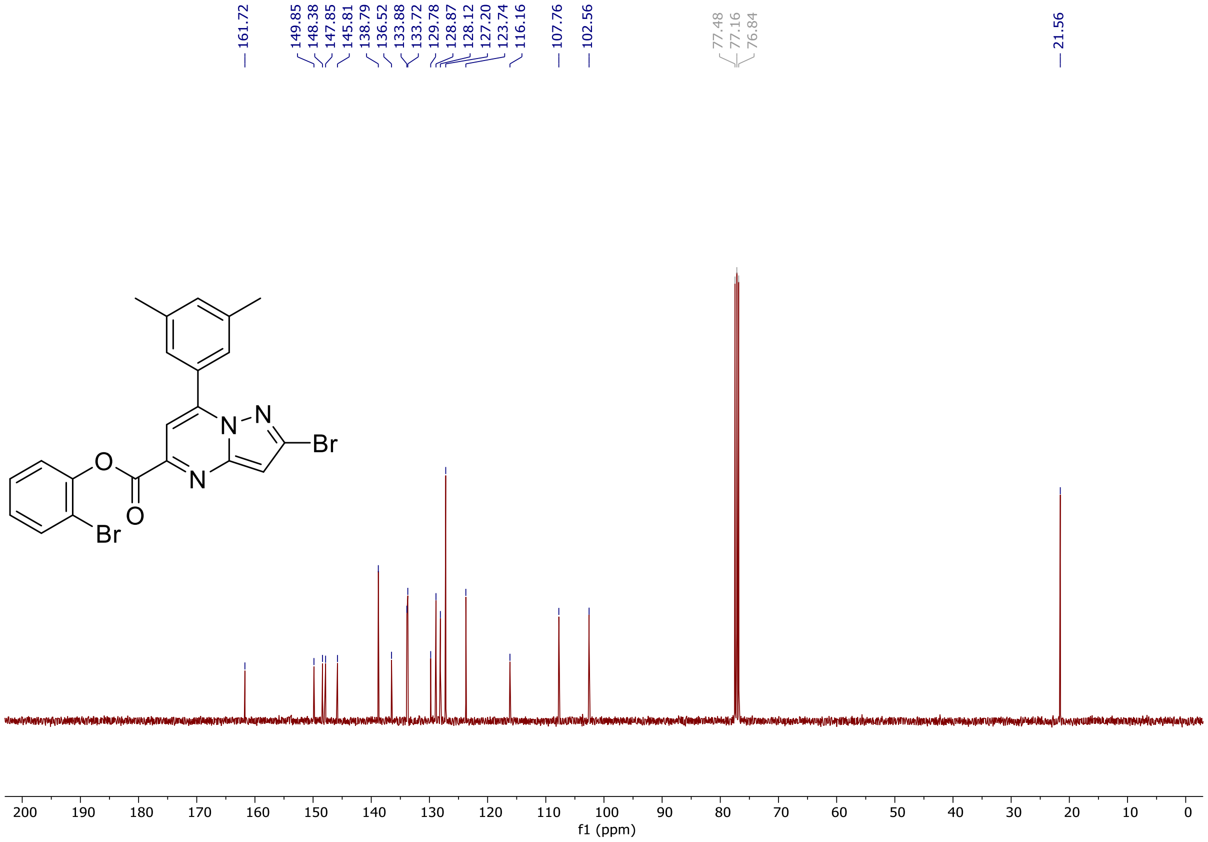

^{1}H NMR of compound **17b** in $CDCl_3$ (7.26 ppm)

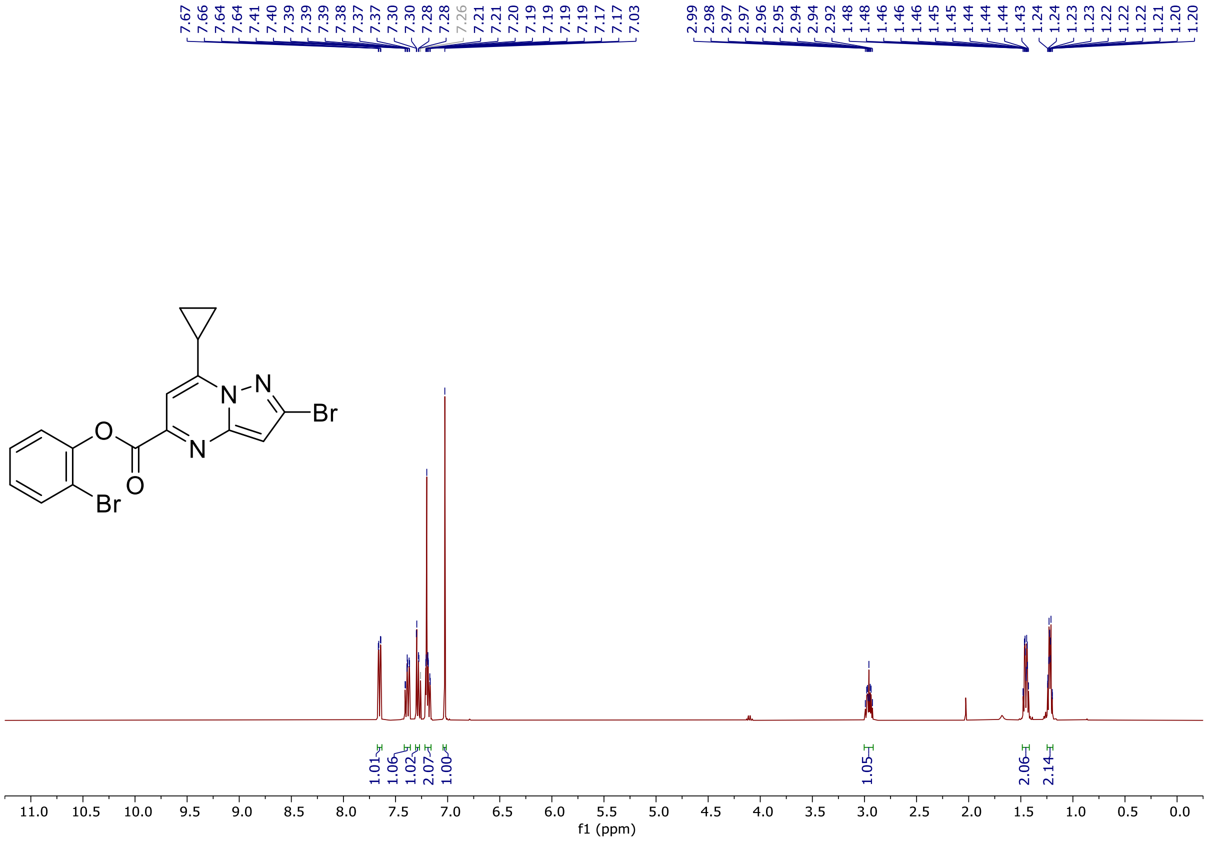

^{13}C NMR of compound **17b** in $CDCl_3$ (77.16 ppm)

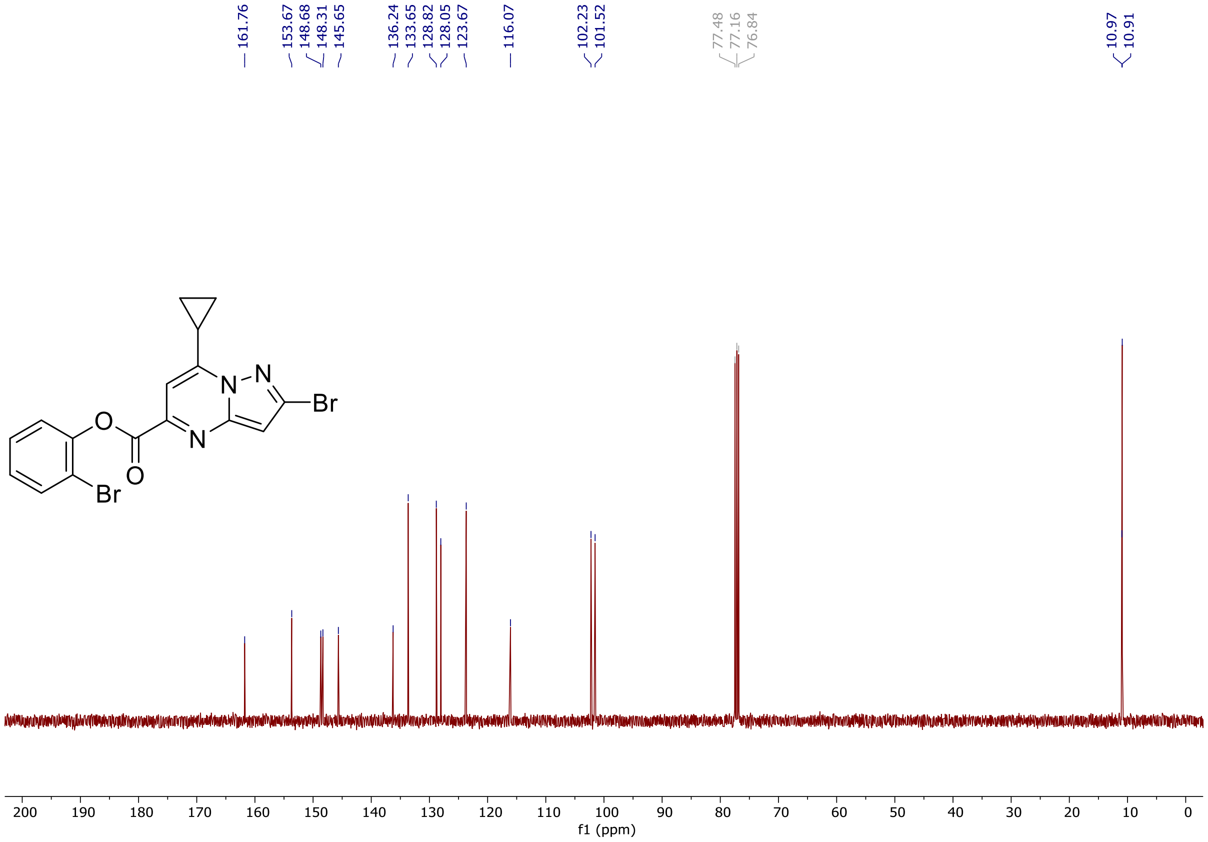

^{1}H NMR of compound **18a** in $CDCl_3$ (7.26 ppm)

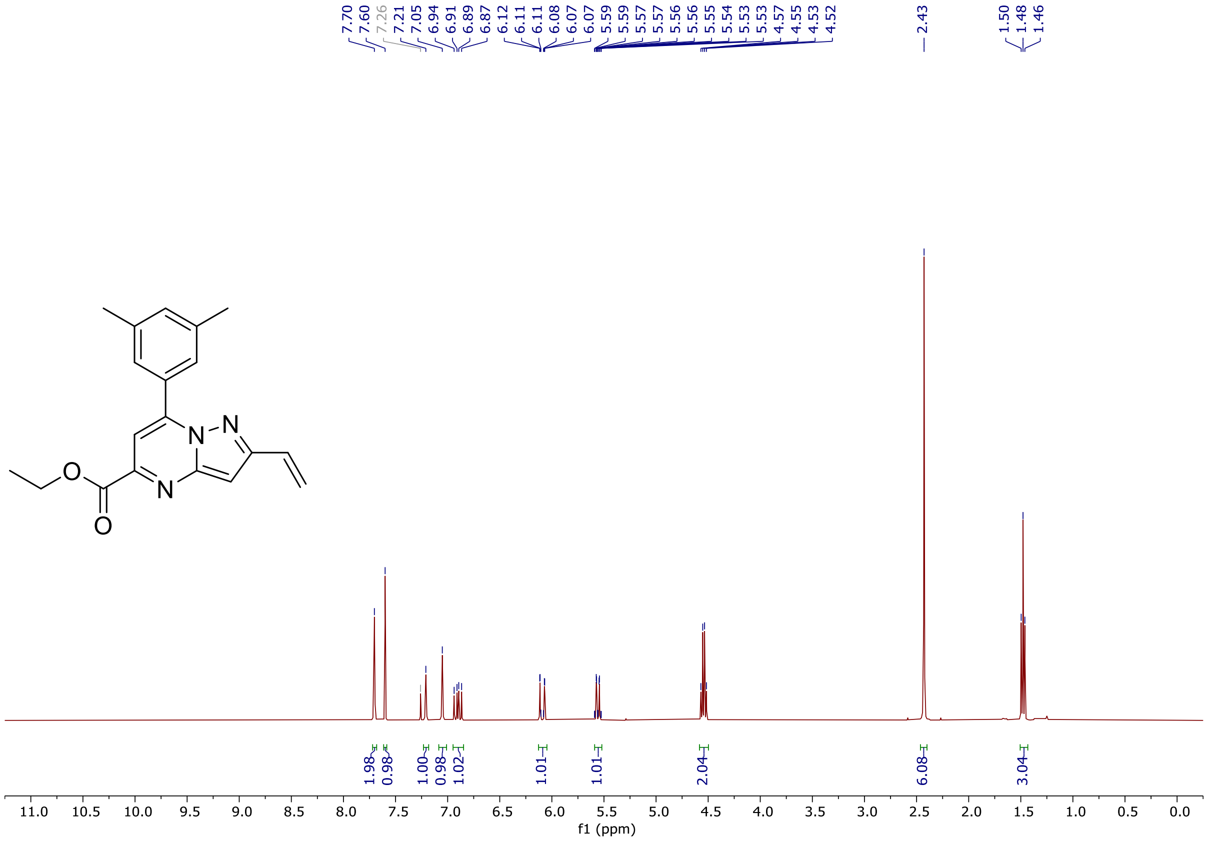

^{13}C NMR of compound **18a** in $CDCl_3$ (77.16 ppm)

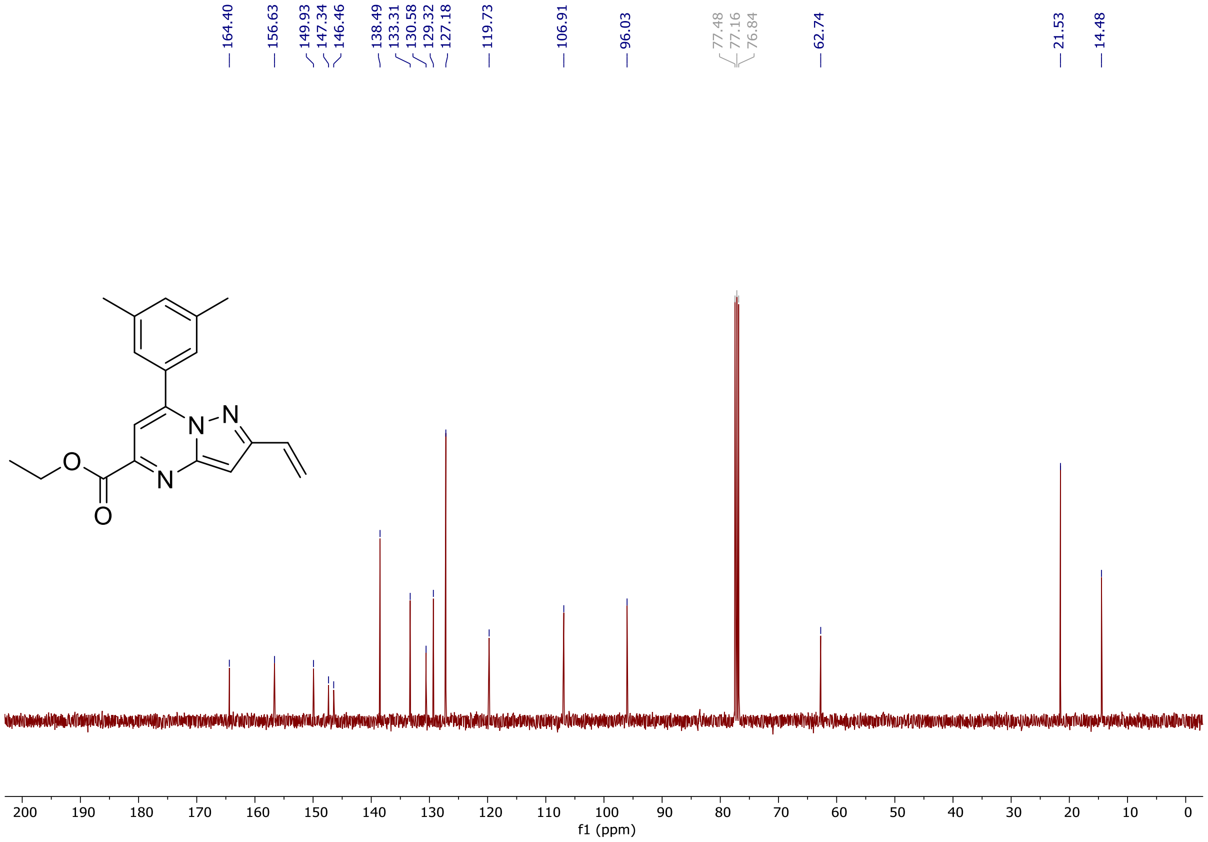

^{1}H NMR of compound **18b** in $CDCl_3$ (7.26 ppm)

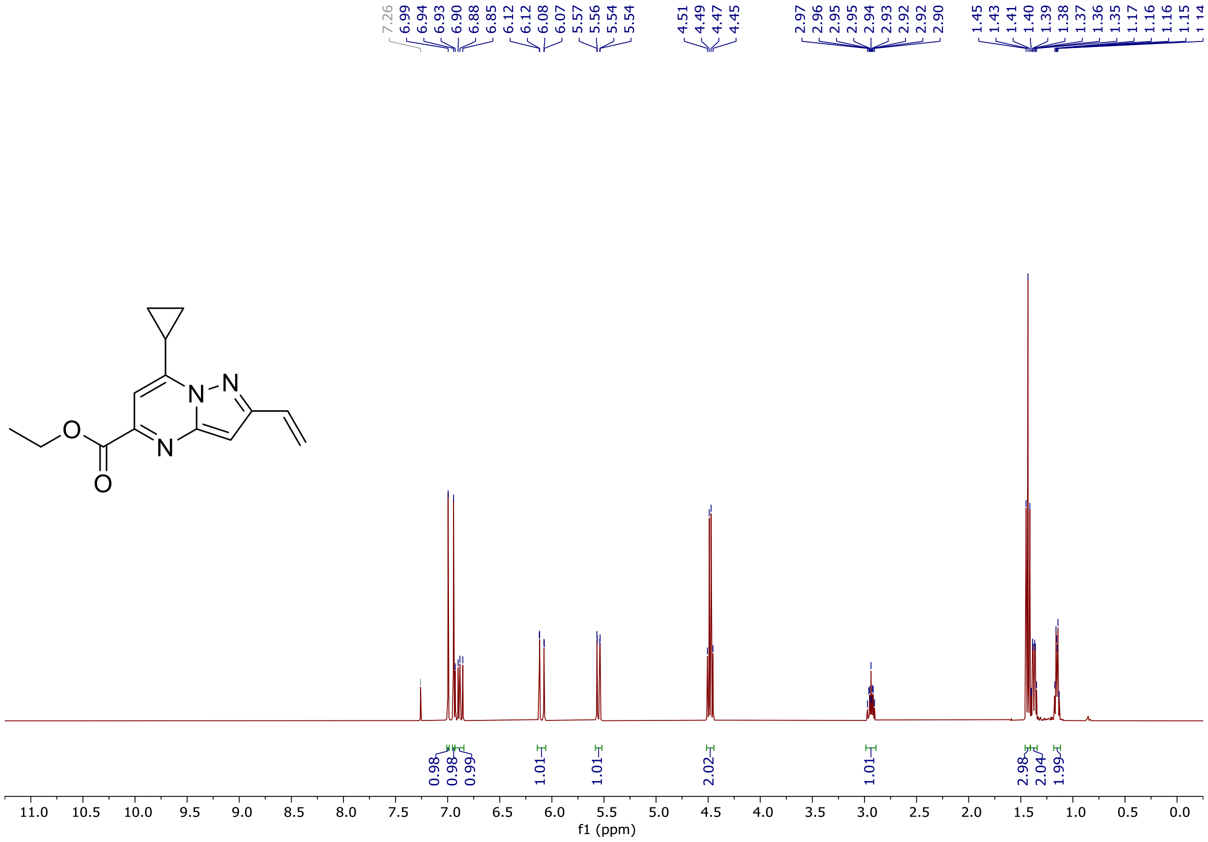

^{13}C NMR of compound **18b** in $CDCl_3$ (77.16 ppm)

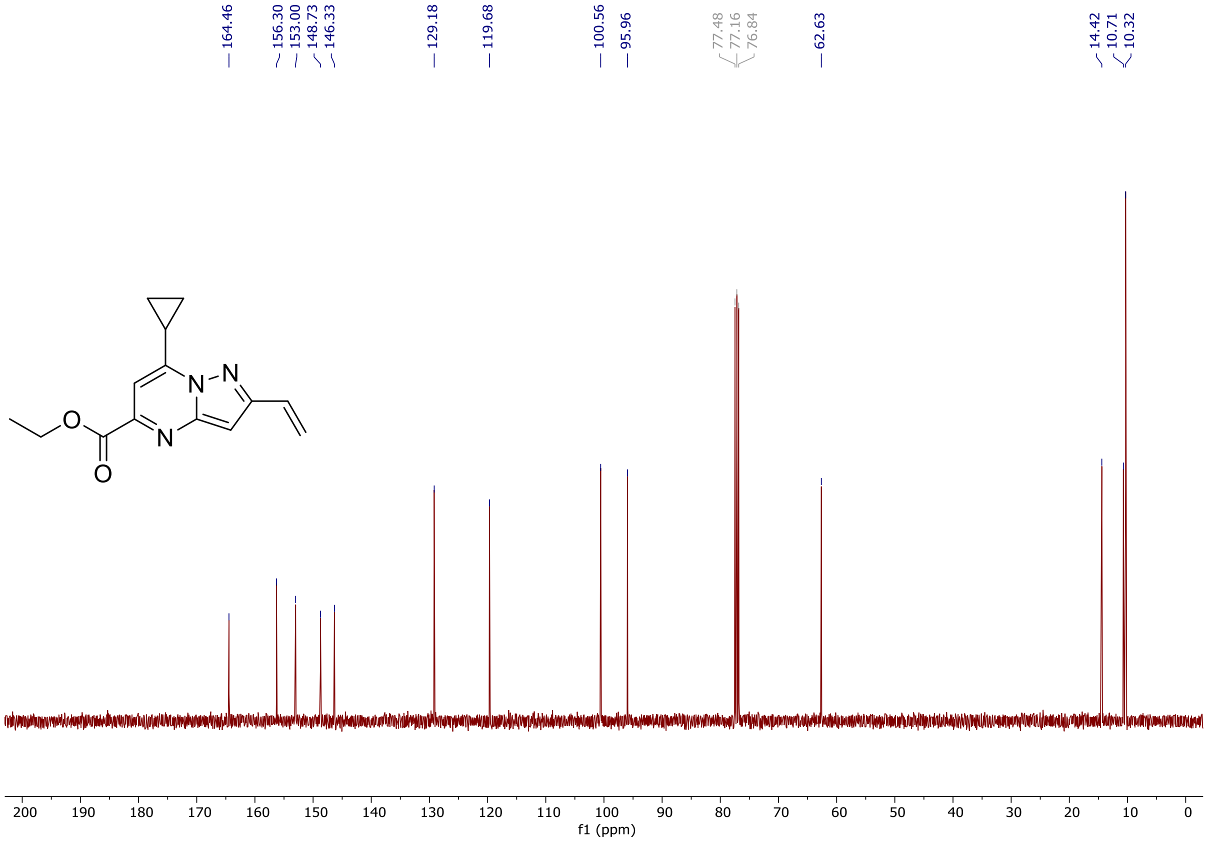

^{1}H NMR of compound **19a** in CD_3OD (3.31 ppm)

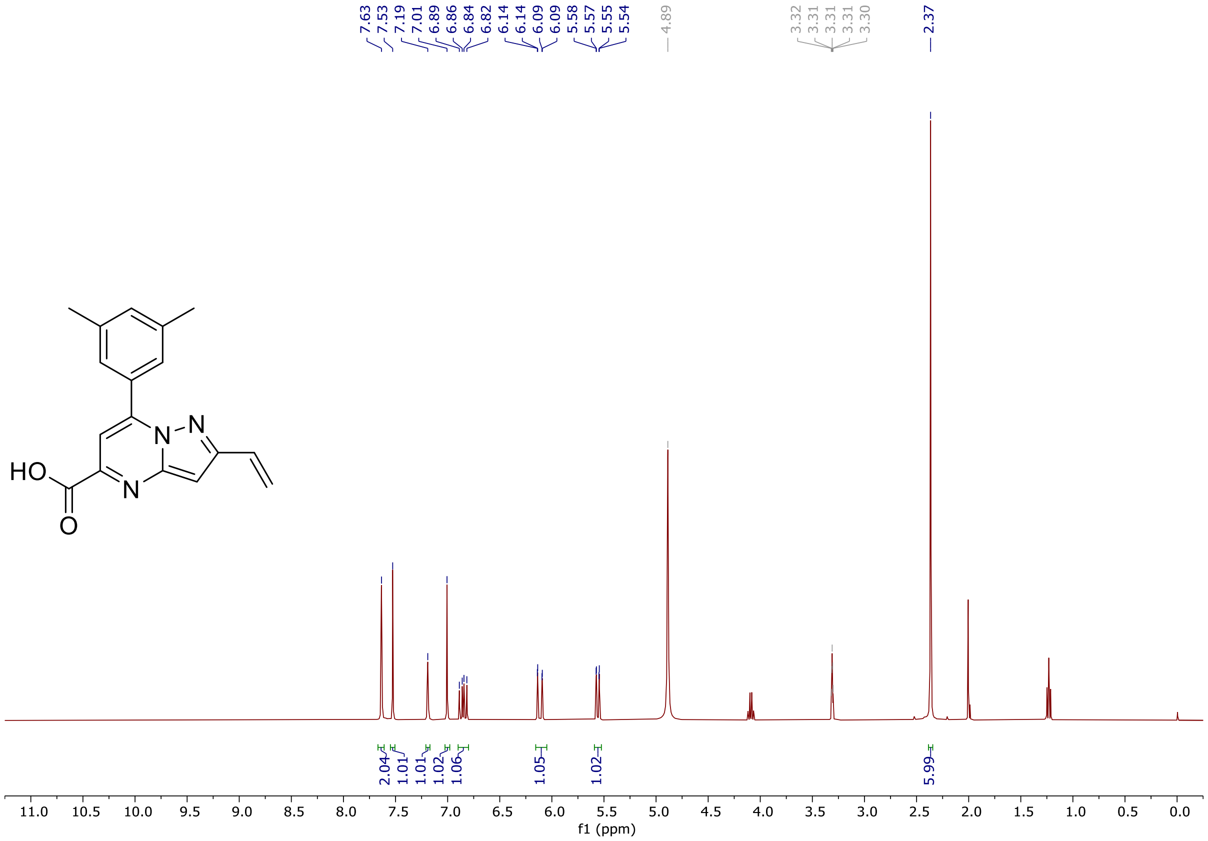

^{13}C NMR of compound **19a** in CD_3OD (49.00 ppm)

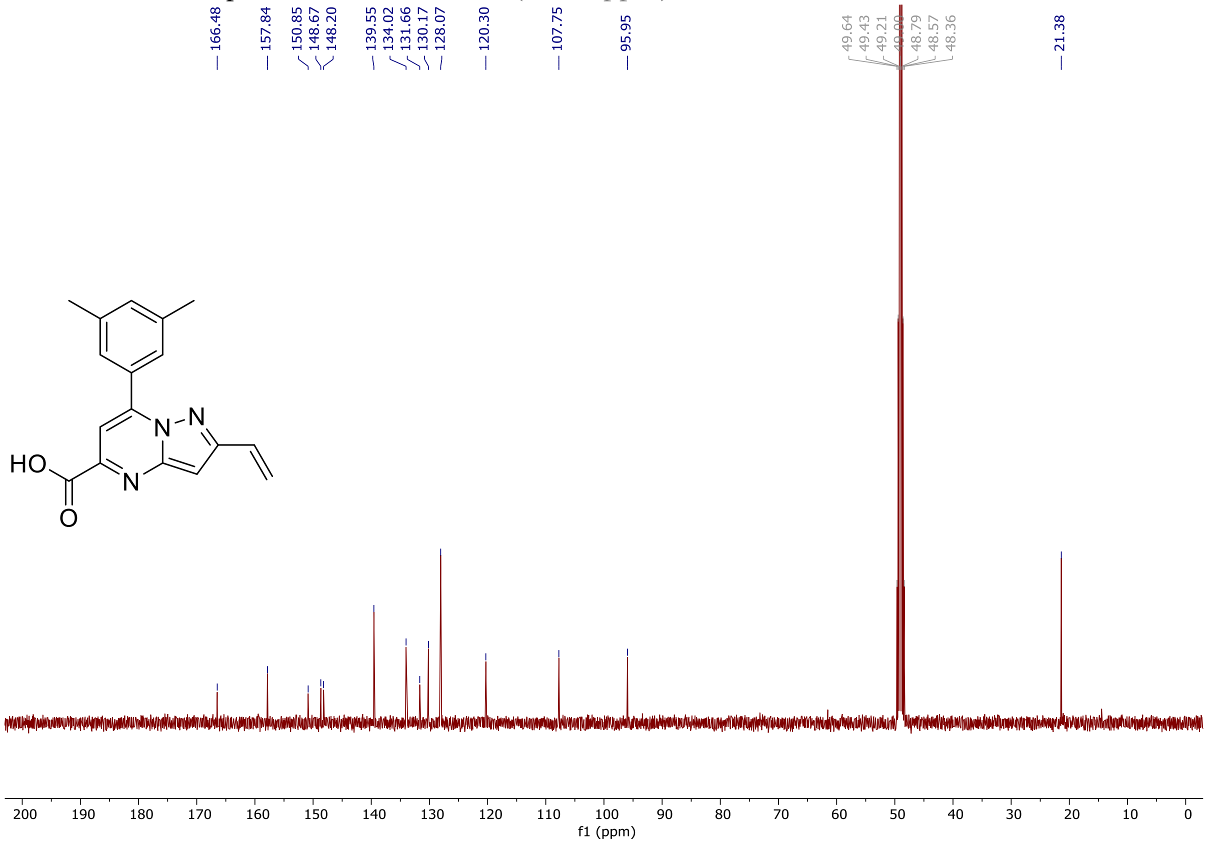

^{1}H NMR of compound **19b** in CD_3OD (3.31 ppm)

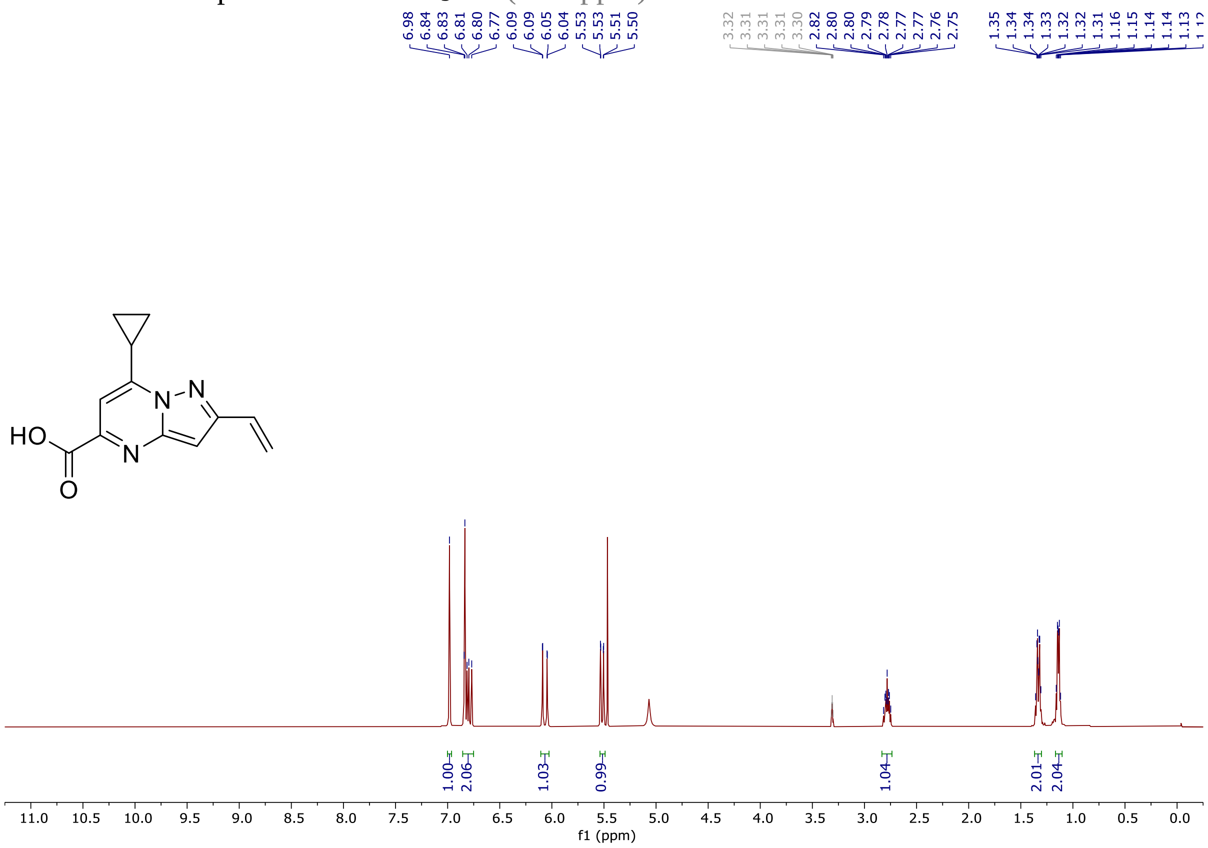

^{13}C NMR of compound **19b** in CD_3OD (49.00 ppm)

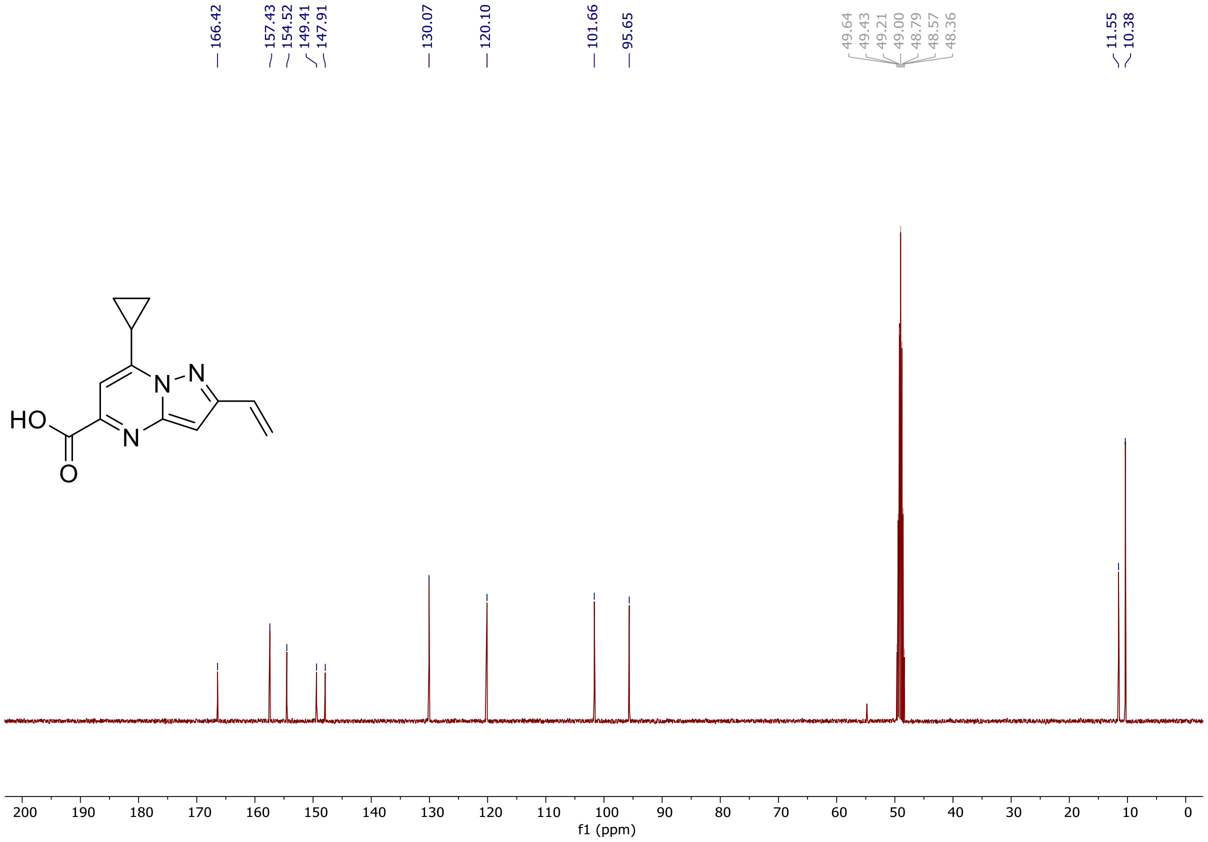

^{1}H NMR of compound **20a** in $CDCl_3$ (7.26 ppm)

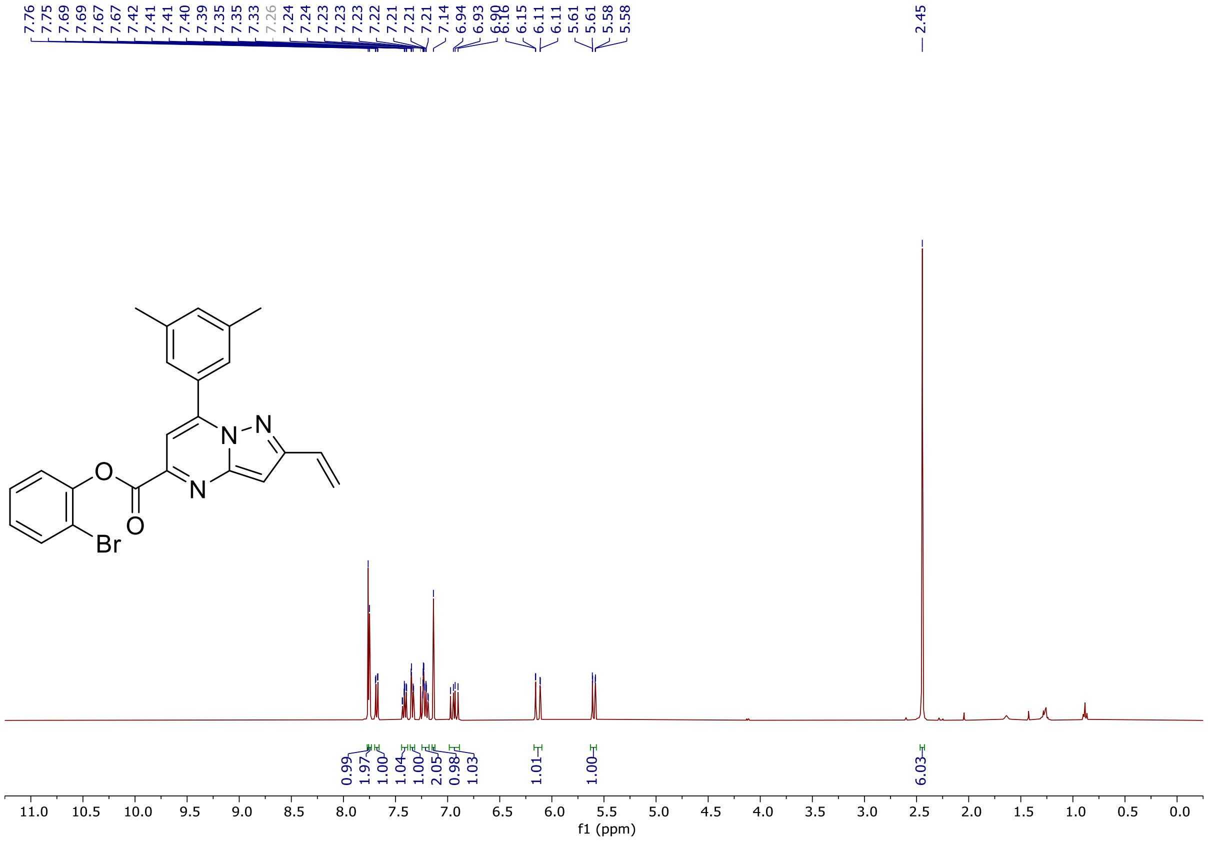

^{13}C NMR of compound **20a** in $CDCl_3$ (77.16 ppm)

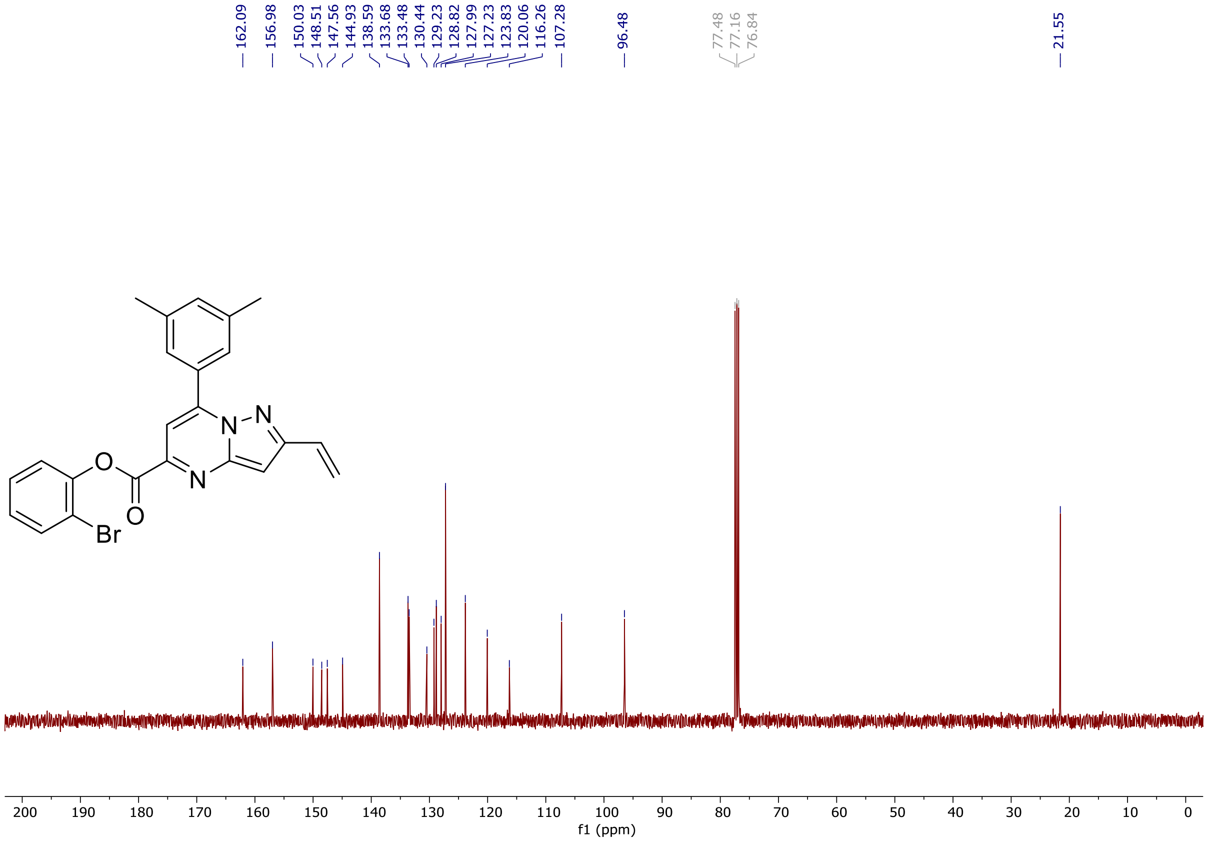

^{1}H NMR of compound **20b** in $CDCl_3$ (7.26 ppm)

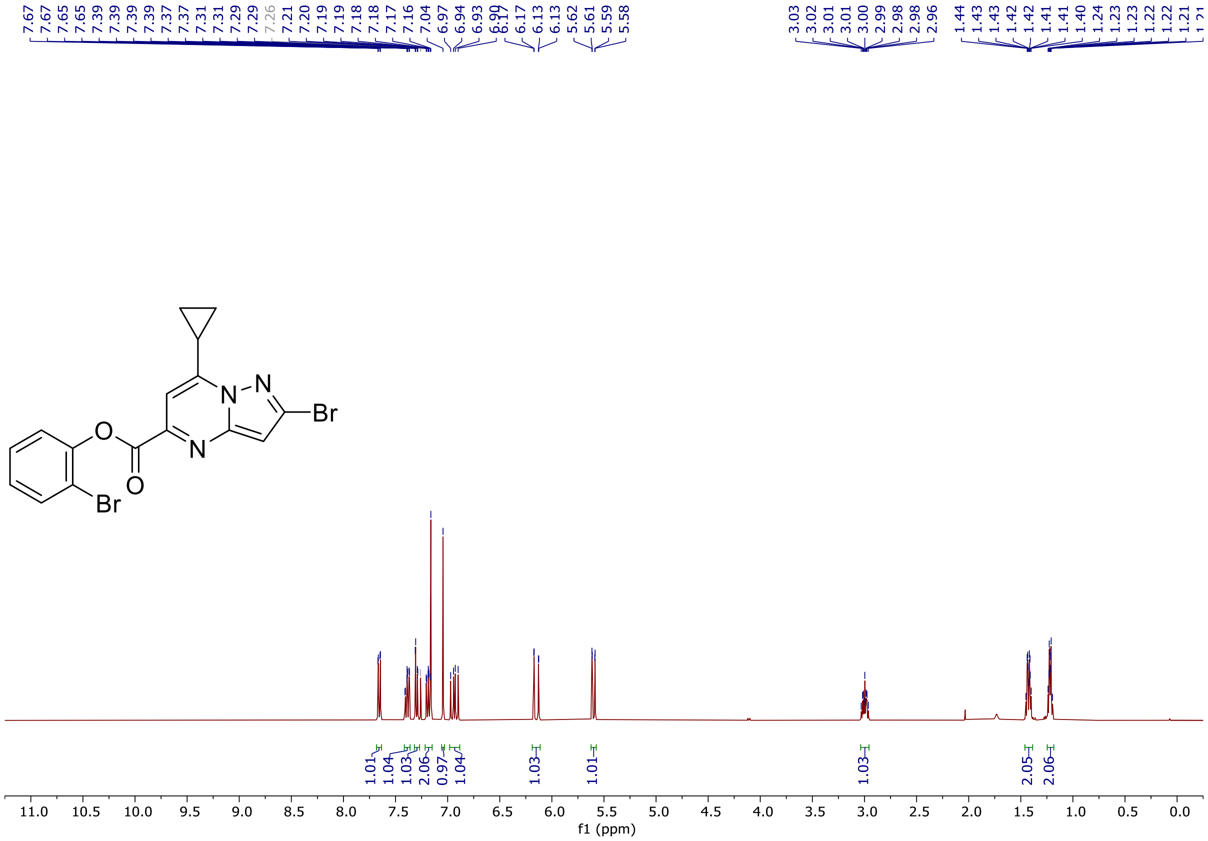

^{13}C NMR of compound **20b** in $CDCl_3$ (77.16 ppm)

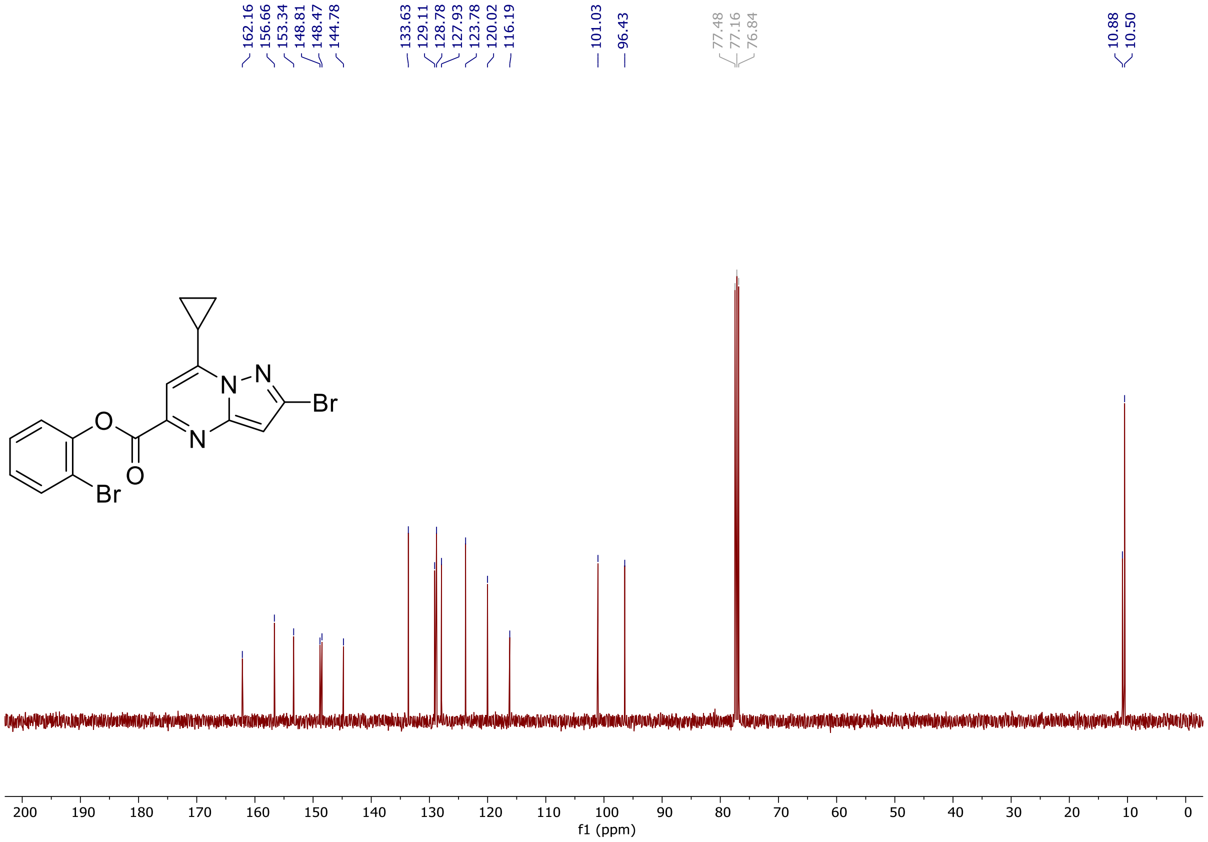

^{1}H NMR of compound **21a** in CD_3OD (3.31 ppm)

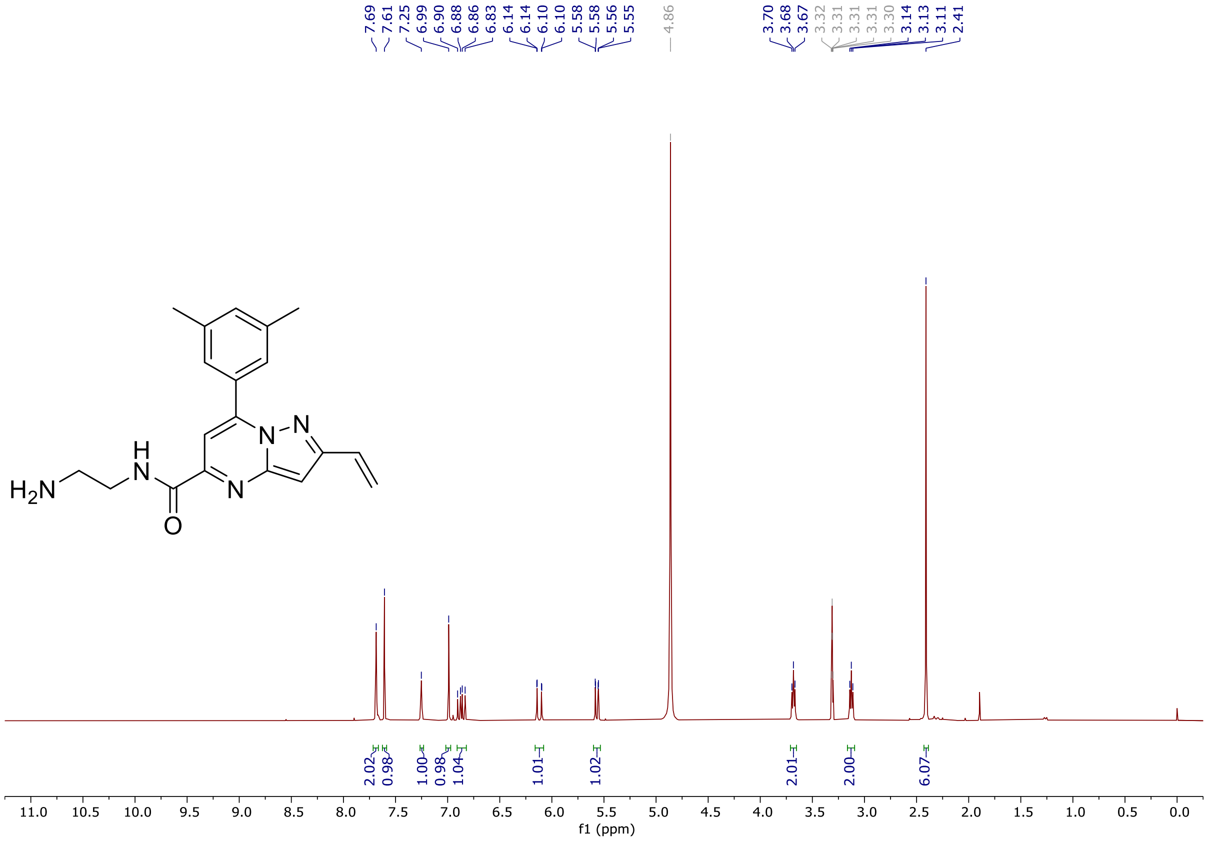

^{13}C NMR of compound **21a** in CD_3OD (49.00 ppm)

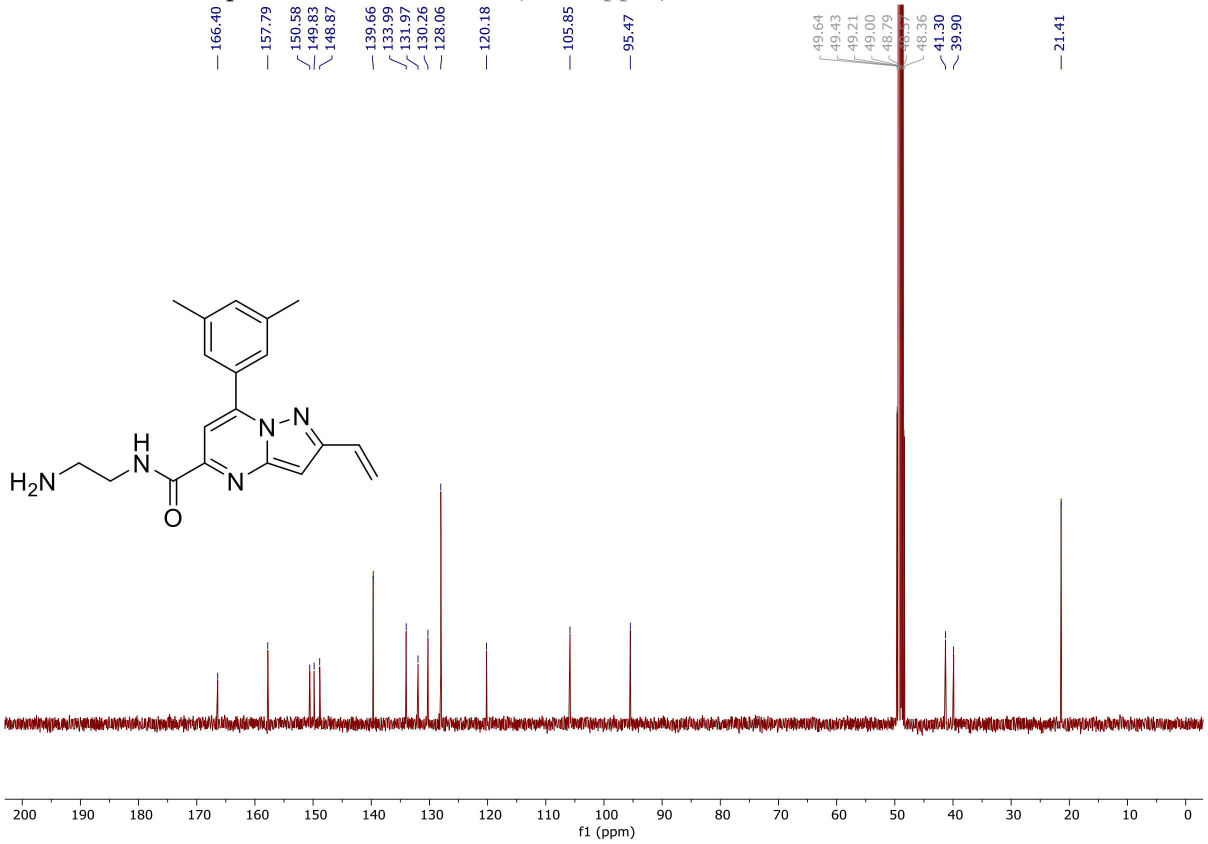

^{1}H NMR of compound **21b** in CD_3OD (3.31 ppm)

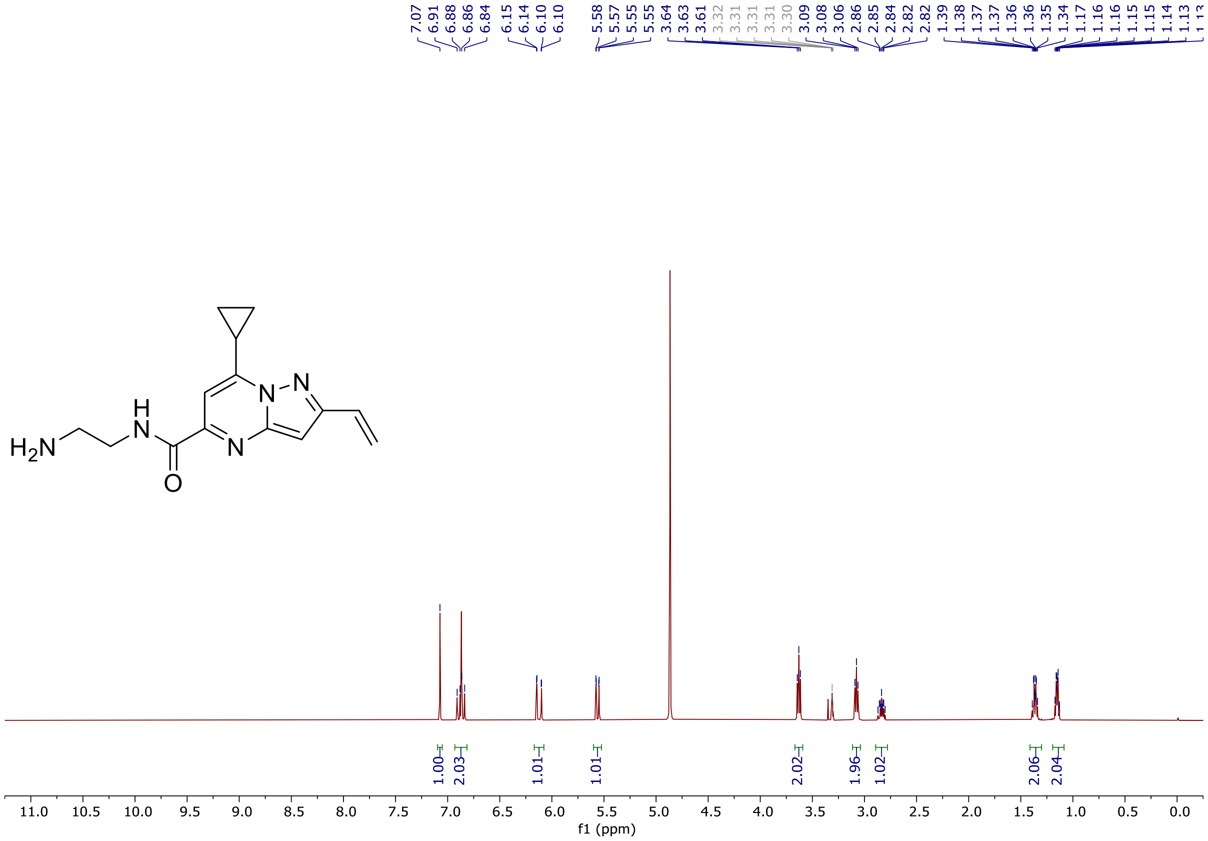

^{13}C NMR of compound **21b** in CD_3OD (49.00 ppm)

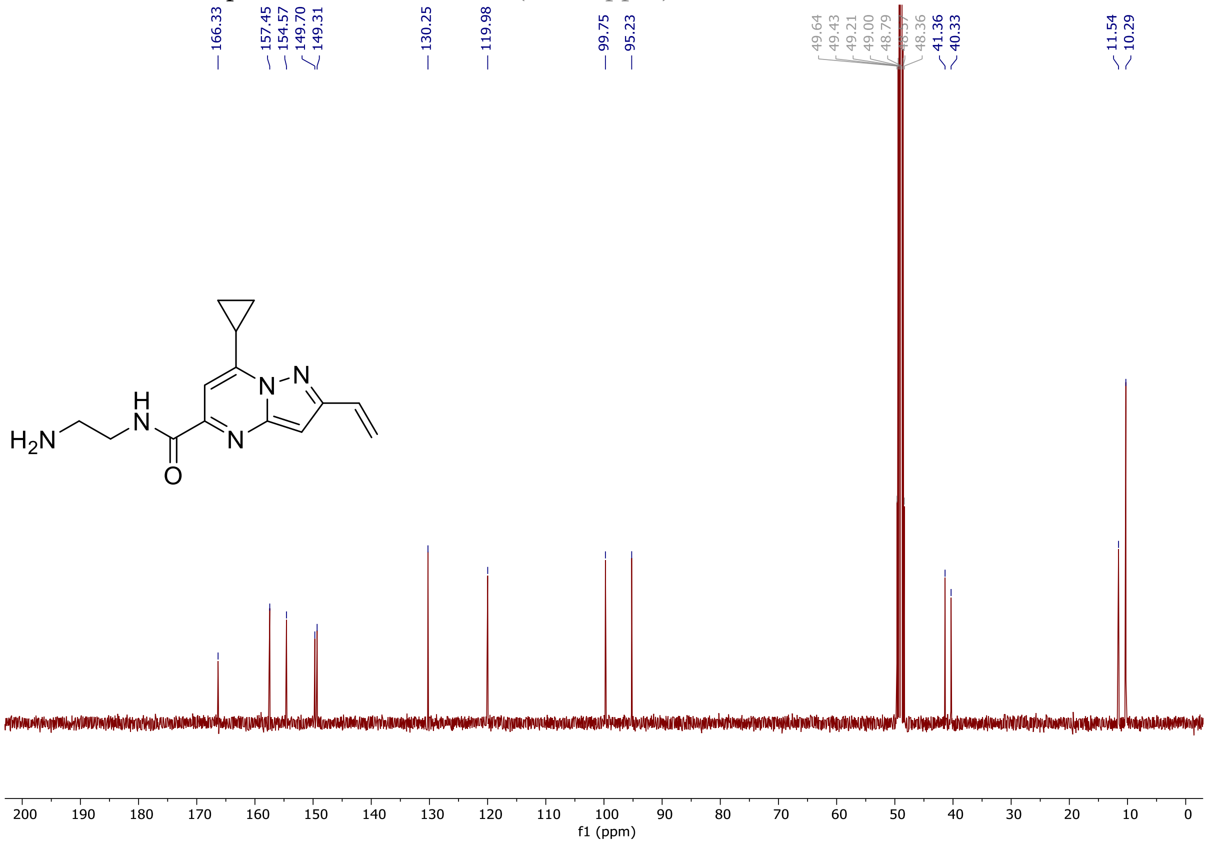

8 Mass spectra (for 17a, 19a and 20a)

Compound **17a**

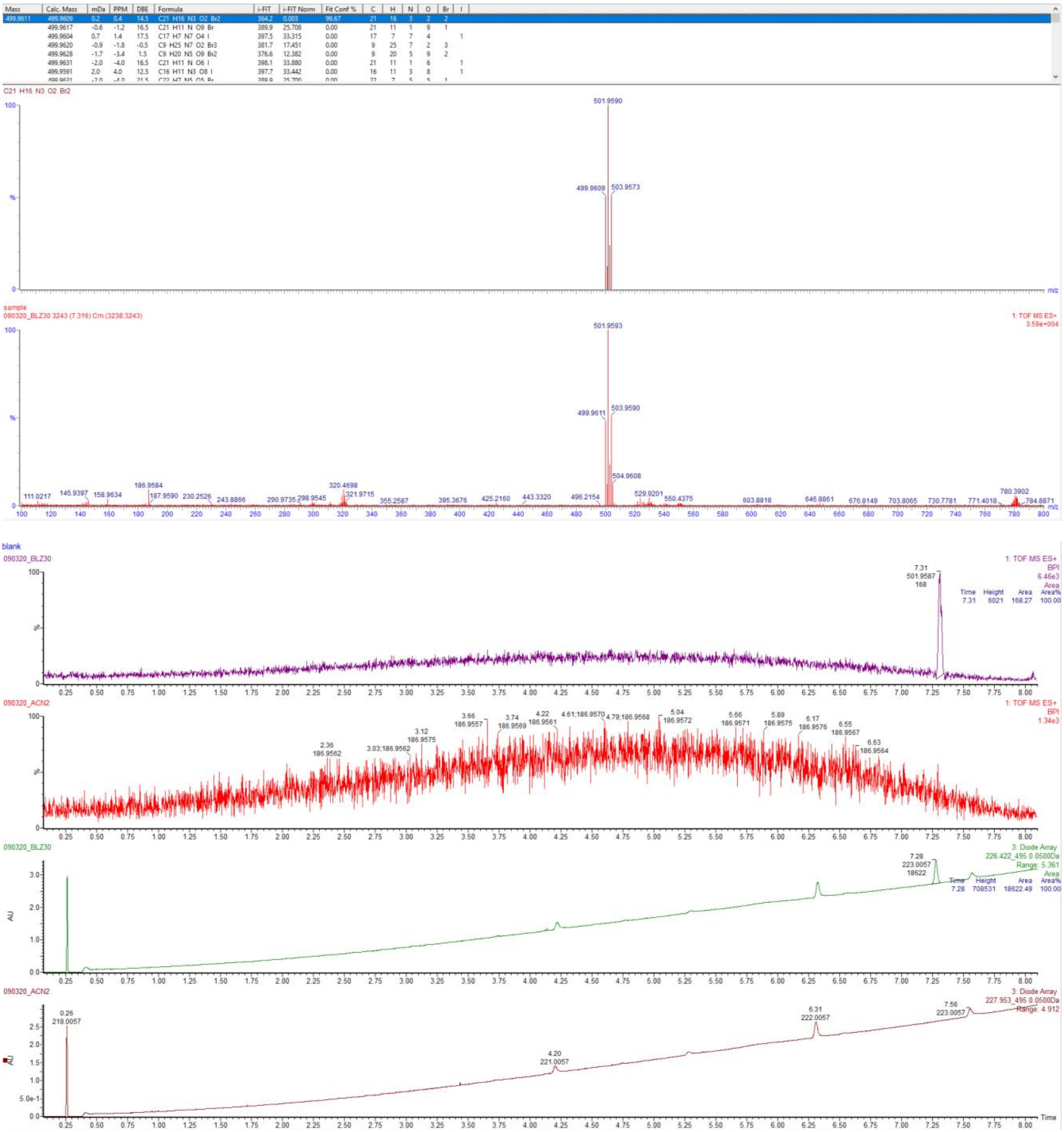

Compound **19a**

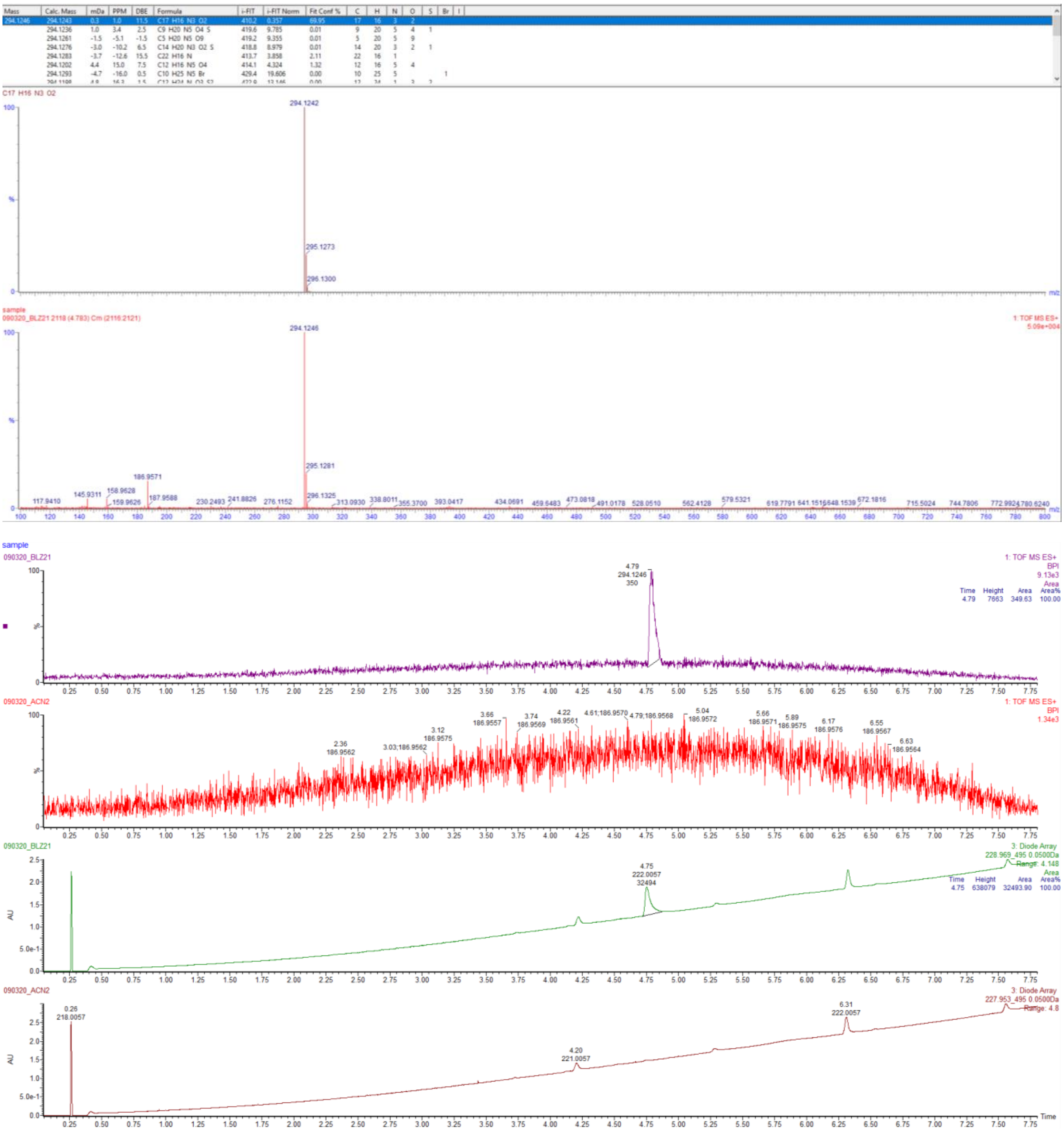

Compound **20a**

Mass	Calc. Mass	mDa	PPM	DBE	Formula	i-FIT	i-FIT Norm	Fit Conf %	C	H	N	O	S	Br	I
448.0665	448.0664	0.1	0.2	-1.5	C8 H26 N5 O8 S4	558.4	34.342	0.00	8	26	5	8	4		
	448.0663	0.2	0.4	0.5	C15 H31 N S3 I	558.6	34.607	0.00	15	31	1		3		1
	448.0668	-0.3	-0.7	6.5	C15 H23 N5 O S I	552.9	28.901	0.00	15	23	5	1	1		1
	448.0669	-0.4	-0.9	17.5	C23 H14 N O9	560.7	36.707	0.00	23	14	1	9			
	448.0661	0.4	0.9	15.5	C23 H19 N3 O2 Br	524.0	0.000	99.98	23	19	3	2		1	
	448.0671	-0.6	-1.3	0.5	C11 H28 N7 O2 Br2	560.2	36.212	0.00	11	28	7	2		2	
	448.0671	-0.6	-1.3	7.5	C16 H22 N3 O6 S3	557.4	33.408	0.00	16	22	3	6	3		
	448.0657	0.8	1.8	26.5	C28 H10 N5 S	554.9	30.826	0.00	28	10	5		1		

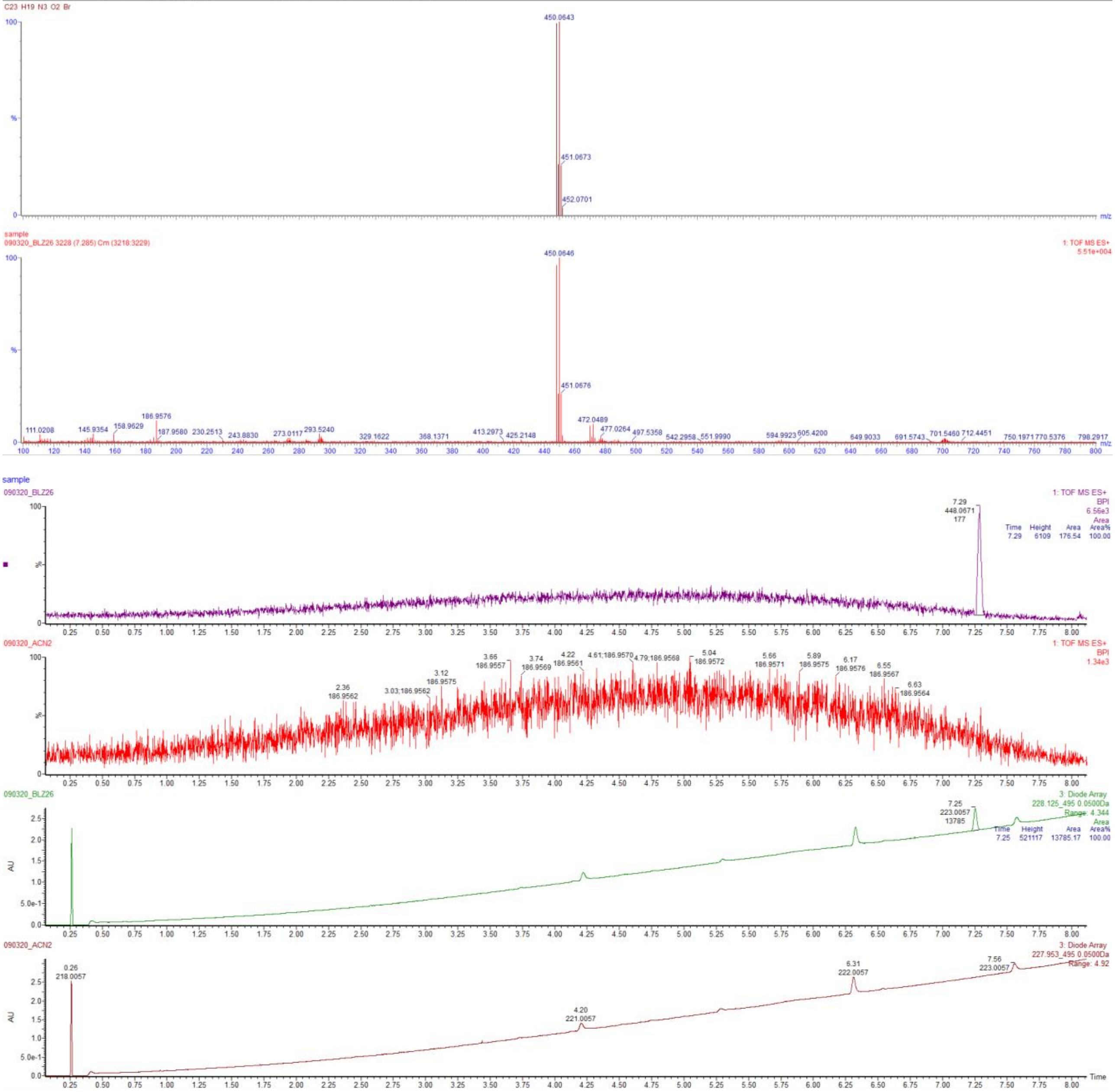

References

[1] Schrödinger: Small-Molecule Drug Discovery Suite **2019-4**. Schrödinger, LLC: New York **2019**.

[2] R Core Team. R: A Language and Environment for Statistical Computing. R Foundation for Statistical Computing, **2019**.

[3] F. Commo, B. M. Bot, Nplr v0.1-7. **2016**.

[4] H. J. Motulsky, R. E. Brown, *BMC Bioinformatics* **2006**, *7*, 123.
